# Tackling social disconnection: an umbrella review of RCT-based interventions targeting social isolation and loneliness

**DOI:** 10.1186/s12889-024-19396-8

**Published:** 2024-07-17

**Authors:** Thomas Hansen, Ragnhild Bang Nes, Kamila Hynek, Thomas Sevenius Nilsen, Anne Reneflot, Kim Stene-Larsen, Ragnhild Agathe Tornes, Julia Bidonde

**Affiliations:** 1 Department of Mental Health and Suicide, Norwegian Institute of Public Health, Oslo, Norway; 2 Promenta Research Center, Department of Psychology, University of Oslo, Oslo, Norway; 3 School of Rehabilitation Science, University of Saskatchewan, Saskatoon, Canada

## Abstract

**Background:**

Social isolation and loneliness are urgent public health concerns associated with negative physical and mental health outcomes. Understanding effective remedies is crucial in addressing these problems. This umbrella review aimed to synthesize and critically appraise scientific evidence on the effectiveness of social isolation and loneliness interventions overall and across subgroups. We focused on systematic reviews (SRs) of randomized controlled trials (RCTs).

**Methods:**

We searched seven databases (June 2022 and updated June 2023) and supplemented the search with grey literature and reference screening to identify SRs published since 2017. Screening, data extraction, and quality assessment using the AMSTAR2 tool were conducted independently by author pairs, with disagreements resolved through discussion.

**Results:**

We included 29 SRs, 16 with meta-analysis and 13 with narrative synthesis. All SRs focused on loneliness, with 12 additionally examining social isolation. Four SRs focused on young people, 11 on all ages, and 14 on older adults. The most frequently examined intervention types were social (social contact, social support), psychological (therapy, psychoeducation, social skills training), and digital (e.g., computer use and online support). Meta-analyses indicated small-to-moderate beneficial effects, while narrative synthesis demonstrated mixed or no effect. Social interventions for social isolation and psychological interventions for loneliness were the most promising. However, caution is warranted due to the effects’ small magnitude, significant heterogeneity, and the variable quality of SRs. Digital and other interventions showed mixed or no effect; however, caution is advised in interpreting these results due to the highly diverse nature of the interventions studied.

**Conclusions:**

This overview of SRs shows small to moderate effectiveness of social interventions in reducing social isolation and psychological ones in tackling loneliness. Further rigorously conducted RCTs and SRs are needed to guide policy decisions regarding the implementation of efficacious and scalable interventions. Evaluation should focus on both preventive structural interventions and tailored mitigating strategies that address specific types and causes of loneliness.

**Supplementary Information:**

The online version contains supplementary material available at 10.1186/s12889-024-19396-8.

## Introduction

Social isolation and loneliness (SIL) are pervasive and serious public health concerns associated with numerous detrimental physical and mental health outcomes, including mortality [1–4]. Associations also extend to adverse impacts on prosocial behavior (e.g., volunteering), social participation, healthcare utilization, productivity, and daily functioning [5, 6]. Thus, SIL generates a wide array of harmful and debilitating effects, ranging from individual suffering to broader societal burdens and financial costs [2, 5].

Social isolation and loneliness, although conceptually similar, are distinct and moderately correlated phenomena [7]. Social isolation (“being alone”) refers to an objective state characterized by limited social contacts and infrequent meaningful contact with others [8–10]. In contrast, loneliness (“feeling alone”) is a subjective experience and refers to the negative feeling caused by a discrepancy between actual and desired social connection and social contact [5, 11, 12]. The prevalence of social isolation varies across its specific indicators, but generally increases in later life [13]. For instance, over one-third of adults aged 65 and above, and more than half of those aged over 80, live alone in Norway and several other Western countries [13, 14]. Loneliness is also a widespread issue in Western countries, with approximately one-quarter of the adult population reporting that they “sometimes” or “often” feel lonely [15, 16]. The proportion is even higher among the youngest and oldest age groups and a significant increase among adolescents and young adults has been documented in many Western countries over recent decades [17, 18]. Among older age groups, the rates appear relatively stable, yet the absolute rates will in many countries likely rise in the future due to the aging of the population [19, 20].

Strategies are sought globally to prevent and alleviate SIL [21]. To this end, access to high-quality research evaluating intervention effectiveness is crucial. Although the development and evaluation of SIL interventions are still in their infancy compared to interventions for mental and physical health problems [22], the evidence base is rapidly expanding, accompanied by an increasing number of published systematic reviews (SRs). However, the quality and scope of SRs often vary, in terms of the focal intervention type, populations, delivery format, or outcome, making it challenging to obtain a comprehensive overview of an intervention’s effectiveness [23, 24]. To address this limitation, systematic reviews of systematic reviews (termed umbrella reviews (URs)) can be conducted. URs systematically assess, and synthesize evidence from multiple SRs [25], to offer a comprehensive examination of the available information, allowing for a more robust evaluation of intervention effectiveness [23].

We identified six URs of interventions for reducing SIL [26–31]. These have conflicting findings, making it challenging to draw firm conclusions. Two suggest that interventions have small but significant effects [30], or that specific interventions such as mindfulness, social cognitive training, and social support are effective while others, such as befriending, technological interventions, and social training interventions, are not [31]. The other four URs conclude that interventions generally show no effect [28, 29] or, based on digital interventions, show weak and inconsistent effects [26, 27]. Factors contributing to these diverse findings include different study designs (randomized controlled trials (RCTs) and non-RCTs) and varying quality of evidence and reviews. Half of the URs are not published in peer-reviewed journals. Additionally, the pooling of analyses involving widely different types of interventions and populations further contributes to the challenges in synthesizing the evidence (see Appendix 1 for a detailed description of prior and current umbrella reviews).

Furthermore, these URs reveal several knowledge gaps. Limited research has been conducted on adolescents and younger adults, despite the increasing prevalence of loneliness among these age groups, while half of the URs focus on older adults [26–28]. Only half of the URs also addressed social isolation, which has health impacts similar to loneliness [8]. The evidence also remains scarce for specific types of interventions. For example, the UR supporting the benefits of mindfulness is based on only two RCTs [31]. Non-RCTs and single group (pre-post) designs raise concerns related to internal validity and have been shown to significantly overestimate effect sizes compared to RCTs [32–35]. While concentrating solely on RCT-based evidence limits the scope of intervention types (see Discussion), synthesizing this evidence is imperative for a reliable and accurate evaluation of intervention efficacy [32]. Encouragingly, the number of RCT-evaluated interventions has increased in recent years, and the quality of RCTs appears to be improving [7, 36]. This highlights the need for a further UR to update the evidence and address these limitations.

The aim of this UR is to synthesize and critically appraise scientific evidence on the effectiveness of SIL interventions. This UR includes all types of preventive and mitigating interventions for individuals of all ages. It adds to the existing UR literature by (i) only including SRs of RCTs, (ii) including only the most recent SRs (2017 – 2023), which helps to reduce redundancy and overlap with prior URs, (iii) considering both published and non-published (grey) literature, (iv) analyzing overall and subgroup effects by intervention type, and (v) assessing both social isolation and loneliness. We examine both social isolation and loneliness due to their shared conceptual similarities, significant health impacts, and centrality in public health interventions and strategies. Our review will elucidate whether interventions have similar or distinct effects on these interconnected phenomena. Another aim is to advance insights into interventions designed for youth and young adults, and to investigate the effectiveness of structural interventions, contingent upon the evidence available in recent literature. Our overall objective is to provide updated and valuable insights for researchers, policymakers, and practitioners in this field.

## Methods

This UR was registered with PROSPERO (CRD42022329192) and is reported according to the Preferred Reporting Items for Overview of Reviews (PRIOR) reporting guideline (see Appendix 2) [25]. One deviation from the protocol involved excluding an eligible SR [37] since it included only one relevant RCT, which had already been included in three other included SRs.

### Inclusions and exclusion criteria

Eligible SRs were written in English or Scandinavian languages and published in 2017 or later. SRs were required to have a clear PICO,^1^ a search of two or more databases, and an assessment of risk of bias. Eligible SRs needed to include data from RCTs and provide intervention data (e.g., effect size), with data provided separately for RCTs if non-RCT data were also included. Reviews of any population and any non-pharmacological types of preventive/mitigating intervention (e.g., befriending, social support, psychological interventions) were eligible. Any comparison treatment was acceptable (e.g., treatment-as-usual, other treatment, no care). Reviews including measures of the following outcomes were eligible: loneliness and/or social isolation (or close proxy measures, e.g., social contact). We excluded SRs that did not focus on social isolation or loneliness, SRs that did not measure or report effects comparatively, and SRs using other designs (e.g., scoping reviews).

### Search methods

An information specialist (RAT) conducted a systematic literature search based on a search strategy that combined text words and controlled vocabulary (e.g., MEDLINE Medical Subject Headings), applying a method filter for SRs (see full search strategy in Appendix 3). The strategy was peer-reviewed by a second information specialist. The strategy was adapted for the following databases: MEDLINE (OVID), Embase (OVID), APA PsycINFO (OVID), Sociological Abstracts (ProQuest), CINAHL (EBSCO), Web of Science Core Collection databases (SCI-EXPANDED, SSCI, A&HCI, and ESCI), and Epistemonikos. The search was performed on June 16–17, 2022, and updated on 19 June, 2023. Grey literature was identified through searches in Google Scholar, Swemed + , Prospero, Open Grey System for Information on Grey Literature in Europe, OAIster, and The Campbell Collaboration on June, 20–21, 2022 (updated 22 June 2023). Grey literature is sought to reduce publication bias and enhance the currency of the UR by acknowledging that some relevant reviews may not yet be published or are published as reports, which may not be indexed in journal literature databases. The inclusion criteria and quality assessment methodology were uniformly applied across all studies, whether published or unpublished. Additionally, we contacted researchers with relevant expertise for suggestions of SRs and searched reference lists of included SRs and prior URs (see Appendix 3).

### Screening and selection of reviews

Search results were imported into EndNote [38], where records were de-duplicated. The results were loaded into Covidence [39] for screening. Titles, abstracts, and full-text articles were screened by two review authors independently, with disagreements resolved through discussion. Reasons for excluding full-text SRs were recorded (Appendix 4). When protocols for SRs were identified, up to three emails were sent to the authors requesting copies of the completed SRs. In the absence of a response, the study was excluded from the UR (see Appendix 4).

### Data extraction and quality assessment

Pairs of authors conducted data extraction and quality assessment independently, with disagreements resolved through discussion. An Excel data extraction form was developed and piloted for this project.^2^ For each SR, data on the research question (aim), search strategy (number of databases, grey literature (no/yes), years covered), population, RCT characteristics (number, origin, sample size, sample characteristics (mean age, mental disorder, institutionalized vs. community dwelling, etc.), outcomes and outcome measures, and review characteristics (Cochrane review (no/yes), GRADE assessment (no/yes), risk of bias/quality assessment measure), and results were extracted. We extracted data on intervention characteristics and findings, grouped, if possible, by type of intervention. This included the type of intervention (see below), nature of the intervention (procedure used), delivery format (group vs. individual), comparator(s), mode of delivery (face-to-face, internet, etc.), intervention provider (e.g., therapist, health worker), setting (e.g., long-term care), frequency and duration of intervention, follow-up details, author/year of included primary studies, and findings (e.g., overall and subgroup effects). Inspired by previous categorizations [33, 40, 41], interventions were pre-classified into 11 groups in the data extraction sheet (Table 1).

**Table 1:** Intervention categories (used in the data extraction form)

Social network and contact	Promoting social contact and activity, expanding network size, providing opportunities for social interaction (e.g., online or group-based meeting or activities, video-calls with family, friendship clubs, shared interest groups, day care centers)
Social support	Providing social support through regular contact, care, or companionship (e.g., befriending) typically conducted by a volunteer or peer mentor. Unlike ‘social network and contact’, which focuses on reciprocity and mutual benefit, this category is more one-directional
Psychological/therapy	Addressing social cognition or providing psychological support to cope with distress (e.g., psychotherapy, cognitive-behavioral therapy, mindfulness). The goal is to tackle negative thoughts and beliefs, influence social behavior and self-efficacy, and reduce barriers to socialization and secure social connections. Usually delivered by a trained therapist or health professional
Psychoeducation	Education about topics related to loneliness, health, and well-being more broadly
Social skills training	Educational interventions focused on improving friendship, communication, and interpersonal skills
Computer/internet	Training in the use of information and communication technology, such as internet, email, and social media platforms
Digital	Digitally delivered (e.g., video-conferencing, online support groups). Applied only when used by the systematic reviews and when results from the review’s constituent trials cannot be separated or recategorized based on procedure and content
Physical/exercise	Physical activities, such as walking groups, gardening, or aerobics
Leisure/skill development	Skill development or learning a new hobby
Structural	Organizational (e.g., at workplace or school), community-based (e.g., volunteering), or societal (e.g., policy reform, awareness campaigns). This category refers more to the intervention setting. These interventions are predominantly preventative, aiming to proactively address and mitigate potential issues within these environments before they develop
Mix/Other	"Mix" was used only when applied by the systematic review and when interventions could not be separated or recategorized based on procedure and content. “Other” refers to types of interventions not matching the above categories

These non-mutually exclusive categories center on the interventions’ main objective and action mechanisms. While some interventions may incorporate elements from multiple categories, we presume that each primarily focuses on one area. The classification mainly focuses on content rather than mode of delivery. However, an exception is made for digital interventions. This category is used exclusively when systematic reviews apply it and when results from the constituent trials of the review cannot be distinctly separated or recategorized based on their procedures and content. The first two types can be regarded as “social” (or direct) interventions, as they aim directly at bolstering individuals’ social ties and social connectivity. Social contact/network interventions are designed to enhance social interaction and expand social networks through structured activities or environments that foster interaction among participants. Social support interventions focus on providing support and companionship through befriending and volunteer services. In contrast, the next three are deemed “psychological” (or indirect) interventions, as they target cognitive and behavioral obstacles to social ties, teaching stress management strategies, and improving interpersonal interactions. Therapeutic interventions target social cognition and psychological distress through methods like cognitive-behavioral therapy, focusing on changing negative thoughts and behaviors to improve social behavior and self-efficacy. Psychoeducation provides education on mental health issues, aiming to increase understanding and offer strategies to manage loneliness. Social skills training involves teaching specific skills to enhance communication and interpersonal relationships. The categories of computer/internet and digital interventions are overlapping, as are physical/exercise and leisure/skill development. Structural and mix/other interventions are open categories potentially encompassing a broad spectrum of strategies implemented at various levels.

SRs were critically appraised using the Assessment of Multiple Systematic Reviews (AMSTAR) version 2 [42]. Based on the number of weaknesses in critical domains, we categorized the quality of reviews as moderate (0), low (1), and critically low (2 +) (see Appendix 5). We applied a stringent interpretation of the criteria, and any item that was not fully met, including those rated as 'partial yes', was coded as 'no'.

We assessed the overlap of SRs by comparing the list of included primary studies in each SR. A citation matrix was created with SRs in rows and primary studies in columns to count instances of repeated studies across SRs. This cross-referencing allowed visual inspection and quantification of overlap. We included all overlapping studies, as we were unable to limit inclusion to the highest quality or most comprehensive SR in the case of duplication. However, we report the extent of overlap and consider its possible impact during the interpretation of results.

### Data analysis and synthesis

We grouped the SRs into those with quantitative (meta-analysis) and narrative synthesis analysis. We summarized and synthesized the findings of the two groups separately, and, where possible, according to the type of intervention and outcome. For meta-analyses, we present important parameters (effect size, heterogeneity, number of studies and participants, *p*-values). Effect sizes (including 95% confidence intervals), as reported by the SRs, are the standardized mean differences (SMD) or “Hedges’ g” which adjusts for small sample bias [43]. Unless otherwise stated, all effects refer to favorable (decreasing) changes in loneliness/isolation.^3^ SMD effect estimates ≤ 0.4 are interpreted as low/small effects, 0.40 to 0.70 as moderate, and > 0.7 as large effects [44]. Heterogeneity in pooled estimates is typically summarized using the *I*^*2*^ statistic, which indicates how much of the variance can be attributed to between-study variation. *I*^*2*^ values between 0 and 30% are interpreted as unimportant, 31–60% as moderate, 61–75% as substantial, and 76–100% as considerable [45].

There is no consensus on how to report findings from narrative synthesis [46, 47]. Narrative synthesis provides direction of effect and a counting of significant effects. We have presented numerical data narratively using the metrics, where reported, from the SRs. However, as this information was often unavailable or not synthesized by the review authors (often due to the heterogeneity of the interventions and/or incomplete effect size data), we frequently only had the number of trials with significant effects (“vote count”) to rely on.

In addition to the detailed findings, we provide tables with overall conclusions from the findings of the SRs using the “stop-light model” suggested by Aromataris et al. [48]. We use colors to indicate the overall conclusion regarding evidence of effect based solely on statistical significance. We additionally provide the effect size, number of respondents, and measure of heterogeneity, if available. The color red indicates no evidence of effect (for narrative synthesis; ≥ 75% of the trials show no significant effect), green indicates evidence of effect (for narrative synthesis; ≥ 75% of the trials show significant effect), and orange indicates inconsistent or inconclusive evidence of effect. Evidence based on only 1 RCT is regarded as inconclusive.

## Results

**Fig. 1:**
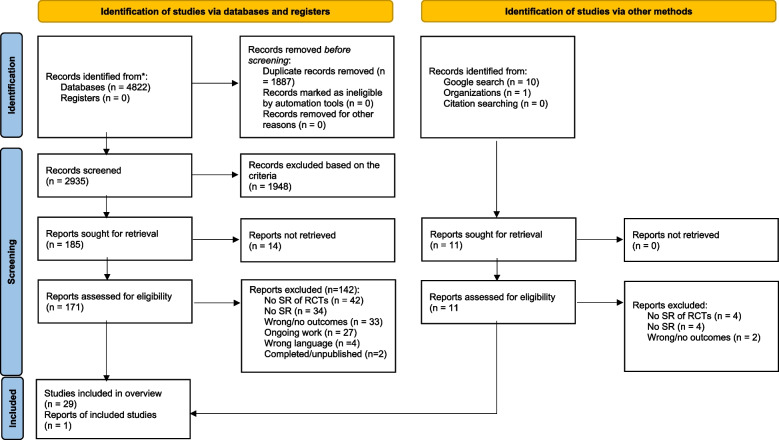
PRISMA flow diagram of the search screening process

Figure 1 summarizes the search results. After removing duplicates, the searches retrieved 2,935 records. Following title and abstract screening, 171 full-text articles were assessed for eligibility based on the inclusion criteria. A total of 29 SRs met our eligibility criteria. Appendix 4 displays the excluded publications and reasons for exclusion, as well as SRs (*n* = 14) identified from review protocols but for which we could not obtain the full text.

### Quality assessment

AMSTAR2 assessments for the included SRs are displayed in supplementary online resources (Appendix 5). SR quality varied, with the number of weaknesses in critical domains ranging from none (4 SRs) to five (1 SR), and the most frequent quality score was one (10 SRs). The most common weaknesses in critical domains were the failure to pre-register a protocol (26/29 SRs), provide a list of excluded studies and reasons for exclusion (25/29 SRs), examine publication bias (12/29 SRs), and discuss the impact of risk of bias on results (7/29 SRs). Consequently, and considering our stringent criteria (coding “partially yes” as “no”), 4 SRs were classified as moderate, 10 as low, and 15 as critically low quality.

The most common weaknesses in non-critical domains were failures to justify the choice of study selection and provide funding information for the included primary studies.

The quality appraisal tool used varied across the reviews, with the Cochrane risk of bias tool emerging as the most frequently employed instrument, featured in 19 SRs (Table 2). There were five larger reviews (≥ 20 RCTs) that used this tool and reported the number of studies rated as having low, moderate, and high risk of bias [7, 10, 36, 49, 50]. On average, 21% of primary studies in the SRs were rated as low, 61% as moderate, and 17% as having a high risk of bias (own calculation). More specifically, the predominant concerns revolved around the procedures for randomization and allocation, the absence of blinding, participant attrition, and selective outcome reporting. The specific nature of the interventions often rendered the blinding of participants or personnel and volunteers unfeasible.

**Table 2:** Characteristics of included systematic reviews

		Search and eligibility criteria				Characteristics of included RCTs			
Study	Objective	Number of data-bases searched	Grey literature sources searched	Years searched	Population details	Number of RCTs	Number of parti-cipants	Outcome	Quality appraisal: tool/rating (number of RCTs)
Abbott 2019 [66]	To determine the effects of robopets on the health and well‐being of older people living in care homes	13	No	Inception-2018	Older people living in care homes/residential care	2	82	Loneliness	NR/Low, High quality
Barnett 2020	To synthesize evidence to improve social circumstances across eight social domains in people with mental health conditions	6	No	2000–2020	Adults aged 18 + with any mental health condition. Only high-income countries	23	2,550	Objective/ subjective isolation	Cochrane RoB Tool/ Low (5), Moderate (13), High (5) quality
Choi 2021 [73]	To investigate the effect of information communication technology interventions on loneliness among the elderly	3	No	2003–2019	Age 60 +	3	370	Loneliness	Cochrane RoB Tool/NR^a^
Christensen 2021 /Lasgaard 2022 [7, 60]	To evaluate and compare the effectiveness of different interventions to reduce loneliness	6	Yes	1980–2020	All ages	54	6,379	Loneliness	Cochrane RoB Tool/: Low (12), Moderate (33), High (9) quality GRADE: Low (≤ 4 weeks)^b^ and Moderate (5–26 weeks)
Eccles 2021 [32]	To examine the effect of interventions to reduce loneliness in young people, and moderators of the effects	4	No	1980-Jan 2020	Age ≤ 25	25	6,750	Loneliness	Tools created by the National Heart, Lung, and Blood Institute and Research Triangle Institute/Poor (11), Fair (7), Good (7)^c^
Ellard 2022	To assess the effectiveness of interventions addressing loneliness in university students	4	Yes	Inception-March 2022	University students	16	NR	Loneliness	Cochrane RoB Tool v.2/NR
Forsman 2018 [71]	To assess the effectiveness of technology-based interventions in promoting the mental health and wellbeing of older adults	7	Yes	2003–2014	Age 65 + or age 55 + and retired	6	752	Loneliness	NICE/Poor (2), Fair (2), Good (2)
Fu 2022 [56]	To evaluate the effects of remotely delivered intervention on loneliness among older adults	5	No	Inception-July 2021	Age 65 +	13	1,045	Loneliness	Cochrane RoB Tool/NR^a^
Gardiner 2018 [41]	To determine the effectiveness of interventions targeting social isolation and loneliness	6	Yes	2003–2016	Age 55 +	6	1,112	Loneliness, social isolation	Hierarchy of evidence (score 3 to 9 (high quality)). Studies with score < 4 excluded/ Scores 7 (1), 8 (1), 9 (4)^d^
Hao 2023 [75]	To understand the effectiveness of telehealth interventions on psychological outcomes in community adults during COVID-19	6	Yes	2019-Oct 2022	Community-dwelling adults aged 18 +	4	495	Loneliness	Cochrane RoB Tool v.2/NR
Heins 2021 [72]	To provide a comprehensive overview of the effects of technological interventions that address social participation in community-dwelling older adults with dementia	5	No	2000- June 2020	Community-dwelling adults aged 55 +	3	170	Loneliness, social interaction	Effective Public Health Practice Project/Moderate to strong quality
Hickin 2021 [49]	To explore the effect of psychological interventions to reduce loneliness across the lifespan, and the moderator of this effectiveness	5	No	2000–2020	Entire population, age range 8–80; Mean 45	31	3,959	Loneliness	Cochrane RoB Tool/ Low (10), Moderate (12), High (9) quality
Hoang 2022 [53]	To evaluate interventions targeting older adults to reduce social isolation and loneliness	7	No	Inception-March 2020	Age 65 +	70	8,259	Social isolation and loneliness	Cochrane RoB Tool/NR GRADE “very low” for each of 10 intervention types
Jin 2021 [76]	To determine the effectiveness of technology-based interventions for reducing loneliness in older adults	7	No	Inception-April 2021	Age 60 +	6	391	Loneliness	Cochrane RoB Tool/ Low (3), Moderate (3) quality
Li 2018 [74]	To synthesize existing studies and provide an overall picture on the social effects of exergames on older adults	4	No	Inception -Jan 2017	Age 55 +	4	282	Loneliness	Cochrane RoB Tool/ Low (2), Moderate or unclear (2) quality
Ma 2020 [59]	To review the evidence for the effectiveness of interventions to improve subjective and/or objective social isolation for people with mental health problems	3	Yes	Inception -July 2017	People with mental health problems	30	3,080	Subjective and objective social isolation	Cochrane RoB Tool/ NR^a^
McElfresh 2021 [57]	To determine the effectiveness of loneliness interventions among adult cancer survivors	7	No	Inception-May 2019	Cancer survivors aged 18 +	7	465	Loneliness	Downs and Black Tool/Low (1), High (2), Very high (4) quality
Osborn 2021 [67]	To assess the acceptability and effectiveness of interventions to reduce and prevent social isolation and loneliness in young people	6	No	NR	Populations that include persons aged 10–25	5	411	Loneliness	Mixed Method Appraisal Tool/NR^a^
Poscia 2018 [58]	To summarize knowledge on the effectiveness of interventions for alleviating social isolation and loneliness among older persons	5	No	2011-Feb 2016	Age 65 +	2	94	Loneliness, social isolation	The Effective Public Health Practice Project Tool/Low quality
Quan 2020 [68]	To review and compare evidence from the past 10 years on the effect of loneliness interventions for older adults living in long-term care facilities	3	No	2009-Jan 2019	Adults aged 65 + living in LTC facilities	5	NR	Loneliness, social isolation	The Quality Assessment of Controlled Intervention Studies/High quality
Shah 2021 [54]	To assess the effectiveness of digital technology interventions in reducing loneliness in older adults	5	No	2010-July 2019	Age 18 +	5	459	Loneliness	Cochrane RoB Tool/ High quality (5) GRADE by month of FU: 3m = moderate, 4m = very low, 6m = moderate
Shvedko 2018 [77]	To examine the physical activity intervention effects on loneliness, social isolation and low social support in community-dwelling older adults	5	Yes	1946–2017	Community-dwelling, healthy/ cognitively intact, older adults aged 60 +	7	NR	Loneliness, social isolation, social network	Cochrane Review Book Group RoB tool/Score 4 to 8 (range 0–12) for the 7 RCTs
Siette 2017 [64]	To evaluate the evidence for the effectiveness of befriending across a range of health conditions and clinical and social outcomes	9	Yes	Inception-2017	All populations	5	1,033	Loneliness	Cochrane RoB Tool/ Low (1), Moderate (1), High (3) quality
Teoh 2021 [55]	To determine the effectiveness and safety of mindfulness-based interventions in alleviating loneliness	5	No	Inception-May 2020	All populations	8	815	Loneliness	Cochrane RoB tool v2/Low (7), Moderate (1) quality GRADE: Low
Tong 2021 [36]	To summarize knowledge on the effectiveness of interventions for alleviating social isolation of older adults	10	No	1978–2021	Adults aged 50 + with no mental illness or cognitive impairment	24	4,078	Loneliness, social isolation	Cochrane RoB tool/ Moderate (17), High (7) quality
Williams 2021 [61]	To identify and assess the effectiveness of interventions to reduce social isolation and loneliness that are compatible with COVID-19 shielding and social distancing measures	6	Yes	Inception-April 2020	Non-hospitalized persons of any age	45	NR	Loneliness, social isolation	Downs and Black Tool/NR^a,e^
Wiwatkunu-pakarn 2021	To examine the relationship between social network site usage and social isolation, loneliness, and depression among older adults	3	No	Inception-2020	Age 60 +	4	551	Loneliness, social isolation	Cochrane RoB Tool/ NR^a^
Zagic 2021 [10]	To determine the effect of interventions designed to promote ‘objective social contact’ and the ‘quality of social connections’	4	No	1980–2020	Age 18 +	58	8,780	Objective social contact, perceived quality of social connection	Cochrane RoB Tool v2/Low (6), Moderate (45), High (7) quality^f^
Zhang 2023 [65]	To determine the effectiveness of psychological and exercise interventions compared with no treatment for problematic mobile phone use in RCTs	10	Yes	Inception-Aug 2022	Chinese middle-school/university students with problematic mobile phone use	8	1,107	Loneliness	Cochrane RoB Tool/ NR

*RoB* Risk of bias, *NR* not reported, *FU* Follow-up

^a^Detailed (but no overall) ratings provided in the paper

^b^GRADE = Moderate in all subgroup analyses by type of intervention

^c^Reported per type of intervention (# Poor-Fair-Good): Support (0–4-0), social skills (2–0-3), social and emotional skills (5–1-1), psychological (2–3-3), learning hobby (2–0-0)

^d^Psychological (all: High (score 9 of 9)), animal-assisted (Score 7/8 out of 9)

^e^Reported per type of intervention (# Poor-Fair-Good). Social facilitation (3–2-5), Support (1–2-0), psychological (0–5-5), psychoeducation (0–3-1), Animal-assisted (1–2-0), Health/social care (0–1-1), Leisure/skill development (14–2-1)

^f^Reported per type of intervention (# Poor-Fair-Good). Social access (1–11-3), Support (3–11-1), Social skills (0–2-0), psychological (0–8-4)

### Description of included reviews

Key characteristics of the 29 included SRs are presented in Table 2. The SRs were published from 2017, with the majority published since 2020 (22 SRs). Among the 29 SRs, 28 reported search dates, with the most recent searches being conducted up until 2020 (9 SRs), 2021 (2 SRs), and 2022 (4 SRs). The number of databases searched ranged from 3 to 10, with 5 − 7 databases being the most common (16 SRs). Grey literature was included in 10 SRs.

Approximately equal numbers of SRs focused on all types of interventions (15 SRs) and specific types of interventions (14 SRs). Some reviews included all population groups (5 SRs), while others specifically targeted the young (4 SRs), adults (3 SRs), older adults (14 SRs), people with mental health problems (2 SRs), or cancer survivors (1 SR). The number of RCTs included in the reviews ranged from 2 to 58 (< 10 in 18 SRs). The total number of participants ranged from 82 to 8,780 (> 1,000 in 12 SRs, not reported in three SRs). All SRs focused on loneliness, with 12 SRs additionally focusing on social isolation.

As Table 3 illustrates, the most frequently investigated interventions were psychological, digital, social contact, social support, and social skills training (all with ≥ 8 SRs). Eight SRs encompassed a mix of various intervention types (for meta-analysis; pooled analysis across intervention types).

**Table 3:** Number of included systematic reviews with meta-analysis or narrative synthesis by type of intervention

Type of intervention	Number of systematic reviews per intervention type		
	Meta-analysis	Narrative analysis	Total
Psychological/therapy	10	9	19
Digital (incl. computer/internet)	5	4	9
Diverse types	5	3	8
Social contact/network	3	5	8
Social support	6	2	8
Social skills training	3	4	7
Other^a^	5	2	7
Psychoeducation	2	4	6
Leisure/skill development	1	1	2
Physical/exercise	1	—	1
Structural	—	—	—
Total^b^	41	34	75

^a^Multicomponent intervention (5 reviews), music, health and social care

^b^The total exceeds the number of included SRs, as each SR may cover multiple intervention types

The assessment of overlap in primary studies across the included SRs is detailed in Appendix 6. Of 256 primary studies in the SRs, 163 (64%) were “unique” and reviewed by only one SR, 49 (19%) by two SRs, and 45 (18%) by at least three SRs.

Some additional features of the SRs, not shown in the tables, deserve mention. Typically, loneliness was measured using the UCLA Loneliness scale [51], the De Jong Gierveld Loneliness Scale [52], or single item measures. Social isolation was gauged by different measures, and the most frequently used was the Lubben Social Network Scale [53]. None of the SRs were Cochrane reviews. Further details about each type of intervention are discussed below.

### Summary of results

Detailed results for each type of intervention are provided in Tables 4, 5, 6, 7, 8, 9, 10, as well as in summarized Tables 11, 12, 13, 14, 15, 16, 17 using the stop-light model. The effects of interventions were quantified as SMD or Hedges’ g with 95% confidence intervals in 13 meta-analyses and were subject to narrative synthesis in terms of significance testing and sometimes mean differences in 13 SRs. Overall, the extent to which the SRs provided details about populations, comparators, delivery (individual vs. group), mode (face-to-face, internet, etc.), frequency and duration, and follow-up measurement varied greatly. An overall summary of the certainty of the evidence (GRADE) was reported by only four SRs [40, 53–55]. A review of 54 RCTs focusing on loneliness for diverse populations reported a low GRADE rating for the overall (pooled) evidence and a moderate rating for RCTs within each of five intervention types [40]. Another review of 70 RCTs targeting older adults reported a “very low” rating for each of ten intervention types focusing on social isolation and loneliness [53]. In general, there was no evidence or reporting that interventions did any harm.

**Table 4:** Characteristics of systematic reviews of diverse types (≥ 2 types) of interventions

Author year Outcome Population** details**	Intervention details (number of studies)		Findings Meta-analysis: Effect sizes (95% CI), subgroup analysis Narrative synthesis: Report of significant effects
	Intervention vs. comparator	Delivery: group vs. individual Mode: F2F, internet, etc Frequency/duration (F/D) Follow-up (FU)	
Reviews with meta-analysis			
Christensen 2021/Lasgaard 2022 [7, 60] Loneliness Diverse^a^	Social support (19), social network (16), social and emotional skills training (26), psychological treatment (17), psychoeducation (6) vs. NR	Details provided separate by type of intervention, see Tables 5, 6, 7, 8, 9, 10	Short-term effect (≤ 4 weeks): SMD -0.47 (-0.61; -0.33), *I*^*2*^ = 83%, 54 studies (*n* = 6,379) Long-term (5–26 weeks) effect: SMD -0.49 (-0.76, -0.23), *I*^*2*^ = 85%, 18 studies (*n* = 1,826) Based on short-term effects: **- age** 6–25 (SMD -0.30 (-0,47; -0.13), 14 studies), age 26–64 (SMD -0.29 (-0.48; -0.10), 12 studies), and age 65 + (SMD -0.60 (-0.88; -0.33), 28 studies) - **group based** (SMD -0.53 (-0.72; -0.34), 37 studies) vs. individual (SMD -0.31 (-0.49; -0.15), 16 studies) - **digital** (SMD -0.38 (-0.61; -0.19), 14 studies) vs. non-digital (SMD -0.49 (-0.67; -0.32), 40 studies) **- study quality**: high (SMD -0.43 (-0.79; -0.08), 9 studies, *I*^*2*^ = 81%), moderate (SMD -0.53 (-0.75; -0.30), 32 studies, *I*^*2*^ = 87%), low (SMD -0.40 (-0.56; -0.23), 13 studies, *I*^*2*^ = 59%)
Eccles 2021 [32] Loneliness Age ≤ 25	Social support (4), social skills (5), social and emotional skills (7), psychological (8), learning new skills (2) vs. NR	Details provided separate by type of intervention, see Tables 5, 6, 7, 8, 9, 10	g 0.32 (0.19; 0.44), *I*^*2*^ = 67%, 25 studies (*n* = 6,750) - **study quality**: poor g = 0.42 (0.22; 0.63), 11 studies; fair g = 0.26 (0.06; 0.45), 7 studies; good g = 0.26 (0.04; 0.48), 7 studies
Fu 2022 [56] Loneliness Diverse^a^	Social network (6), social support (3), social skills (1), social cognition (3) vs. TAU (5), brief contact (2), no treatment (4), social activity (2)	Details provided separate by type of intervention, see Tables 5, 6, 7, 8, 9, 10	SMD -0.41 (-0.70; -0.13), *I*^*2*^ = 79%, 13 studies (*n* = 1,045) - **individually delivered** interventions SMD -0.39 (-0.71; -0.07), *I*^*2*^ > 50%, 6 studies vs. **group** (5 studies) and **mixed** format (2 studies) both p > .05 (ES not reported) - **time of follow-up**: evidence of effect found < 3 months SMD -0.33 (-0.52; -0.14), *I*^*2*^ < 50%; 3–6 months SMD -0.32 (-0.57; -0.07), I^2^ > 50%; but not > 6 months SMD 0.37 (-0.02; 0.76), *I*^*2*^ NR
McElfresh 2021 [57] Loneliness Cancer survivors	Social support (4), social access (1), social cognitive training (1), social skills training (1) vs. NR	Delivery: Group (3), indiv. (4) Mode: Mostly F2F F/D: NR/6-13m FU: NR	g -0.32 (-0.50; -0.14), *I*^*2*^ = 17%, 7 studies (*n* = 465)
Zagic 2021 [10] Social isolation, loneliness Diverse^a^	Social support (4), social access (1), social cognitive training (1), social skills training (1) vs. NR	Details provided separate by type of intervention, see Tables 5, 6, 7, 8, 9, 10	Social isolation (significant only after removing one outlier): g 0.43 (0.21, 0.65), *I*^*2*^ = 46%, 10 studies (*n* = NR) Loneliness: g -0.33 (-0.51; -0.16), *I*^*2*^ = 77%, 32 studies (*n* = NR)
Reviews with narrative synthesis			
Ma 2020 [59] Social isolation, loneliness Mental health problems	Supported socialization (3), social skills and psychoeducation (6), psychological (4), other (12) vs. TAU, no/other treatment	Delivery: NR Mode: NR F/D: NR FU: NR	Social isolation: 3/8 trials showed an effect. 5/8 showed no evidence of effect Loneliness: No evidence of effect in any of the 6 trials
Poscia 2018 [58] Loneliness Age 65 +	Social support (1), animal therapy (1) vs. No treatment, other activity	Delivery: NR Mode: F2F F/D: NR FU: 6m, 12m	Both trials showed evidence of effect
Tong 2021 [36] Social isolation, loneliness Age 50 +	Group interventions (8), individual interventions (6), mixed (4). Content details NR vs. No intervention, other therapy, telephone calls, waiting list, community service, other	Delivery: Mix Mode: F2F, internet F/D: Weekly/6-12m FU: NR	Social isolation: 12/19 trials showed evidence of effect Loneliness: 9/19 trials showed evidence of effect

*F2F* Face-to-face, *TAU* treatment as usual, *W* weeks, *M* months, *Y* years, *ES* Effect size, *N* number of participants, *g* Hedges’ g, *SMD* standardized mean difference, *NR* not reported, *RCT* Randomized controlled trial, *SI* Social isolation, *L* Loneliness, *LTC* Long-term care

^a^Not limited to a specific group. *Effect* indicates a significant (*p* < .05) effect in favor of the intervention

**Table 5:** Characteristics of the systematic reviews of social network/contact interventions

Author year Outcome Population details	Intervention details (number of studies)		Findings Meta-analysis: Effect sizes (95% CI), subgroup analysis Narrative synthesis: Report of significant effects
	Intervention vs. comparator	Delivery: group vs. individual Mode: F2F, internet, etc Frequency/duration (F/D) Follow-up (FU)	
Reviews with meta-analysis			
Christensen 2021 Loneliness Diverse^a^	Social network (e.g., senior meetings, physical activity groups, choir, arts) vs. NR	Delivery: Mix Mode: NR F/D: NR FU: NR	SMD -0.30 (-0.50; -0.09), *I*^*2*^ > 65%, 15 studies (*n* = NR)
Fu 2022 [56] Loneliness Age 65 +	Remotely delivered (e.g., phone, video-call, internet) vs. No treatment (4), TAU (1), social activity (1)	Delivery: About 50–50 Mode: Phone, internet F/D: NR/2-30w Follow-up: NR	SMD -0.13 (-0.55; 0.29), *I*^*2*^ = 76%, 6 studies (*n* = 411)
Zagic 2021 [10] Social isolation, loneliness Diverse^a^	Social access (details NR) vs. TAU, other activity	Delivery: NR Mode: NR F/D: Weekly/26-52w FU: NR	Social isolation: g 0.67 (0.36; 0.98), *I*^*2*^ = 17%, 4 studies (*n* = NR) Loneliness: g -0.13 (-0.41; 0.17), *I*^*2*^ = 60%, 8 studies (*n* = NR)
Reviews with narrative synthesis			
Barnett 2020 Social isolation, loneliness Mental health problems	Supported socialization (e.g., watching films with others, social network intervention, activities with volunteer, self-help training course) vs. TAU	Delivery: NR Mode: NR F/D: NR FU: NR	Social isolation: 3/4 trials showed evidence of effect Loneliness: 1/8 trials showed evidence of effect (n’s = NR)
Ellard 2022 Loneliness University students	Social interaction (shared activities, e.g., sports, creative exercises) vs. NR	Delivery: Group Mode: NR F/D: NR FU: NR	3/4 trials showed evidence of effect (*n* = 13–190)
Ma 2020 [59] Social isolation, loneliness Mental health problems	Supported socialization (details NR) vs TAU, no treatment, other treatment	Delivery: NR Mode: NR F/D: NR/12 weeks-2y FU: 2y (1)	Social isolation: 2/2 trials showed evidence of effect. One trial found evidence of effect also after 2 years Loneliness: 1/3 trials showed evidence of effect
Williams 2021 [61] Social isolation, loneliness NR	Compatible with COVID-19 social distancing (e.g., computer/internet training, videoconferencing, online group meetings) vs. TAU, other activity	Delivery: NR Mode: digital F/D: Weekly/6-12m FU: NR	Social isolation: 1 poor-quality RCT showed evidence of effect, 2 good-quality RCTs showed no evidence of effect Loneliness: 4 RCTs (1 fair, 3 good quality) showed evidence of effect. Two of these were videoconferencing for nursing home residents. 2 poor-quality RCTs showed no evidence of effect
Wiwatkunupakarn2021 Loneliness Age 60 +	Social network site usage (e.g., internet training, social network site use) vs. TAU	Delivery: NR Mode: Internet F/D: NR FU: NR	1/4 trials showed evidence of effect (*n* = 551)

*F2F* Face-to-face, *TAU* treatment as usual, *W* weeks, *M* months, *Y* years, *ES* Effect size, *N* number of participants, *g* Hedges’ g, *SMD* standardized mean difference, *NR* not reported, *RCT* Randomized controlled trial, *SI* Social isolation, *L* Loneliness, *LTC* Long-term care

^a^Not limited to a specific group. *Effect* indicates a significant (*p* < .05) effect in favor of the intervention

**Table 6:** Characteristics of the systematic reviews of social support interventions

Author year Outcome Population details	Intervention details (number of studies)		Findings Meta-analysis: Effect sizes (95% CI), subgroup analysis Narrative synthesis: Report of significant effects
	Intervention vs. comparator	Delivery: group vs. individual Mode: F2F, internet, etc Frequency/duration (F/D) Follow-up (FU)	
Review with meta-analysis			
Christensen 2021 Loneliness NR	Enhancing social support (e.g., home visiting schemes, befriending services and mentorship programs) vs. TAU	Delivery: Mix Mode: NR F/D: NR FU: NR	SMD -0.39 (-0.56; -0.23), *I*^*2*^ > 65%, 22 studies (*n* = NR)
Eccles 2021 [32] Loneliness Age 13–19 (students 2, ASD 1, NR 1)	Enhancing social support (examples NR) vs. NR	Delivery: Mix Mode: F2F F/D: NR/3-7m FU: NR	g 0.21 (-0.16; 0.59), 4 studies (*n* = 1,294). *I*^*2*^ NR (only one of the constituent RCTs found evidence of effect)
Fu 2022 [56] Loneliness Older adults (isolated elderly 2, caregivers 1)	Social support—remotely delivered (via telephone) vs. TAU, brief contact, no treatment	Delivery: Group (2), indiv. (1) Mode: Telephone F/D: 1–5 times per w/4–8 weeks FU: 24 weeks (2), no (1)	SMD -0.47 (-0.77; -0.18), *I*^*2*^ = 42%, 3 studies (*n* = 388)
Hoang 2022 [53] Loneliness Age 65 + , community setting	Social intervention (befriending, support groups) vs. TAU (3), no intervention (4), other activity (1)	Delivery: Mix Mode: F2F (5), telephone (3) F/D: NR/6w-2y FU: No	SMD -0.02 (-0.21; 0.17), *I*^*2*^ = 7%, 5 studies (*n* = NR)
Siette 2017 [64] Loneliness Caregiver 1, isolated elderly 2, physical or mental health problems 2	Befriending (one-to-one companionship provided regularly by a volunteer) vs. TAU, no treatment	Delivery: Individual Mode: F2F, telephone F/D: 1–2 per week/6w-12m FU: 2-9m (3)	SMD -0.03 (-0.18; 0.12), *I*^*2*^ = 0%, 5 studies (none of the trials showed evidence of short-term or long-term effects) (*n* = NR)
Zagic 2021 [10] Social isolation, loneliness NR	Social support (regular contact, care, or companionship) vs. TAU, other activity	Delivery: NR Mode: NR F/D: Weekly/6-12m FU: NR	Objective social contact: g 0.29 (-0.09; 0.67), *I*^*2*^ = 49%, 4 studies Perceived social isolation: g -0.24 (-0.61; 0.14), *I*^*2*^ = 87%, 10 studies (n’s = NR)
Reviews with narrative synthesis			
Ellard 2022 Loneliness University students	Social support (group-based interventions to build friendships) vs. NR	Delivery: Group Mode: NR F/D: NR FU: NR	1/1 RCT showed evidence of effect (*n* = 171)
Williams 2021 [61] Social isolation, loneliness NR	Befriending compatible with COVID-19 physical distancing measures (telephone calls/home visits) vs. NR	Delivery: NR Mode: Digital F/D: NR FU: NR	Social isolation: 1/1 trial showed no evidence of effect (*n* = 291) Loneliness: 1/2 trials showed evidence of effect (*n* = 331)

*F2F* Face-to-face, *TAU* treatment as usual, *W* weeks, *M* months, *Y* years, *ES* Effect size, *N* number of participants, *g* Hedges’ g, *SMD* standardized mean difference, *NR* not reported, *RCT* Randomized controlled trial, *SI* Social isolation, *L* Loneliness, *LTC* Long-term care

^1^Not limited to a specific group. *Effect* indicates a significant (*p* < .05) effect in favor of the intervention

**Table 7:** Characteristics of the reviews on psychological interventions

Author year Outcome Population details	Intervention details (number of studies)		Findings Meta-analysis: Effect sizes (95% CI), subgroup analysis Narrative synthesis: Report of significant effects
	Intervention vs. comparator	Delivery: group vs. individual Mode: F2F, internet, etc Frequency/duration (F/D) Follow-up (FU)	
Reviews with meta-analysis			
Abbott 2019 [66] Loneliness Older adults in LTC	Robopets (spending time with robotic animals) vs. No intervention, normal dog	Delivery: Mix Mode: F2F F/D: Weekly or biweekly/8–12 w FU: No	SMD -0.51 (-1.24; 0.22), *I*^*2*^ = 46% (1/2 trials showed evidence of effect), 2 studies (*n* = 59)
Christensen 2021 Loneliness Diverse^a^	Psychological (examples NR) vs. NR	Delivery: Mix Mode: NR F/D: NR FU: NR	SMD -0.50 (-0.74; -0.26), *I*^*2*^ > 65%, 16 studies (*n* = NR)
Eccles 2021 [32] Loneliness Age 10–25. 5/8 studies: at-risk (war-affected, depressive symptoms, lonely, incarcerated, substance abuse)	Psychological (examples NR) vs. NR	Delivery: Group (7), ind (1) Mode: F2F F/D: Mostly weekly/5–12 w FU: 3–6 m (4)	g 0.36 (0.12; 0.60), *I*^*2*^ NR (4/8 trials found evidence of effect), 8 studies (*n* = NR)
Fu 2022 [56] Loneliness LTC (2), isolated (1)	Addressing maladaptive social cognition, remotely delivered (examples NR) vs. TAU (1), other activity (1)	Delivery: Group (2), ind (1) Mode: Internet, telephone, video call F/D: NR/4–7 weeks FU: NR	SMD -1.04 (-1.98; -0.10), *I*^*2*^ = 87%, 3 studies (*n* = 178)
Hickin 2021 [49] Loneliness Age 8–81 (M = 45). Children (4), Age 18–25 (1), Age 65–74 (10), 75 + (4)	Psychological (CBT 9, mindfulness 3, integrative 6, interpersonal therapy 1, reminiscence therapy 1, social skills training 3, social identity 1, gratitude 1) vs. Waitlist (14), other activity (11), no treatment (6)	Delivery: Group 16, ind 8, mix 7 Mode: F2F (24), phone or internet (7) F/D: Mostly weekly/1-52w (mean 10w) FU: NR	SMD 0.43 (0.18; 0.68), *I*^*2*^ = 90%, 31 studies (*n* = 3,959) Moderation analysis of effect of different types of interventions: *p* = .06. Reminiscence, social identity, and CBT had the highest effect sizes
Hoang 2022 [53] Social isolation, loneliness Age 65 + , community setting	CBT and psychotherapy vs. TAU (3), other activity (2)	Delivery: Mix Mode: F2F F/D: NR/5w-3m FU: No	Social isolation: SMD 0.16 (-0.06; 0.38), 1 study Loneliness: SMD -0.52 (-1.21; 0.17), *I*^*2*^ = 83%, 4 studies (n’s = NR)
Hoang 2022 [53] Loneliness Age 65 +	Animal-assisted therapy (living dog, robotic animal) vs. TAU (4), other activity (1)	Delivery: Mix Mode: F2F F/D: NR/6-12w FU: NR	SMD -1.86 (-3.14; -0.59), *I*^*2*^ = 86%, 5 studies (after excluding one study comparing group to individual animal therapy) (*n* = NR)
Teoh 2021 [55] Loneliness Students (2) and adults (6); with (3) or without (3) mental health problems; lonely (1)	Mindfulness (mindfulness stress-reduction/CBT, cognitively based compassion training, meditation, yoga) vs. Wait list, other activity (e.g. health education class, guidance in free reflection (not mindfulness), aerobic, no treatment	Delivery: Group Mode: F2F (7), phone (1) F/D: Mostly weekly/8w-2y FU: NR	- Mentally healthy (control = waitlist) showed evidence of effect: MD -6.33 (-9.39; -3.26), *I*^*2*^ = 0%, 3 studies; GRADE low - Mentally unhealthy: no evidence of effects: SMD -0.23 (-0.80; 0.33), *I*^*2*^ = 63%, 3 studies; GRADE very low) - Age 17–30: SMD -0.85 (-1.36; -0.35), *I*^*2*^ = 0, 2 studies; GRADE low - Age > 30: SMD -0.12 (-0.43; 0.19), *I*^*2*^ = 18%,5 studies: GRADE low Overall, 4/8 trials (*n* = 815) showed evidence of effect
Zagic 2021 [10] Loneliness NR	Psychological (e.g., psychotherapy, CBT, mindfulness) vs. NR	Delivery: Mix Mode: Mix tech/nontech F/D: From daily to weekly/1d-39w FU: NR	g -0.53 (-0.79; -0.26), *I*^*2*^ = 71%, 12 studies (*n* = NR)
Reviews with narrative synthesis			
Barnett 2020 Social isolation, loneliness Mental health problems	Changing cognitions (reframing, social cognition and interaction training, social mentoring, CBT) vs. Waitlist (3), other activity (3), no intervention (1), unknown (1)	Delivery: Group (7), smartphone (1) Mode: F2F (7), phone (1) F/D: 1–2 times per w/8w (7) or 2y (1) FU: NR	Social isolation: 0/4 trials showed evidence of effect Loneliness:1/2 trials showed evidence of effect (n’s NR)
Ellard 2022 Loneliness University students	Reflexive exercises (e.g., mindfulness/meditation, journal writing) vs. NR	Delivery: Group Mode: NR F/D: NR FU: NR	6/7 trials showed evidence of effect (*n* = 33–139)
Gardiner 2018 [41] Loneliness Age 55 + , home-dwelling	Psychological therapies (mindfulness, stress-reduction, rehabilitation, support, cognitions) vs. NR	Delivery: Group Mode: F2F F/D: 0,5–1 times per w/8-12w FU: NR	2/3 trials showed evidence of effect (*n* = 330)
Gardiner 2018 [41] Loneliness Older adults in LTC	Animal assisted therapy (visit from living/robotic dog) vs. TAU	Delivery: Group Mode: F2F F/D: Weekly/6-8w FU: NR	2/2 trials showed evidence of effect (*n* = 75)
Ma 2020 [59] Social isolation, loneliness Mental health problems	Changing cognitions (examples NR) vs. TAU, no/other treatment	Delivery: NR Mode: NR F/D: NR FU: NR	Social isolation: 1/2 trials showed evidence of effect Loneliness: 2/6 trials showed evidence of effect (n’s = NR)
Osborn 2021 [67] Loneliness Age 14–25 “at risk of loneliness”	Psychological (CBT and positive psychology-oriented interventions to address cognitions, self-compassion, and competence) vs. NR	Delivery: Individual Mode: Internet (1), smartphone (1) F/D: NR FU: NR	2/3 trials showed evidence of effect (*n* = 361)
Quan 2020 [68] Loneliness Older adults living in LTC	Therapy (reminiscence 2, pet 2) vs. TAU, other activity, waitlist	Delivery: Individual Mode: F2F F/D: 1–2 sessions weekly/8-12w FU: NR	4/4 trials showed evidence of effect (*n* = NR)
Williams 2021 [61] Social isolation, loneliness NR	Psychological therapy compatible with COVID-19 social distancing (e.g., mindfulness, CBT, other therapy) vs. NR	Delivery: NR Mode: NR F/D: NR FU: NR	Social isolation: 2/2 trials showed evidence of effect (fair-quality logotherapy, good-quality Tai Chi) Loneliness: 4/7 showed evidence of effect. Effects found for 2 good-quality mindfulness, 2 fair-quality reminiscence/CBT, and 1 good-quality Tai Chi. No effect: 1 fair-quality reminiscence therapy, 2 fair/good quality CBT
Williams 2021 [61] Loneliness NR	Animal intervention compatible with COVID-19 social distancing (real or artificial animals: animal-assisted therapy, companionship) vs. NR	Delivery: NR Mode: NR F/D: NR FU: NR	2/3 trials showed evidence of effects (*n* = 118). Evidence of effect found in 2 poor/fair quality RCTs. No evidence of effect found in 1 fair quality RCT

*F2F* Face-to-face, *TAU* treatment as usual, *W* weeks, *M* months, *Y* years, *ES* Effect size, *N* number of participants, *g* Hedges’ g, *SMD* standardized mean difference, *NR* not reported, *RCT* Randomized controlled trial, *SI* Social isolation, *L* Loneliness, *LTC* Long-term care

^a^Not limited to a specific group. *Effect* indicates a significant (*p* < .05) effect in favor of the intervention

**Table 8:** Characteristics of the reviews on psychoeducation interventions

Author year Outcome Population detail**s**	Intervention details (number of studies)		Findings Meta-analysis: Effect sizes (95% CI), subgroup analysis Narrative synthesis: Report of significant effect**s**
	Intervention vs. comparator	Delivery: group vs. individual Mode: F2F, internet, etc Frequency/duration (F/D) Follow-up (FU)	
Reviews with meta-analysis			
Christensen 2021 Loneliness NR	Psychoeducation (examples NR) vs. NR	Delivery: Mix Mode: NR F/D: NR FU: NR	SMD = -1.12 (-2.61; 0.36), *I*^*2*^ > 65%, 4 studies (*n* = NR)
Hoang 2022 [53] Loneliness Age 65 +	Counseling (bereavement counseling, instructor-led educational programs) vs. TAU (5), other activity (1), NR (1)	Delivery: Group Mode: F2F F/D: NR/2-8w FU: No	SMD -0.19 (-0.35; -0.03), *I*^*2*^ = 0%, 5 studies (after excluding one study) (*n* = NR)
Reviews with narrative synthesis			
Barnett 2020 Loneliness Mental health problems	Psychoeducation (e.g., education, guided peer support, social identity) vs. TAU	Delivery: NR Mode: F2F F/D: NR FU: NR	1/4 trials showed evidence of effect (*n* = 434)
Ellard 2022 Loneliness University students	Psychoeducation (cognitive restructuring exercises, social skills training, e.g., through roleplaying or gamification) vs. NR	Delivery: Group Mode: F2F, internet F/D: NR FU: NR	9/14 trials showed evidence of effect (*n* = 1,412)
Ma 2020 [59] Social isolation, loneliness Mental health problems	Social skills training and/or psychoeducation (examples NR) vs. TAU, no/other treatment	Delivery: NR Mode: NR F/D: NR FU: NR	Social isolation: 1/2 trials showed evidence of effect Loneliness: 1/4 trials showed evidence of effect (n’s NR)
Williams 2021 [61] Loneliness NR	Educational program compatible with COVID-19 social distancing (topics relevant to social isolation/loneliness or health/well-being) vs. NR	Delivery: NR Mode: NR F/D: NR FU: NR	Evidence of effect found for 2 fair-quality RCTs on friendship and social integration education (*n* = 313) No evidence of effect found for 2 fair/good quality RCTs (*n* = 430)

*F2F* Face-to-face, *TAU* treatment as usual, *W* weeks, *M* months, *Y* years, *ES* Effect size, *N* number of participants, *g* Hedges’ g, *SMD* standardized mean difference, *NR* not reported, *RCT* Randomized controlled trial, *SI* Social isolation, *L* Loneliness, *LTC* Long-term care

^1^Not limited to a specific group. *Effect* indicates a significant (*p* < .05) effect in favor of the intervention

**Table 9:** Characteristics of the systematic reviews of social skills interventions

Author year Outcome Population details	Intervention details (number of studies)		Findings Meta-analysis: Effect sizes (95% CI), subgroup analysis Narrative synthesis: Report of significant effects
	Intervention vs. comparator	Delivery: group vs. individual Mode: F2F, internet, etc Frequency/duration (F/D) Follow-up (FU)	
Reviews with meta-analysis			
Christensen 2021 Loneliness Diverse^a^	Social and emotional skills training (e.g., role-play, conversation-based training) vs. NR	Delivery: Mix Mode: NR F/D: NR FU: NR	SMD -0.38 (-0.62; -0.15), *I*^*2*^ > 65%, 21 studies (*n* = NR)
Eccles 2021 [32] Loneliness At-risk clinical (social phobia 2, cystic fibrosis 1, ASD 2)	Social skills training (examples NR) vs. NR	Delivery: Group Mode: F2F F/D: 1–2 sessions per w/12–14 w FU: 6-9m (2)	g 0.44 (0.10; 0.79), *I*^*2*^ NR (2/5 trials found evidence of effect), 5 studies (*n* = NR)
Eccles 2021 [32] Loneliness Age 3–15 (general 2, at-risk 5: developmental disorder, problem behavior)	Social and emotional skills (examples NR) vs. NR	Delivery: Group (4), Ind (3) Mode: Tech (3), Non-tech (4) F/D: Weekly/6-12m FU: 3–6 m (3)	g 0.27 (-0.01; 0.53), 7 studies. *I*^*2*^ NR (3/7 trials found evidence of effect)
Reviews with narrative synthesis			
Barnett 2020 Social isolation Mental health problems	Supported socialization (examples NR) vs. Skill training, other therapy	Delivery: NR Mode: NR F/D: NR FU: NR	3/3 trials showed evidence of effect
Ma 2020 [59] Social isolation, loneliness Mental health problems	Social skills training and/or psychoeducation (examples NR) vs. TAU, no/other treatment	Delivery: NR Mode: NR F/D: NR FU: NR	Social isolation: 1/2 trials showed evidence of effect Loneliness: 1/4 trials showed evidence of effect (n’s NR)
Osborn 2021 [67] Loneliness Age 13–23 and ASD	Social skills and function (PEERS program) vs. NR	Delivery: Group Mode: F2F F/D: Weekly/8w FU: NR	2/2 trials showed evidence of effect (*n* = 56)
Zagic 2021 [10] Loneliness Mean age 20 (1), 63 (1)	Social skills training (interpersonal communication skills) vs. NR	Delivery: Group Mode: F2F F/D: Weekly/6-8w FU: NR	1/2 trials showed evidence of effect. One trial showed evidence of effect (g -1.04 (-2.01; -0.07), *n* = 17) among young people with ASD. One trial showed no evidence of effect among older women (*n* = 142)

*F2F* Face-to-face, *TAU* treatment as usual, *W* weeks, *M* months, *Y* years, *ES* Effect size, *N* number of participants, *g* Hedges’ g, *SMD* standardized mean difference, *NR* not reported, *RCT* Randomized controlled trial, *SI* Social isolation, *L* Loneliness, *LTC* Long-term care

^a^Not limited to a specific group. *Effect* indicates a significant (*p* < .05) effect in favor of the intervention

**Table 10:** Characteristics of the reviews on digital interventions

Author year Outcome Population details	Intervention details (number of studies)		Findings Meta-analysis: Effect sizes (95% CI), subgroup analysis Narrative synthesis: Report of significant effects
	Intervention vs. comparator	Delivery: group vs. individual Mode: F2F, internet, etc Frequency/duration (F/D) Follow-up (FU)	
Reviews with meta-analysis			
Hao 2023 [75]	Telehealth (videoconferencing, telephone/internet-based psychoeducation or counseling/therapy) vs. NR	Delivery: NR Mode: Internet F/D: NR FU: NR	SMD -0.63 (-1.44; 0.18), *I*^*2*^ = 94% (*n* = NR)
Hoang 2022 [53] Social isolation, loneliness Age 65 +	Technology (e.g., computer-training, videoconferencing, internet-based exercise) vs. TAU (3), other activity (6), waitlist (1), no intervention (4), NR (1)	Delivery: NR Mode: Internet F/D: NR/6w-1y FU: No	Social isolation (community setting): SMD -0.18 (-0.43; 0.08), 1 study Loneliness (community setting): SMD -0.19 (-0.51; 0.14), *I*^*2*^ = 59%, 7 studies Loneliness (LTC): SMD -1.40 (-2.37; -0.44), *I*^*2*^ = 70%, 2 studies. (n’s = NR)
Jin 2021 [76] Loneliness Age 60 +	Technology-based (digital smartphone-based videoconferencing to interact with family members (3), computer training/internet use (2), teleconferences (1) vs. Regular care, regular family visits, alternative activities	Delivery: NR Mode: Internet F/D: Weekly or biweekly/1-6m FU: NR	SMD -0.08 (-0.33; 0.17), *I*^*2*^ = 35%, 6 studies (*n* = 391) Subgroup analysis (*I*^*2*^ NR): - smartphone-based video calls: SMD -0.01 (-0.25; 0.24), 3 studies - computer-based training SMD -0.38 (-1.39; 0.64), 3 studies
Shah 2021 [54] Loneliness Older adults (mean age 73–78 years), independent or assisted living	Social internet-based activities (via social websites, videoconferencing, customized computer platforms, WhatsApp groups, etc.) vs. TAU, no activity	Delivery: Group Mode: Digital F/D: NR/3-12m FU: 3m (2), 4m (2), 6m (2), 12m (1)	By time of FU: 3 months: SMD 0.02 (-0.36; 0.40), *I*^*2*^ = 0%, 3 studies 4 months: SMD -1.11 (-2.60; 0.38), *I*^*2*^ = 88%, 2 studies 6 months: SMD -0.11 (-0.54; 0.32), *I*^*2*^ = 37%, 2 studies
Reviews with narrative synthesis			
Choi 2021 [73] Loneliness Older adults (60 +)	ICT interventions: Robot animal (1), online interventions (support, information, maladaptive cognitions) (2) vs. TAU	Delivery: Individual Mode: F2F, internet F/D: NR/6-15w FU: NR	Robot animal: no evidence of effect Two online support interventions: evidence of effect, one of which showed effect maintained after 12 months (n’s = NR)
Forsman 2018 [71] Loneliness Older adults	Technology-based (ICT training, computer gaming, Nintendo Wii) vs. TAU, living dog	Delivery: Individual Mode: Internet F/D: NR FU: 3-98m (2)	No evidence of effect, except in one small study (*n *= 16 in intervention group) of Nintendo computer gaming 6 studies (*n* = 752)
Heins 2021 [72] Social isolation, loneliness Age 55 + with or without dementia	Technologically assisted (mobile app/web-based therapy, self-monitoring of physical activity, psychoeducation, health education) vs. Waitlist, no intervention, TAU	Delivery: Mix Mode: Internet F/D: NR/3-6m FU: 12w (1)	Social isolation: 1/2 trials showed evidence of effect (*n *= 110) Loneliness: 1/1 trial showed no evidence of effect (*n *= 60)
Li 2018 [74] Loneliness Older adults (mean age > 75)	Exergames – combining digital gaming (e.g., WII) and physical exercise vs. Other activities (board games, watching TV, normal exercise)	Delivery: Individual Mode: Internet F/D: 1–3 sessions weekly/4-12w FU: No	4/4 trials showed evidence of effect (*n* = 282)

*F2F* Face-to-face, *TAU* treatment as usual, *W* weeks, *M* months, *Y* years, *ES* Effect size, *N* number of participants, *g* Hedges’ g, *SMD* standardized mean difference, *NR* not reported, *RCT* Randomized controlled trial, *SI* Social isolation, *L* Loneliness, *LTC* Long-term care

^1^Not limited to a specific group. *Effect* indicates a significant (*p* < .05) effect in favor of the intervention

**Table 11:**
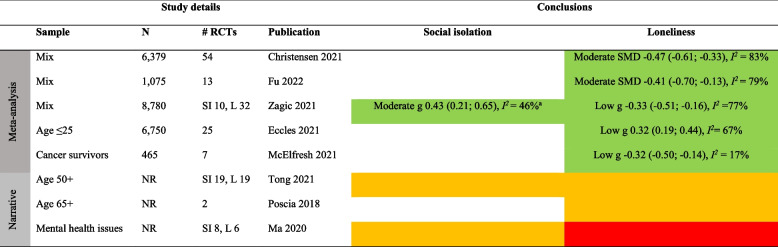
Summary of findings on the efficacy of diverse types of interventions to reduce social isolation and loneliness

Color keys: green = evidence of effect, yellow = inconsistent or inconclusive evidence of effect, red = no evidence of effect. *SI *Social isolation, *L* Loneliness

a Significant (p .05) only after removing one outlier

**Table 12:**
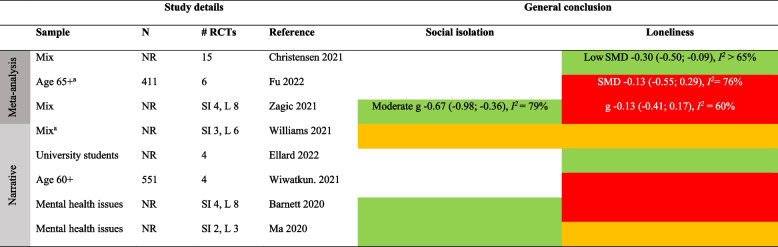
Summary of findings on the efficacy of social contact/network interventions to reduce social isolation and loneliness

Color keys: green = evidence of effect, yellow = inconsistent or inconclusive evidence of effect, red = no evidence of effect. *SI* Social isolation, *L* Loneliness

a Digital interventions

**Table 13:**
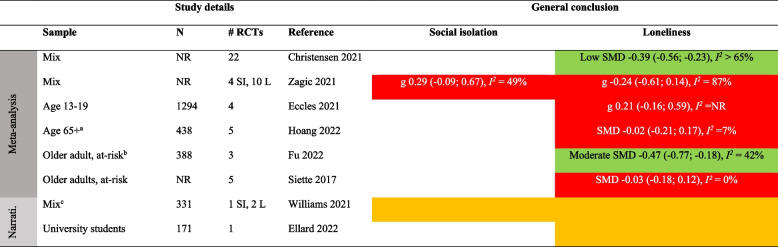
Summary of findings on the efficacy of social support interventions to reduce social isolation and loneliness

Color keys: green = evidence of effect, yellow = inconsistent or inconclusive evidence of effect, red = no evidence of effect. *SI* Social isolation, *L* Loneliness

a Community setting, b Digital interventions, c Delivered via phone

**Table 14:**
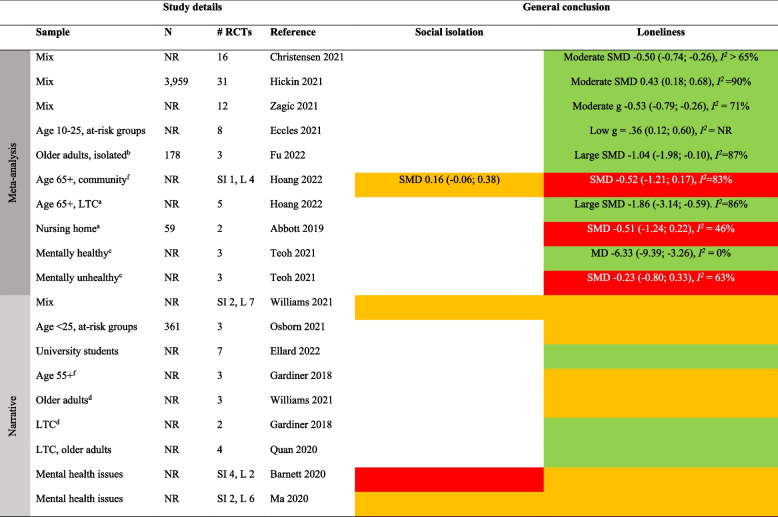
Summary of findings on the efficacy of psychological interventions to reduce social isolation and loneliness

Color keys: green = evidence of effect, yellow = inconsistent or inconclusive evidence of effect, red = no evidence of effect. *SI* Social isolation, *L* Loneliness

a Animal-assisted therapy, b Digital, c Group-based, d Pets, animal-assisted e Counseling (e.g., bereavement and lifestyle counseling), f Community setting

**Table 15:**
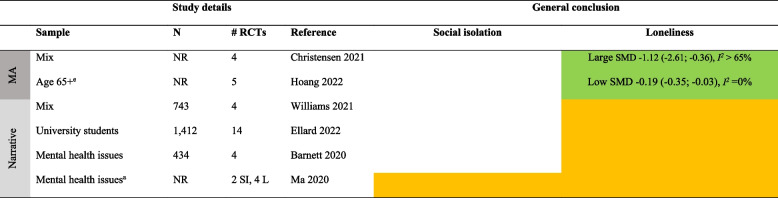
Summary of findings on the efficacy of psychoeducation interventions to reduce social isolation and loneliness

Color keys: green = evidence of effect, yellow = inconsistent or inconclusive evidence of effect, red = no evidence of effect. *SI* Social isolation, *L* Loneliness, *MA* Meta-analysis

a Social skills training and psychoeducation (in community setting)

**Table 16:**
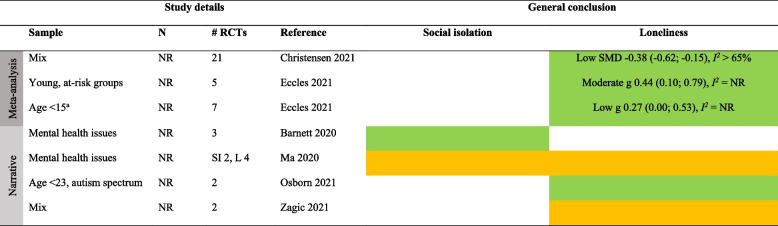
Summary of findings on the efficacy of social skills interventions to reduce social isolation and loneliness

Color keys: green = evidence of effect, yellow = inconsistent or inconclusive evidence of effect, red = no evidence of effect. *SI* Social isolation, *L* loneliness

a Social-emotional training

**Table 17:**
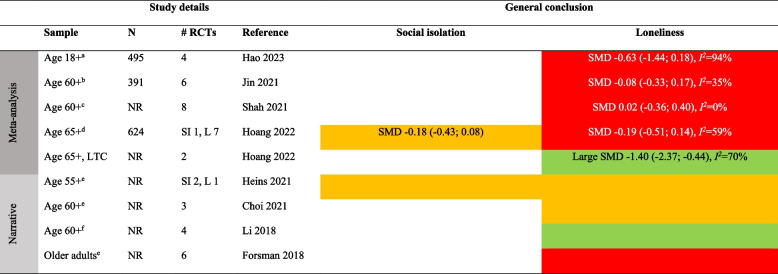
Summary of findings on the efficacy of digital interventions to reduce social isolation and loneliness

Color keys: green = evidence of effect, yellow = inconsistent or inconclusive evidence of effect, red = no evidence of effect. *LTC* Long-term care. *SI* Social isolation, *L* loneliness

a Online social contact, therapy, etc., b PC use, videocall, c online group call, d community setting, e ICT training, online support/therapy, f Exergames (online games/exercise)

We summarize the evidence for each type of intervention below. The category “structural interventions” is excluded from this summary due to a lack of findings. Furthermore, due to a substantial overlap in their respective constituent interventions, digital interventions and computer/internet interventions have been consolidated into a single category.

#### Mixed interventions

In some SRs, evidence derived from diverse types of intervention was analyzed through pooled meta-analysis or narrative synthesis precluding the possibility of structuring the evidence by intervention type. We identified 5 such SRs with meta-analyses [10, 32, 40, 56, 57] and 3 SRs with narrative syntheses [36, 58, 59] (Tables 4 and 11). The SRs with narrative synthesis focused on older adults [36, 58] or people with mental health problems [59]. These SRs show inconclusive evidence for social isolation [36, 59] and inconclusive or no evidence of an effect on loneliness [36, 58, 59].

The 5 SRs with meta-analyses focused on the general population [10, 40, 56], younger persons [32], or cancer survivors [57]. The analyses show small to moderate effects of the aggregated (pooled) interventions on social isolation [10] and loneliness [10, 32, 40, 56, 57]. The meta-analysis on social isolation found evidence of a moderate effect (g = 0.43 [0.21; 0.65], *I*^*2*^ = 46%, 10 RCTs) [10]. For loneliness, the largest meta-analysis covering 54 RCTs found a moderate effect (SMD = -0.47 [-0.61; -0.33], *I*^*2*^ = 83%), but also considerable heterogeneity.

Four SRs with meta-analysis of loneliness explored moderation effects (Table 4). With respect to sustainability of effects, one review reported moderate, considerably heterogeneous effects both in the short term (≤ 4 weeks; SMD -0.47 [-0.61; -0.33], 54 studies, *I*^*2*^ = 83%) and long-term (5–26 weeks; SMD -0.49 [-0.76; -0.23], 18 studies, *I*^*2*^ = NR) [40]. Another review of 13 studies found a small effect at < 3 months (SMD -0.33 [-0.52; -0.14], *I*^*2*^ < 50%) and at 3–6 months (SMD -0.32 [-0.57; -0.07], *I*^*2*^ > 50%), but not at > 6 months (SMD 0.37 [-0.02; 0.76], *I*^*2*^ = NR). Moreover, SRs showed no statistically significant variation in effects across age groups [40], delivery (group vs. individual) [40, 56], mode (face-to-face vs. digital) [40], or study quality [32, 40]. Finally, while the effect sizes tended to be highest and most often statistically significant for psychological and educational interventions, the moderating role of intervention type was not statistically significant [32, 56, 60].

#### Social contact/network interventions

These interventions often used an activity-based group format such as community groups, choirs, or exercise groups. These activities were typically delivered either in-person or through digital platforms, often scheduled on a weekly or bi-weekly basis, with a duration ranging from 4 to 52 weeks [7, 56].

We identified 3 SRs with meta-analysis [10, 40, 56] and 5 SRs with narrative synthesis [50, 59, 61–63] including social contact/network interventions (Tables 5 and 12). The SRs with narrative synthesis have focused on various populations [61], university students [63], older adults [62], or people with mental health problems [50, 59]. Of the three narrative syntheses for social isolation (each with ≤ 3 RCTs), two showed evidence of effect [50, 59] and one showed inconclusive results [61]. Of the five narrative syntheses of loneliness, three (each with ≤ 6 RCTs) showed inconclusive results [59, 61, 62], evidence of effect (4 RCTs) among university students [63], and (8 RCTs) no evidence of effect [50].

The three reviews with meta-analyses focused on all age groups [10, 40] or older adults [56]. One SR (4 RCTs) focused on social isolation and showed a considerably heterogeneous (*I*^*2*^ = 79%) moderate effect (g -0.67 [-0.98; -0.36]) [10]. Three SRs (6‒15 RCTs) reported on loneliness, and all showed substantial heterogeneity in the effects (*I*^*2*^ ≥ 60%). Two of the SRs found no evidence of effect [10, 56], and one found a small effect (SMD -0.30 [-0.50; -0.09]) [40]. None of the SRs addressed long-term effects.

#### Social support interventions

Social support interventions were primarily befriending efforts delivered individually. Typically, these interventions were facilitated by a volunteer and scheduled on a weekly or bi-weekly basis spanning up to one year. The mode of delivery varied, taking place either in-person or through digital platforms [32, 53].

Six meta-analyses and two narrative syntheses reported on social support interventions (Tables 6 and 13). As the narrative syntheses [61, 63] incorporated only 1–2 RCTs (*n* = 171 − 331) for each outcome, the conclusions drawn regarding the effects remain uncertain. Only one SR with meta-analysis (4 RCTs) focused on social isolation, showing a small non-significant effect (g 0.29 [-0.09; 0.67], *I*^*2*^ = 49%) within mixed populations [10].

Six SRs with meta-analysis addressed loneliness, of which two focused on populations of all ages based on 10 [10] and 22 [40] RCTs, one on younger people (4 RCTs) [32], and three on older adults (3–5 RCTs) [53, 56, 64]. The effects were inconsistent with four SRs showing no evidence of effects and two reporting small-moderate effects that were moderately to substantially heterogeneous (SMD -0.39 [-0.56; -0.23], *I*^*2*^ ≥ 65% [40]; SMD -0.47 [-0.77; -0.18], *I*^*2*^ = 42% [56]).

#### Psychological interventions

Psychological interventions most frequently involve cognitive-behavioral therapy (CBT) and mindfulness-based stress-reduction [50, 65]. A few used reminiscence therapy for older adults and animal-assisted therapy, where participants interacted with either live dogs or robotic animals such as seals or dogs [66]. Most interventions were delivered individually, with some opting for group settings. These were more often delivered face-to-face than digitally, according to the SRs providing such details (e.g., [32, 49, 56]). The frequency and duration of interventions varied as well, typically occurring weekly or biweekly, with durations ranging from a few weeks to up to a year. Follow-up effects were largely unaddressed.

Based on 15 SRs (some with multiple analyses for different subgroups), the evidence on psychological interventions included 10 meta-analyses and 9 narrative syntheses (Tables 7 and 14). Population groups varied widely across reviews, with five addressing a mixed population [10, 40, 49, 55, 61], three targeting younger people [32, 63, 67], five targeting older adults [41, 53, 56, 66, 68], and two focusing on people with mental health concerns [50, 59].

Four SRs focused on social isolation, all employing narrative synthesis [50, 53, 59, 61]. Based on 4 RCTs, these reviews presented inconclusive or no evidence of effects. Among the eight reviews with narrative syntheses on loneliness, conclusions were also mixed and based on few (2 − 7) RCTs. Some of the evidence showed effect, among university students [63] or older adults in long-term care [41, 68]. Others reported inconclusive evidence, in mixed populations [61], among people with mental health issues [50, 59], young adults [41, 67, 68], and older adults [41, 61].

Of the 10 SRs with meta-analyses on the effect of psychological interventions to reduce loneliness, seven showed benefits [10, 32, 40, 49, 53, 55, 56]. Three SRs based on few (2 − 4) RCTs reported small to moderate effects that were not significant [53, 55, 66]. The seven showing effects included up to 31 RCTs and the effects were generally moderate-large in size and substantially to considerably heterogeneous (*I*^*2*^ ≥ 65%). For example, the SR of 31 RCTs found a moderate effect (SMD 0.43 [0.18; 0.68, *I*^*2*^ = 90%) [49]. The power of the meta-analyses is crucial as despite the pooled analyses showing significant effects, approximately half of their constituent RCTs did not (see [32, 55, 66, 69]). Two SRs reported a GRADE certainty of the evidence; the resulting grades were 'low' [55] and 'moderate' [70].

#### Psychoeducation interventions

Psychoeducation interventions typically involved educating individuals at risk of loneliness (e.g., due to mental health issues) about topics relevant to loneliness or health more generally (Tables 8 and 15). These interventions were addressed in two meta-analyses [40, 53] and four narrative syntheses [50, 59, 61, 63]. Three SRs did not report population details, and others focused on younger people [63], older adults [53], or people with mental health problems [50, 59]. The narrative syntheses reported inconclusive evidence for benefits of psychoeducational interventions to reduced social isolation [59] or loneliness [50, 59, 61, 63]. The meta-analyses on the effects of psychoeducational interventions for loneliness were reported as having a small effect (SMD -0.19 [-0.35; -0.03], *I*^*2*^ = 0%) and a large effect (SMD -1.12 [-2.61; -0.36], *I*^*2*^ = 65%), the latter with substantial heterogeneity. The SRs contained sparse additional intervention details, and none included follow-up data.

#### Social skills interventions

Social skills interventions were primarily delivered in-person, adopting a group format, and typically held on a weekly basis. The duration of these interventions varied, ranging from six weeks to a year. These interventions were explored in two SRs [32, 40] with three meta-analyses and four SRs with narrative synthesis [10, 50, 59, 67] (Tables 9 and 16). Of these, two SRs included diverse populations [10, 40], two focused on people with mental health issues [50, 59], and two focused on younger people at risk of loneliness such as those diagnosed with Autism Spectrum Diagnosis (ASD), social phobia, or other mental health conditions [32, 67].

Two of the SRs focused on social isolation using narrative syntheses, each based on 2–3 RCTs. One reported inconclusive outcomes [59], and the other reported beneficial effects [50]. Three other SRs with narrative synthesis focused on effects on loneliness, with two reporting inconclusive evidence (based on 2–4 RCTs) [10, 59] and one reporting effects (2 RCTs) [67].

Three meta-analyses indicated the effect of social skills interventions on loneliness. One such analysis showed a small and considerably heterogeneous effect (SMD -0.38 [-0.62; -0.15], *I*^*2*^ > 65%), with the GRADE certainty of evidence rated as “moderate” [40]. One SR on young persons performed a separate analysis for “at-risk” groups (g 0.44 [0.10; 0.79], *I*^*2*^ NR, 5 RCTs) and for children under age 15 (g 0.27 [-0.01; 0.53], *I*^*2*^ NR, 7 RCTs), demonstrating a moderate effect of social skills interventions on loneliness in the former group [32].

#### Digital interventions

Eight SRs, each based on a few (1 − 8) RCTs, investigated digital interventions among older adults (Tables 10 and 17). These interventions included computer training, online interaction and support, gaming, and other internet-mediated approaches. These interventions were delivered both in groups and individually, usually with 1 − 3 sessions per week and for a period of one to six months [53, 71].

One SR, with two narrative syntheses based on 1 − 2 RCTs, focused on social isolation and showed inconclusive evidence [72]. Four SRs with narrative synthesis focused on loneliness. These SRs demonstrated varying outcomes. No evidence of effect was found from 6 RCTs on information and communication technology (ICT) training and gaming [71], inconclusive evidence was found based on 1 − 3 RCTs evaluating online support or therapy [72, 73], and evidence of effect was found on gaming and exercise (4 RCTs) [74]. Four SRs with meta-analysis showed no evidence of effect [53, 54, 75, 76], while one SR of two RCTs among individuals in long-term care showed a large and substantially heterogeneous effect (SMD -1.40 [-2.37; -0.44], *I*^*2*^ = 70%). Assessment of long-term effects was largely absent, with the exception of two SRs [54, 72] that included a total of 8 RCTs, and demonstrated no evidence of effects at various time points, up to one year.

#### Other interventions

We found five SRs that included a total of 10 analyses, including seven meta-analyses, of intervention types outside the above categories (see Appendix 7). Two SRs explored the effect of leisure and skill development, yielding inconclusive or null effects on social isolation and loneliness [32, 61]. One SR, adopting a narrative synthesis of two RCTs, found no effect of health and social care service interventions on loneliness [61]. A further SR with meta-analysis on the effects of group-based exercise interventions among older adults, found no effect on social isolation or loneliness [53]. One review examined music interventions, including choir participation and music therapy, and reported a small effect on loneliness (SMD -0.34 [-0.55; -0.13], 1 RCT), but no effect on social isolation (2 RCTs) [53]. Two SRs evaluated interventions that combined psychotherapy and exercise. One of the reviews focused on older adults and found no evidence of effect for either social isolation or loneliness [77]. The other SR involving young Chinese individuals reported a large effect (SMD -1.10 [-1.45; -0.71], 8 RCTs) on loneliness [65]. One SR assessed the effect of multicomponent interventions (various combinations) among older adults [53]. In community settings, there was a small effect on social isolation (SMD 0.29 [0.15; 0.43], *I*^*2*^ = 0%, 6 RCTs) and a moderate effect on loneliness (SMD -0.67 [-1.13; -0.21], *I*^*2*^ NR, 2 RCTs), and in long-term care settings, there was a moderate effect on loneliness (SMD -0.53 [-0.86; -0.20], *I*^*2*^ = 57%, 3 RCTs).

## Discussion

The aim of the present UR was to synthesize and critically appraise systematic reviews of RCT-based evidence on the effectiveness of SIL interventions. The evidence showed that social interventions promoting social contact and providing social support are effective strategies to tackle social isolation. In contrast, psychological interventions hold the most promise for mitigating loneliness. While previous URs have not been sufficiently detailed and comprehensive to support the former finding, there is some evidence supporting the latter [30, 31]. The quality of the evidence varies greatly, and effect sizes are typically being small to moderate and displaying substantial to considerable heterogeneity. The heterogeneity can be attributed to multiple factors, including varying intervention components such as frequency, duration, setting, and content, as well as methodological limitations such as risk of bias and small sample sizes. There was no reporting that interventions did any harm, but we are uncertain whether the primary studies measured any adverse events, or if this is an oversight from SRs authors.

The analysis revealed a nuanced landscape, with our results both aligning with and diverging from previous URs; however, direct comparisons are complicated due to these reviews' tendency to aggregate data across various types of interventions, outcomes (loneliness and social isolation), and study designs (RCTs and-non-RCTs). Additionally, only three previous URs covering all ages and intervention types focus on loneliness [29–31] and only one focuses on social isolation [30], limiting the comparison material. While these URs generally concluded that most interventions either show an effect [30] or no effect [29], our findings, along with one other UR [31] demonstrated effects of certain interventions, suggesting a more varied efficacy dependent on specific intervention type and targeted outcome. We have enriched our understanding by encompassing recent evidence and by also factoring in social isolation, which, despite its health impacts mirroring those of loneliness, has received far less attention in prior URs. By narrowing our focus to RCTs — often hailed as the "gold standard" and highest level of evidence — and supplementing with grey literature to potentially capture a broader scope of evidence, we aimed to strengthen the overall evidence base.

Why and how social interventions hold particular promise for mitigating social isolation can be offered several interpretations. Substantial evidence suggests that interventions aimed at providing social support or fostering friendships and social activity show promise in reducing social isolation, especially in the short term [10, 50, 61]. However, the long-term impacts remain uncertain [59]. Sustainability is critical, as the immediate effect can be deemed self-evident or even tautological; the presence of social support inherently implies a reduction in social isolation.^4^

Although some well-powered meta-analyses with many RCTs and participants show small positive impacts of social interventions also on loneliness, the overall evidence shows no or inconsistent effects, suggesting a lesser and more uncertain impact on loneliness. This uncertainty suggests that increasing social contact does not necessarily alleviate loneliness unless accompanied by psychological changes [10]. Issues such as mistrust, negative self-beliefs, hypersensitivity to social threat and rejection, and social anxiety often intertwine with or underpin loneliness [59], hindering the formation and maintenance of close social relationships. For some, new social situations might bring discomfort and self-consciousness, potentially intensifying feelings of isolation rather than mitigating loneliness. Likewise, while compassionate social support and companion resources in befriending interventions can yield significant anticipatory and experienced rewards for some, as qualitative studies indicate [64, 78], for others, it may highlight, stigmatize, and patronize their loneliness, exacerbating rather than reducing it. These ideas suggest that interventions designed to foster social relationships within a safe setting may not be effective unless they also address the underlying psychological causes of loneliness [33, 61].

Why and how psychological and educational interventions show more potential for reducing loneliness than social isolation can be interpreted in various ways. The moderate to large positive effects often exhibited for psychological interventions on loneliness may reflect that they address the cognitive-behavioral underlying roots of loneliness. Psychological-educational interventions address some of the same barriers and components, focusing on providing lessons on making friends and addressing barriers to social integration [49]. Part of the success may also stem from the fact that the interventions target cognitive biases and avoidance behavior that underlie not only loneliness, but also mental health problems that co-exist with or underpin loneliness, such as depressive mood, anxiety, low self-worth, and social withdrawal [7]. Hence, the effect may be indirect by targeting the barriers to secure social connection. Their success likely also stems from being directed mainly or exclusively toward individuals who are either lonely or at risk of loneliness due to underlying psychological issues or conditions (e.g., ASD).

The evidence for the effects from other types of interventions was small and inconsistent. For instance, the few SRs centered on physical activities, learning new hobbies, and health and social care services reported no evidence of effect. Digital interventions have increased in popularity in recent years and may help people stay connected with family and friends and access information or receive social support in online communities. To date, and as found in previous URs [26–28, 31], the evidence on their effectiveness for addressing SIL is uncertain. This uncertainty could reflect the highly diverse nature of digital interventions, and the fact that they have targeted older adults. While older adults are becoming more adept with technology and online communication, a significant portion may still face challenges navigating these platforms and might find such communication unsatisfying [79].

Moderation effects and subgroup heterogeneity were examined in mixed types of interventions. Only a few SRs examined longer-term effects and the results were inconsistent. For example, while one SR demonstrated that long-term effects (i.e., one to six months after the intervention) were comparable to the short-term effects (i.e., up to four weeks after the intervention) [7], another SR found evidence of effect for up to 6 months but not thereafter [56]. Furthermore, no difference in the overall effectiveness was shown for group vs. individual settings [30, 32, 40, 56, 60], between digital and non-digital interventions [7, 32, 40], between studies of high, moderate and low quality [7, 32, 40], or depending on age groups [7, 32, 40] or gender [32]. The relative similarity of effects across subgroups implies that various approaches and strategies can be employed to reduce loneliness without a significant difference in outcomes.

The overlap of primary studies in the results can introduce redundancy and potentially skew findings. However, in the current overview, the impact appears to be minimal due to the low degree of overlap, ensuring that the results remain predominantly independent.

### Limitations of the research evidence and implications for future research

This UR reveals several gaps and limitations in the literature, indicating areas for future research and interventions. For instance, studies need to assess interventions for young people, the persistence of intervention effects, and social isolation, which parallels loneliness in health impacts. It also underscores the urgent need to explore broader structural determinants and interventions of SIL [15, 80]. The lack of large and consistent findings may suggest that loneliness, rooted in social and structural conditions, cannot be effectively tackled through individual-level interventions alone. Instead it requires addressing broader societal causes like social inequalities, systemic marginalization, and neoliberal policies that worsen isolation and inequity [81]. Evaluations might benefit from observational studies or natural experiments, as RCTs may not suit these approaches. The absence of structural interventions in our UR could stem from our focus on RCTs, but notably, no such interventions were found during our broad screening across all study designs.

Beyond structural interventions, other types of interventions should be explored in future RCTs. For instance, “social prescribing” interventions, which have shown promise in non-RCT trials [30], could offer valuable insights into tackling social isolation and loneliness. Additionally, it is worth noting the potency of giving support to tackle one’s own social disconnection. Positive psychology interventions have long recognized that giving (generosity, prosociality) is often more powerful than receiving, challenging the assumption that people must always be on the receiving end of an intervention to address SIL. Volunteering and digital interventions, through group interactions and online forums, offer unique and flexible opportunities for reciprocal support, fostering meaningful connections and positive relationships.

Furthermore, trials often lack clear theoretical bases, hindering the identification of active elements that reduce loneliness. For instance, in mindfulness-based therapies, the effective factor is unclear and could be meditation, breath-work, presence, group interaction, or increased thought awareness. Similarly, it is often unclear whether interventions target lonely individuals or those presumed at risk [32, 49, 53], requiring more clarity in future intervention design and evaluation [29].

Another limitation we encountered is the quality of the trials and systematic reviews. Many trials lack adequate blinding procedures, randomization processes, and power, increasing the risk of erroneously inferring the presence of an effect (type-I error). Furthermore, the quality of SRs is often low. Many reviews fail to preregister protocols, to use scientific quality appropriately in formulating conclusions, to specify interventions in adequate detail, and to use clear categories of interventions [58, 68, 69]. These problems partly reflect the lack of detail reported in the primary studies. Meta-analysis on pooled data of highly diverse types of interventions is also problematic, as evidenced by the substantial heterogeneity of their effects. Such an approach also curtails the applicability of the results for practical purposes.

Furthermore, combining dissimilar interventions in meta-analyses compromises the practical relevance of the pooled estimates due to the diversity of interventions [82]. Although some SRs include subgroup analyses, the lack of differentiation by intervention types limits the practical application of the acquired knowledge [10]. Additionally, narrative synthesis within the SRs relied excessively on *p*-values, whereas reporting numerical data, effect sizes, and precision are preferable [29]. We frequently only had the number of trials with significant effects (“vote count”) to rely on. It is generally recommended to avoid “vote counting”, e.g., counting effects that are statistically significant and favoring the intervention vs. all others [47]. This method has limitations and can lead to incorrect conclusions because underpowered studies that do not rule out clinically important effects are counted as not showing benefit. Additionally, it does not provide information on the magnitude of effects and does not account for differences in the relative sizes of the studies. The absence of inter-rater reliability calculations for the full-text review, data extraction, and quality appraisal is a potential limitation of our methodology. However, we took significant steps to ensure reliability in our coding process. All coders received extensive training and participated in calibration exercises before beginning the assessments. Additionally, the coding team, drawn from the same work group, brought a wealth of experience from similar projects, fostering a consistent approach. Our observations showed that ratings from different coders were remarkably consistent.

Integrating elements from different approaches to treat individuals holistically and individually is another avenue for future research. Given the heterogeneity of the population of lonely people, it is essential to tailor interventions to different types of loneliness, triggers, and risk groups [29, 53]. For instance, those whose loneliness is rooted in insecure attachment or mental health issues might need interventions focusing on cognitive and other barriers. On the other hand, people with situational loneliness may benefit from interventions aimed at enhancing social networks and connectedness. In response to the question of common misconceptions about ways to enhance happiness, bestselling author Gretchen Rubin asserted that the fundamental mistake people make is to believe that there is a single, universally effective method [83]. She emphasized that happiness-enhancing strategies are profoundly individualistic, contingent on one's unique nature, interests, values, and idiosyncrasies. This perspective mirrors our own and others’ (e.g., [10, 60]) notions regarding mitigating SIL, dispelling the idea of a one-size-fits-all solution and instead advocating for tailored and individual-centric strategies. In the rapidly evolving digital era, the exploration of ways to improve technology-based interventions for social isolation and loneliness becomes increasingly vital.

Balancing these limitations were several strengths, including the use of rigorous methods and quality assessment, grouping SRs by type of intervention and outcome, the exclusive focus on RCTs, and the broad search strategy including grey literature to contribute valuable insights to the field and inform future research and practice in addressing social isolation and loneliness.

## Conclusion

There is an urgent need to develop a comprehensive, evidence-based understanding and effective remedies for SIL. However, the current evidence from SRs since 2017 does not yet clearly support any specific intervention to reduce SIL. Potential interventions such as cognitive modification for loneliness and support and facilitated socialization for social isolation show promise, but the quality of published trials and SRs limits our confidence in their findings and our ability to draw firm conclusions. Compounding this uncertainty is the inconsistency within the findings, paired with our limited insight regarding the exact 'active ingredients' that bring about successful results, the interventions’ relevance to different subgroups, and the circumstances under which they perform optimally. We suggest that high-quality research and innovation in intervention development informed by the limitations identified in this UR should be prioritized. Critically, the customization of interventions based on the specific type and underlying cause of loneliness appears to be crucial for the development of efficacious strategies. Incorporating elements from various approaches—such as therapeutic counseling and social interaction—may offer a more holistic and effective solution. Digital platforms could serve as a valuable facilitator for these tailored interventions, enabling easier implementation and potentially reaching a wider audience.

### Supplementary Information

Supplementary Material 1.

## Data Availability

The data used in the study can be made available from T. H. (corresponding author) on reasonable request.

## Footnotes

1 Clearly defined inclusion criteria regarding population, type of intervention, and outcomes. We did not require a clearly defined comparator, given their highly diverse nature in this field.

2 The form we created for data extraction included explanations for each item and their categories. We piloted the form with two systematic reviews to ensure authors consistently extracted the same data using the same categories for the variables of interest.

3 Three SRs [10, 32, 47] express reductions in SIL with positively valanced effect sizes.

4 Social interventions spanned a few weeks to a maximum of one year in duration. However, data on long-term effects and post-intervention follow-ups was largely unavailable.
