# Supplementary material for: Tackling social disconnection: an umbrella review of RCT-based interventions targeting social isolation and loneliness
Source: BMC Public Health. 2024 Jul 17;24:1917. doi: 10.1186/s12889-024-19396-8 (PMC11256365; doi:10.1186/s12889-024-19396-8)
Supplement: Supplementary file 1 — Supplementary Material 1. [file 12889_2024_19396_MOESM1_ESM.docx]

**Appendix**

**“Tackling social disconnection: An umbrella review of RCT-based interventions targeting social isolation and loneliness”**

**Authors:** Thomas Hansen, Ragnhild Bang Nes (PhD), Kamila Hynek, Thomas Sevenius Nilsen, Anne Reneflot, Kim Stene-Larsen, Ragnhild Agathe Tornes, & Julia Bidonde^.^

**Outline**

1. Description of prior and current umbrella reviews
2. PRIOR guidelines
3. Search strategy
4. List of excluded studies and reasons for exclusion + List of records not found
5. Quality appraisals using the AMSTAR2 tool
6. Assessment of overlap
7. Results from “other types of interventions”

**Appendix 1**. Descriptions of prior and current umbrella reviews (UR) on interventions to reduce loneliness and/or social isolation

| **Publication (* = peer reviewed)** | **Intervention details** | **Population** | **Designs included** | **Outcome** | **Overall conclusion** |
| --- | --- | --- | --- | --- | --- |
| Chipps 2017 (3)* | Digital | Older adults | Mixed design (RCTs and non-RCTs) | Loneliness/social isolation^a^ | There was little or no difference for digital interventions so it is uncertain whether digital interventions (e.g., online activities, internet-supported communication, internet/computer training) decrease loneliness. |
| Victor 2018 (6) | All types | General | RCTs | Loneliness | There was no evidence of effect on loneliness of interventions, irrespective of setting (care home or community), mode of delivery (individual or group), or intervention type. The authors relate the lack of effect to the small scale and short-term nature of the included interventions. |
| Beckers 2020 (1) | All types | General | Mixed design (RCTs and non-RCTs) | Loneliness/social isolation^a^ | There was evidence that most intervention types (e.g., digital, psychological and skills training, animal interaction, physical activity, social prescribing, place-based interventions) probably decrease loneliness. The authors note that it is not possible to identify one superior type of intervention. Effect sizes and heterogeneity of effects sizes are not discussed. |
| Boulton 2020 (2) | Digital | Older adults | Mixed design (RCTs and non-RCTs) | Loneliness and social isolation | Video-communication interventions may decrease loneliness and social isolation. Telephone befriending, online discussion groups and forums, social networking sites, and multi-tool interventions may lead to little or no difference in decreasing loneliness and social isolation. Effect sizes and heterogeneity of effect sizes are not discussed. |
| Jarvis 2020 (4)^b^ | All types | Older adults | Mixed design (RCTs and non-RCTs) | Loneliness and social isolation | No evidence of effect on loneliness irrespective of delivery format (digital or non-digital) or intervention type (social support, social contact, social skills training). Social cognition may decrease loneliness, but this evidence was based on only one RCT. No synthesis provided for effects on social isolation due to its inconsistent definitions and operationalizations across primary studies. |
| Veronese 2021 (5)^b^ | All types | General | RCTs | Loneliness | Meditation/mindfulness, social cognitive training, and social support interventions may slightly decrease loneliness. There was evidence of no effect for befriending, digital interventions or social training decreasing loneliness; thus, evidence is uncertain about these interventions’ effectiveness in decreasing loneliness. |
| Hansen 2023 | All types | General | RCTs | Loneliness and social isolation | Social interventions may lead to a slight increase in social isolation (low to moderate effect with high heterogeneity). Psychological and educational interventions may decrease loneliness (low to moderate effect with high heterogeneity). There is uncertainty about the effects of digital interventions and physical activity in decreasing loneliness and social isolation. |

^a^ While the search is done for both ‘social isolation’ and ‘loneliness’, in the synthesis of results they are treated synonymously and not analyzed separately.
^b^ The UR reviews and (when possible) meta-analyzes evidence from primary studies selected from published systematic reviews.

**Appendix 2.** PRIOR checklist (preferred reporting items for overviews of reviews)

| Section topic | Item No | Item | Location where item is reported |
| --- | --- | --- | --- |
| **Title** | | |  |
| Title | 1 | Identify the report as an overview of reviews. | p. 1 |
| **Abstract** | | |  |
| Abstract | 2 | Provide a comprehensive and accurate summary of the purpose, methods, and results of the overview of reviews. | p. 1 |
| **Introduction** | | |  |
| Rationale | 3 | Describe the rationale for conducting the overview of reviews in the context of existing knowledge. | Intro, paragraph 4-6 |
| Objectives | 4 | Provide an explicit statement of the objective(s) or question(s) addressed by the overview of reviews. | Intro, paragraph 6 |
| **Methods** | | |  |
| Eligibility criteria | 5a | Specify the inclusion and exclusion criteria for the overview of reviews. If supplemental primary studies were included, this should be stated, with a rationale. | Methods, paragraph 2 |
|  | 5b | Specify the definition of “systematic review” as used in the inclusion criteria for the overview of reviews. | Methods, paragraph 2 |
| Information sources | 6 | Specify all databases, registers, websites, organisations, reference lists, and other sources searched or consulted to identify systematic reviews and supplemental primary studies (if included). Specify the date when each source was last searched or consulted. | Methods, paragraph 3 |
| Search strategy | 7 | Present the full search strategies for all databases, registers and websites, such that they could be reproduced. Describe any search filters and limits applied. | Methods, paragraph 3 |
| Selection process | 8a | Describe the methods used to decide whether a systematic review or supplemental primary study (if included) met the inclusion criteria of the overview of reviews. | Methods, paragraph 2 & 4 |
|  | 8b | Describe how overlap in the populations, interventions, comparators, and/or outcomes of systematic reviews was identified and managed during study selection. | Methods, paragraph 8 |
| Data collection process | 9a | Describe the methods used to collect data from reports. | Methods, paragraph 5 |
|  | 9b | If applicable, describe the methods used to identify and manage primary study overlap at the level of the comparison and outcome during data collection. For each outcome, specify the method used to illustrate and/or quantify the degree of primary study overlap across systematic reviews. | N/A |
|  | 9c | If applicable, specify the methods used to manage discrepant data across systematic reviews during data collection. | N/A |
| Data items | 10 | List and define all variables and outcomes for which data were sought. Describe any assumptions made and/or measures taken to identify and clarify missing or unclear information. | Methods, paragraph 5 |
| Risk of bias assessment | 11a | Describe the methods used to assess risk of bias or methodological quality of the included systematic reviews. | Methods, paragraph 7 |
|  | 11b | Describe the methods used to collect data on (from the systematic reviews) and/or assess the risk of bias of the primary studies included in the systematic reviews. Provide a justification for instances where flawed, incomplete, or missing assessments are identified but not reassessed. | Methods, paragraph 2 |
|  | 11c | Describe the methods used to assess the risk of bias of supplemental primary studies (if included). | N/A |
| Synthesis methods | 12a | Describe the methods used to summarise or synthesise results and provide a rationale for the choice(s). | Methods, paragraph 9 & 10 |
|  | 12b | Describe any methods used to explore possible causes of heterogeneity among results. | N/A |
|  | 12c | Describe any sensitivity analyses conducted to assess the robustness of the synthesised results. | N/A |
| Reporting bias assessment | 13 | Describe the methods used to collect data on (from the systematic reviews) and/or assess the risk of bias due to missing results in a summary or synthesis (arising from reporting biases at the levels of the systematic reviews, primary studies, and supplemental primary studies, if included). | Methods, paragraph 5 & 6 |
| Certainty assessment | 14 | Describe the methods used to collect data on (from the systematic reviews) and/or assess certainty (or confidence) in the body of evidence for an outcome. | Methods, paragraph 5 |
| Results | | |  |
| Systematic review and supplemental primary study selection | 15a | Describe the results of the search and selection process, including the number of records screened, assessed for eligibility, and included in the overview of reviews, ideally with a flow diagram. | Results, paragraph 1 |
|  | 15b | Provide a list of studies that might appear to meet the inclusion criteria, but were excluded, with the main reason for exclusion. | Results, paragraph 1 |
| Characteristics of systematic reviews and supplemental primary studies | 16 | Cite each included systematic review and supplemental primary study (if included) and present its characteristics. | Results, paragraph 5 |
| Primary study overlap | 17 | Describe the extent of primary study overlap across the included systematic reviews. | Results, paragraph 8 |
| Risk of bias in systematic reviews, primary studies, and supplemental primary studies | 18a | Present assessments of risk of bias or methodological quality for each included systematic review. | Results, paragraph 2 & 3 |
|  | 18b | Present assessments (collected from systematic reviews or assessed anew) of the risk of bias of the primary studies included in the systematic reviews. | Results, paragraph 4 |
|  | 18c | Present assessments of the risk of bias of supplemental primary studies (if included). | N/A |
| Summary or synthesis of results | 19a | For all outcomes, summarise the evidence from the systematic reviews and supplemental primary studies (if included). If meta-analyses were done, present for each the summary estimate and its precision and measures of statistical heterogeneity. If comparing groups, describe the direction of the effect. | Results, paragraph 9-31 |
|  | 19b | If meta-analyses were done, present results of all investigations of possible causes of heterogeneity. | N/A |
|  | 19c | If meta-analyses were done, present results of all sensitivity analyses conducted to assess the robustness of synthesised results. | N/A |
| Reporting biases | 20 | Present assessments (collected from systematic reviews and/or assessed anew) of the risk of bias due to missing primary studies, analyses, or results in a summary or synthesis (arising from reporting biases at the levels of the systematic reviews, primary studies, and supplemental primary studies, if included) for each summary or synthesis assessed. | Results, paragraph 2-4 |
| Certainty of evidence | 21 | Present assessments (collected or assessed anew) of certainty (or confidence) in the body of evidence for each outcome. | Results, paragraph 10 & 24 |
| Discussion | | |  |
| Discussion | 22a | Summarise the main findings, including any discrepancies in findings across the included systematic reviews and supplemental primary studies (if included). | Discussion, paragraph 1 |
|  | 22b | Provide a general interpretation of the results in the context of other evidence. | Discussion, paragraph 1 |
|  | 22c | Discuss any limitations of the evidence from systematic reviews, their primary studies, and supplemental primary studies (if included) included in the overview of reviews. Discuss any limitations of the overview of reviews methods used. | Discussion, paragraph 8-12 |
|  | 22d | Discuss implications for practice, policy, and future research (both systematic reviews and primary research). Consider the relevance of the findings to the end users of the overview of reviews, eg, healthcare providers, policymakers, patients, among others. | Discussion, paragraph 2-6. 8-12, and Conclusion |
| Other information | | |  |
| Registration and protocol | 23a | Provide registration information for the overview of reviews, including register name and registration number, or state that the overview of reviews was not registered. | Methods, paragraph 1 |
|  | 23b | Indicate where the overview of reviews protocol can be accessed, or state that a protocol was not prepared. | Methods, paragraph 1 |
|  | 23c | Describe and explain any amendments to information provided at registration or in the protocol. Indicate the stage of the overview of reviews at which amendments were made. | N/A |
| Support | 24 | Describe sources of financial or non-financial support for the overview of reviews, and the role of the funders or sponsors in the overview of reviews. | Declarations |
| Competing interests | 25 | Declare any competing interests of the overview of reviews' authors. | Declarations |
| Author information | 26a | Provide contact information for the corresponding author. | Declarations |
|  | 26b | Describe the contributions of individual authors and identify the guarantor of the overview of reviews. | Declarations |
| Availability of data and other materials | 27 | Report which of the following are available, where they can be found, and under which conditions they may be accessed: template data collection forms; data collected from included systematic reviews and supplemental primary studies; analytic code; any other materials used in the overview of reviews. | Declarations |

**Appendix 3** Search strategies

**LONELINESS – OVERVIEW OF OVERVIEWS**

| **Contact person:** | Thomas Hansen |
| --- | --- |
| **Search:** | Ragnhild Agathe Tornes |
| **Peer review:** | Marita Heintz |
| **Duplicate control in EndNote:** | Before duplicate control: 3707  After duplicate control: 1887 |

**Database:** Ovid MEDLINE(R) and Epub Ahead of Print, In-Process, In-Data-Review & Other Non-Indexed Citations, Daily and Versions <1946 to June 16, 2022>

**Date:** 17.06.22

**Number of hits:** 731

| 1 | Loneliness/ or Social Isolation/ | 19903 |
| --- | --- | --- |
| 2 | (loneliness or lonely or (social* adj (exclusion? or excluded or isolation or isolated))).tw,kf. | 23468 |
| 3 | 1 or 2 | 34929 |
| 4 | limit 3 to "reviews (maximizes specificity)" | 800 |
| 5 | Meta-Analysis/ or Network Meta-Analysis/ or ((systematic* adj2 review*) or metaanal* or "meta anal*" or (review and ((structured or database* or systematic*) adj2 search*)) or "integrative review*" or (evidence adj2 review*)).tw,kf,bt. | 448581 |
| 6 | 4 or (3 and 5) | 1067 |
| 7 | limit 6 to yr="2017 -Current" | 731 |

**Database:** Embase <1974 to 2022 June 16> (via Ovid)

**Date:** 17.06.22

**Number of hits:** 847

| 1 | loneliness/ or social Isolation/ | 38381 |
| --- | --- | --- |
| 2 | (loneliness or lonely or (social* adj (exclusion? or excluded or isolation or isolated))).tw,kf. | 29351 |
| 3 | 1 or 2 | 49393 |
| 4 | limit 3 to "reviews (maximizes specificity)" | 960 |
| 5 | exp Meta-Analysis/ or "systematic review"/ or ((systematic* adj2 review*) or metaanal* or "meta anal*" or (review and ((structured or database* or systematic*) adj2 search*)) or "integrative review*" or (evidence adj2 review*)).tw,kf. | 644343 |
| 6 | 4 or (3 and 5) | 1683 |
| 7 | limit 6 to yr="2017 -Current" | 1106 |
| 8 | limit 7 to (conference abstracts or embase) | 847 |

**Database:** APA PsycInfo <1806 to June Week 2 2022> (via Ovid)

**Date:** 17.06.22

**Number of hits:** 480

| 1 | Loneliness/ or Social Isolation/ | 13483 |
| --- | --- | --- |
| 2 | (loneliness or lonely or (social* adj (exclusion? or excluded or isolation or isolated))).tw. | 26095 |
| 3 | 1 or 2 | 30022 |
| 4 | limit 3 to "reviews (maximizes specificity)" | 819 |
| 5 | (meta analysis or "systematic review").md. or meta analysis/ or ((systematic* adj2 review*) or metaanal* or "meta anal*" or (review and ((structured or database* or systematic*) adj2 search*)) or "integrative review*" or (evidence adj2 review*)).tw. | 98763 |
| 6 | 4 or (3 and 5) | 1058 |
| 7 | limit 6 to yr="2017 -Current" | 480 |

**Database:** Sociological Abstracts (via ProQuest)

**Date:** 16.06.22

**Number of hits:** 87

| S1 | Searched for: SU.EXACT("Loneliness" or "Social Isolation") | 4700 |
| --- | --- | --- |
| S2 | Searched for: AB,TI(loneliness or lonely or (social* P/0 (exclusion or exclusions or excluded or isolation or isolated))) | 13108 |
| S3 | Searched for: S1 or S2 | 14142 |
| S4 | Searched for: SU.EXACT("Technology Assessment") OR SU.EXACT("Literature Reviews") or AB,TI((systematic* N/1 review*) or metaanal* or "meta anal*" or (review and ((structured or database* or systematic*) N/1 search*)) or "integrative review*" or (evidence N/1 review*)) | 14395 |
| S5 | Searched for: S3 and S4 | 164 |
| S6 | Searched for: (S3 and S4) AND pd(20170101-20220616) | 87 |

**Database:** Cinahl (via Ebsco)

**Date:** 17.06.22

**Number of hits:** 393

| S8 | S7  Limiters - Exclude MEDLINE records | 393 |
| --- | --- | --- |
| S7 | S6  Limiters - Published Date: 20170101-20220631 | 512 |
| S6 | S4 or (S3 and S5) | 842 |
| S5 | (MH "Meta Analysis") OR (MH "Systematic Review") or TI ((systematic* N1 review*) or metaanal* or "meta anal*" or (review and ((structured or database* or systematic*) N1 search*)) or "integrative review*" or (evidence N1 review*)) OR AB ((systematic* N1 review*) or metaanal* or "meta anal*" or (review and ((structured or database* or systematic*) N1 search*)) or "integrative review*" or (evidence N1 review*)) | 229,400 |
| S4 | S3  Limiters - Clinical Queries: Review - High Specificity | 476 |
| S3 | S1 OR S2 | 21,585 |
| S2 | TI(loneliness or lonely or (social* W0 (exclusion# or excluded or isolation or isolated))) or AB(loneliness or lonely or (social* W0 (exclusion# or excluded or isolation or isolated))) | 13,427 |
| S1 | (MH "Loneliness") or (MH "Social Isolation") | 14,691 |

**Database:** Web of Science Core Collection: Science Citation Index Expanded (SCI-EXPANDED) --1987-present, Social Sciences Citation Index (SSCI) --1987-present, Arts & Humanities Citation Index (A&HCI) --1987-present, Emerging Sources Citation Index (ESCI) --2015-present

**Date:** 17.06.22

**Number of hits:** 461

| 4 | #3 and PY=2017-2022 | \| Exact search | 461 |
| --- | --- | --- | --- |
| 3 | #1 AND #2 | \| Exact search | 579 |
| 2 | TS=(("systematic*" NEAR/1 "review*") or ("review" and (("structured" or "database*" or "systematic*") NEAR/1 "search*")) or "integrative review*" or ("evidence" NEAR/1 "review*")) OR TI=("metaanal*" or "meta anal*") OR AB=("metaanal*" or "meta anal*") | \| Exact search | 491,731 |
| 1 | TS= (loneliness or lonely or (social* W0 (exclusion$ or excluded or isolation or isolated))) | \| Exact search | 19,902 |

**Database:** Cochrane Database of Systematic Reviews

Issue 6 of 12, June 2022

**Date:** 17.06.22

**Number of hits:** 11 (10 reviews, 1 protocol)

| #1 | [mh ^Loneliness] | 152 |
| --- | --- | --- |
| #2 | (loneliness or lonely or (social* NEXT (exclusion? or excluded or isolation or isolated))):ti,ab | 1698 |
| #3 | #1 or #2 | 1711 |
| #4 | #3 in Cochrane Reviews and Cochrane Protocols | 28 |
| #5 | #4 with Cochrane Library publication date Between Jan 2017 and Jun 2022, in Cochrane Reviews, Cochrane Protocols | 11 |

**Database:** Epistemonikos

**Date:** 17.06.22

**Number of hits:** 697

Publication type: Broad Synthesis: 118

Publication type: Structured Summary: 0

Publication type: Systematic Review: 579

**Note:** Simplified search strategy due to the limited search functionality

Title/Abstract: (loneliness or lonely or "social exclusion" or "social exclusions" or "socially excluded" or "social isolation" or "socially isolated")

Year: 2017-2022

Loneliness and social isolation – grey literature

Effects of interventions on loneliness and social isolation

**Contact person:** Thomas Hansen

**Search:** Ragnhild Agathe Tornes

**Date:** June 20 and 21 2022

**Duplicate control in EndNot**e: Before duplicate control: 280

After duplicate control: 239

| DATABASE | DATE | SEARCH | HITS |
| --- | --- | --- | --- |
| Swemed+  <https://svemedplus.kib.ki.se/> | June 20 2022 | \| Söksträng: \| Antall träffar \| \| --- \| --- \| \| exp:"loneliness" AND year:[2017 TO 2019] Limits: doctype:"översikt" \| 0 \| \| exp:"Social Isolation" AND year:[2017 TO 2019] Limits: doctype:"översikt" \| 1 \| \| loneliness Limits: doctype:"översikt" AND year:[2017 TO 2019] \| 0 \| \| lonely Limits: doctype:"översikt" AND year:[2017 TO 2019] \| 9 \| \| "social exclusion" AND year:[2017 TO 2019] Limits: doctype:"översikt" \| 0 \| \| "social exclusions" AND year:[2017 TO 2019] Limits: doctype:"översikt" \| 1 \| \| "social excluded" AND year:[2017 TO 2019] Limits: doctype:"översikt" \| 0 \| \| social isolation Limits: doctype:"översikt" AND year:[2017 TO 2019] \| 1 \| \| "socially isolated" AND year:[2017 TO 2019] Limits: doctype:"översikt" \| 1 \| | 10 hits without intern dupliquates |
| [Prospero](https://www.crd.york.ac.uk/PROSPERO/)  <https://www.crd.york.ac.uk/PROSPERO/> | June 20 2022 | \| Line \| Search for \| Hits \| \| --- \| --- \| --- \| \| #1 \| MeSH DESCRIPTOR loneliness AND (Review_Ongoing):RS AND (Intervention):RT WHERE CD FROM 2017 TO 20/06/2022 \| 0 \| \| #2 \| MeSH DESCRIPTOR social isolation AND (Review_Ongoing):RS AND (Intervention):RT WHERE CD FROM 2017 TO 20/06/2022 \| 0 \| \| #3 \| loneliness AND (Review_Ongoing):RS AND (Intervention):RT \| 121 \| \| #4 \| social isolation AND (Review_Ongoing):RS AND (Intervention):RT \| 106 \| \| #5 \| socially isolated AND (Review_Ongoing):RS AND (Intervention):RT \| 11 \| \| #6 \| social exclusion AND (Review_Ongoing):RS AND (Intervention):RT \| 15 \| \| #7 \| socially excluded AND (Review_Ongoing):RS AND (Intervention):RT \| 2 \| \| #8 \| social exclusion AND (Review_Ongoing):RS AND (Intervention):RT \| 15 \| \| #9 \| social exclusions AND (Review_Ongoing):RS AND (Intervention):RT \| 0 \| \| #10 \| lonely AND (Review_Ongoing):RS AND (Intervention):RT \| 10 \| \| #18 \| #1 OR #2 OR #3 OR #4 OR #5 OR #6 OR #7 OR #8 OR #9 OR #10 \| 194 \| | 194 |
| Open Grey System for Information on Grey Literature in Europe  [https://easy.dans.knaw.nl/ui/advancedsearch](https://easy.dans.knaw.nl/ui/advancedsearch;jsessionid=526DDB7F427C278D2DA1D35A6666CB7F) | June 20 2022 | Advanced search: Any field: (loneliness or lonely or "social exclusion" or "social exclusions" or "socially excluded" or "social isolation" or "socially isolated") AND review | 5 hits (no systematic reviews) |
| The Campbell Collaboration  <https://www.campbellcollaboration.org/> | June 21 2022 | Resultata kan ikkje eksporterast. Sjå gjennom trefflistene:  loneliness: <https://www.campbellcollaboration.org/website-search.html?searchword=loneliness&searchphrase=all>  lonely: <https://www.campbellcollaboration.org/website-search.html?searchword=lonely&searchphrase=all&limit=20>  social isolation: <https://www.campbellcollaboration.org/website-search.html?searchword=social%20isolation&searchphrase=all&limit=20>  socially isolated: <https://www.campbellcollaboration.org/website-search.html?searchword=socially%20isolated&searchphrase=all&limit=20>  social exclusion: <https://www.campbellcollaboration.org/website-search.html?searchword=social%20exclusion&searchphrase=all>  social exclusions: <https://www.campbellcollaboration.org/website-search.html?searchword=social%20exclusions&searchphrase=all&limit=20>  socially excluded: <https://www.campbellcollaboration.org/website-search.html?searchword=socially%20excluded&searchphrase=all&limit=20> | See links to hitlists |
| SBU – Statens beredning för medicinsk och social utvärdering  <https://www.sbu.se/sv/> | June 21 2022 | The results cannot be exported to EndNote or limited to date. Look through the hitlists to find the publications from 2017 to 2022:  ensamhet: <https://www.sbu.se/sv/sok/?q=ensamhet>+ 14 hits  social isolering: <https://www.sbu.se/sv/sok/?q=social+isolering> 48 hits | See links |
| WHO Library Catalog  <https://kohahq.searo.who.int/cgi-bin/koha/opac-search.pl> | June 21 2022 | Advanced search:  Keyword: loneliness  Publication date range: 2017-2022 | 0 |
| WHO IRIS  <http://apps.who.int/iris/> | June 21 2022 | Browsing by Subject "Loneliness" | 4  (2 from 2017-2022) |
| [The Grey Literature Report in Public Health](http://www.greylit.org/) | June 21 2022 | loneliness  Publication Year: 2017-2022  lonely  Publication Year: 2017-2022  social exclusion  Publication Year: 2017-2022  social exclusions  Publication Year: 2017-2022  socially excluded  Publication Year: 2017-2022  social isolation  Publication Year: 2017-2022  social isolation  Publication Year: 2017-2022  socially isolated  Publication Year: 2017-2022 | 0  0  0  0  0  0  0  0 |
| [Mednar](https://mednar.com/mednar/desktop/en/search.html)  <https://mednar.com/> | June 21 2022 | Search: Full Record: loneliness / Title: review / From: 2017 / To: 2022  Medical  Topics: systematic review | 74 |
| [Socialstyrelsen](https://socialstyrelsen.dk/udgivelser) | June 21 2022 | Søk: ensamhet  156 results  Sort on "Datum" and look through the publications from 2017-2022:  <https://www.socialstyrelsen.se/sok/?q=ensamhet> | See link |
| [Statens Institut for Folkesundhed](https://www.sdu.dk/da/sif/rapporter) | June 21 2022 | Søk: ensomhed  55 results:  <https://www.sdu.dk/da/sif/search?q=ensomhed&l=0&ps=&c=&sc=&s=1&pst=> | See link |
| Preprints in Embase(Ovid) | September 13 2022 | Embase <1974 to 2022 September 12>  1 loneliness/ or social Isolation/ 39457  2 (loneliness or lonely or (social* adj (exclusion? or excluded or isolation or isolated))).tw,kf. 30486  3 1 or 2 50791  4 limit 3 to "reviews (maximizes specificity)" 1003  5 exp Meta-Analysis/ or "systematic review"/ or ((systematic* adj2 review*) or metaanal* or "meta anal*" or (review and ((structured or database* or systematic*) adj2 search*)) or "integrative review*" or (evidence adj2 review*)).tw,kf. 667120  6 4 or (3 and 5) 1775  7 limit 6 to yr="2017 -Current" 1199  8 limit 7 to "preprints (unpublished, non-peer reviewed)" 5 | 5 |

**LONELINESS – OVERVIEW OF OVERVIEWS**

**UPDATE SEARCH**

| **Contact person:** | Thomas Hansen |
| --- | --- |
| **Search:** | Ragnhild Agathe Tornes |
| **Duplicate control in EndNote:** | Before duplicate control: 1560  After duplicate control: 851 |

**Database:** Ovid MEDLINE(R) and Epub Ahead of Print, In-Process, In-Data-Review & Other Non-Indexed Citations, Daily and Versions <1946 to June 15, 2023>

**Date:** 19.06.23

**Number of hits:** 341

| 1 | Loneliness/ or Social Isolation/ | 21068 |
| --- | --- | --- |
| 2 | (loneliness or lonely or (social* adj (exclusion? or excluded or isolation or isolated))).tw,kf. | 27233 |
| 3 | 1 or 2 | 38741 |
| 4 | limit 3 to "reviews (maximizes specificity)" | 1009 |
| 5 | Meta-Analysis/ or Network Meta-Analysis/ or ((systematic* adj2 review*) or metaanal* or "meta anal*" or (review and ((structured or database* or systematic*) adj2 search*)) or "integrative review*" or (evidence adj2 review*)).tw,kf,bt. | 508553 |
| 6 | 4 or (3 and 5) | 1348 |
| 7 | 2023*.ed,ep,yr,dp,dt. | 947168 |
| 8 | (202206* or 202207* or 202208* or 202209* or 202210* or 202211* or 202212*).ep,ed,dt. | 1156910 |
| 9 | 7 or 8 | 1894376 |
| 10 | 6 and 9 | 341 |

**Database:** Embase <1974 to 2023 June 16> (via Ovid)

**Date:** 19.06.23

**Number of hits:** 377

| 1 | loneliness/ or social Isolation/ | 43793 |
| --- | --- | --- |
| 2 | (loneliness or lonely or (social* adj (exclusion? or excluded or isolation or isolated))).tw,kf. | 34499 |
| 3 | 1 or 2 | 56277 |
| 4 | limit 3 to "reviews (maximizes specificity)" | 1212 |
| 5 | exp Meta-Analysis/ or "systematic review"/ or ((systematic* adj2 review*) or metaanal* or "meta anal*" or (review and ((structured or database* or systematic*) adj2 search*)) or "integrative review*" or (evidence adj2 review*)).tw,kf. | 763909 |
| 6 | 4 or (3 and 5) | 2171 |
| 7 | 2023*.yr,dd,dp,dc. | 1251581 |
| 8 | (202206* or 202207* or 202208* or 202209* or 202210* or 202211* or 202212*).dd,dc. | 1318763 |
| 9 | 7 or 8 | 2526884 |
| 10 | 6 and 9 | 510 |
| 11 | limit 10 to (conference abstracts or embase) | 377 |

**Database:** APA PsycInfo <1806 to June Week 2 2023> (via Ovid)

**Date:** 19.06.23

**Number of hits:** 199

| 1 | Loneliness/ or Social Isolation/ | 14836 |
| --- | --- | --- |
| 2 | (loneliness or lonely or (social* adj (exclusion? or excluded or isolation or isolated))).tw. | 28678 |
| 3 | 1 or 2 | 32779 |
| 4 | limit 3 to "reviews (maximizes specificity)" | 961 |
| 5 | (meta analysis or "systematic review").md. or meta analysis/ or ((systematic* adj2 review*) or metaanal* or "meta anal*" or (review and ((structured or database* or systematic*) adj2 search*)) or "integrative review*" or (evidence adj2 review*)).tw. | 109297 |
| 6 | 4 or (3 and 5) | 1251 |
| 7 | 2023*.yr,dp,up. | 95909 |
| 8 | (202206* or 202207* or 202208* or 202209* or 202210* or 202211* or 202212*).up. | 106785 |
| 9 | 7 or 8 | 193943 |
| 10 | 6 and 9 | 199 |

**Database:** Sociological Abstracts (via ProQuest)

**Date:** 19.06.23

**Number of hits:** 35

| S1 | SU.EXACT("Loneliness" or "Social Isolation") | 6271 |
| --- | --- | --- |
| S2 | AB,TI(loneliness or lonely or (social* P/0 (exclusion or exclusions or excluded or isolation or isolated))) | 15,183 |
| S3 | S1 or S2 | 16,615 |
| S4 | SU.EXACT("Technology Assessment") OR SU.EXACT("Literature Reviews") or AB,TI((systematic* N/1 review*) or metaanal* or "meta anal*" or (review and ((structured or database* or systematic*) N/1 search*)) or "integrative review*" or (evidence N/1 review*)) | 19,248 |
| S5 | S3 and S4 | 284 |
| S6 | (S3 and S4) AND pd(20220616-20230619) | 35 |

**Database:** Cinahl (via Ebsco)

**Date:** 19.06.23

**Number of hits:** 157

| S8 | S7  Limiters - Exclude MEDLINE records | 157 |
| --- | --- | --- |
| S7 | S6  Limiters - Published Date: 20220601-20230631 | 170 |
| S6 | S4 or (S3 and S5) | 1,009 |
| S5 | (MH "Meta Analysis") OR (MH "Systematic Review") or TI ((systematic* N1 review*) or metaanal* or "meta anal*" or (review and ((structured or database* or systematic*) N1 search*)) or "integrative review*" or (evidence N1 review*)) OR AB ((systematic* N1 review*) or metaanal* or "meta anal*" or (review and ((structured or database* or systematic*) N1 search*)) or "integrative review*" or (evidence N1 review*)) | 253,118 |
| S4 | S3  Limiters - Clinical Queries: Review - High Specificity | 583 |
| S3 | S1 OR S2 | 23,992 |
| S2 | TI(loneliness or lonely or (social* W0 (exclusion# or excluded or isolation or isolated))) or AB(loneliness or lonely or (social* W0 (exclusion# or excluded or isolation or isolated))) | 15,263 |
| S1 | (MH "Loneliness") or (MH "Social Isolation") | 16,321 |

**Database:** Web of Science Core Collection: Science Citation Index Expanded (SCI-EXPANDED) --1987-present, Social Sciences Citation Index (SSCI) --1987-present, Arts & Humanities Citation Index (A&HCI) --1987-present, Emerging Sources Citation Index (ESCI) --2015-present

**Date:** 19.06.23

**Number of hits:** 199

| 4 | #1 and #2  Timespan: 2022-06-17 to 2023-06-19 | \| Exact search | 199 |
| --- | --- | --- | --- |
| 3 | #1 AND #2 | \| Exact search | 777 |
| 2 | TS=(("systematic*" NEAR/1 "review*") or ("review" and (("structured" or "database*" or "systematic*") NEAR/1 "search*")) or "integrative review*" or ("evidence" NEAR/1 "review*")) OR TI=("metaanal*" or "meta anal*") OR AB=("metaanal*" or "meta anal*") | \| Exact search | 562,947 |
| 1 | TS= (loneliness or lonely or (social* W0 (exclusion$ or excluded or isolation or isolated))) | \| Exact search | 23,491 |

**Database:** Cochrane Database of Systematic Reviews

Issue 6 of 12, June 2023

**Date:** 19.06.23

**Number of hits:** 3 reviews

| #1 | [mh ^Loneliness] | 247 |
| --- | --- | --- |
| #2 | (loneliness or lonely or (social* NEXT (exclusion? or excluded or isolation or isolated))):ti,ab | 2086 |
| #3 | #1 or #2 | 2105 |
| #4 | #3 in Cochrane Reviews and Cochrane Protocols | 29 |
| #5 | #4 with Cochrane Library publication date Between Jun 2022 and Jun 2023, in Cochrane Reviews, Cochrane Protocols | 3 |

**Database:** Epistemonikos

**Date:** 19.06.23

**Number of hits:** 249

Publication type: Broad Synthesis: 16

Publication type: Structured Summary: 0

Publication type: Systematic Review: 233

**Note:** Simplified search strategy due to the limited search functionality

Title/Abstract: (loneliness or lonely or "social exclusion" or "social exclusions" or "socially excluded" or "social isolation" or "socially isolated")

Publication year: 2022-2023

Added to database: From: 17-06-22 To: 19-06-23

Loneliness and social isolation – grey literature

(update search)

Effects of interventions on loneliness and social isolation

**Contact person:** Thomas Hansen

**Search:** Ragnhild Agathe Tornes

**Date:** June 19 2023

**Duplicate control in EndNot**e: Before duplicate control: 94

After duplicate control: 92

| DATABASE | DATE | SEARCH | HITS |
| --- | --- | --- | --- |
| Swemed+  <https://svemedplus.kib.ki.se/> | -- | \| Has not been updated since 2019. Nothing new here. \| Antall träffar \| \| --- \| --- \| | --- |
| [Prospero](https://www.crd.york.ac.uk/PROSPERO/)  <https://www.crd.york.ac.uk/PROSPERO/> | June 19 2023 | #1 MeSH DESCRIPTOR loneliness AND (Review_Ongoing):RS AND (Intervention):RT WHERE CD FROM 20/06/2022 TO 2023 0  #2 MeSH DESCRIPTOR social isolation AND (Review_Ongoing):RS AND (Intervention):RT WHERE CD FROM 20/06/2022 TO 2023 0  #3 loneliness AND (Review_Ongoing):RS AND (Intervention):RT 144  #4 social isolation AND (Review_Ongoing):RS AND (Intervention):RT 112  #5 socially isolated AND (Review_Ongoing):RS AND (Intervention):RT 11  #6 social exclusion AND (Review_Ongoing):RS AND (Intervention):RT 21  #7 socially excluded AND (Review_Ongoing):RS AND (Intervention):RT 3  #8 social exclusion AND (Review_Ongoing):RS AND (Intervention):RT 21  #9 social exclusions AND (Review_Ongoing):RS AND (Intervention):RT 0  #10 lonely AND (Review_Ongoing):RS AND (Intervention):RT 14  #11 #1 OR #2 OR #3 OR #4 OR #5 OR #6 OR #7 OR #8 OR #9 OR #10 231 | 57  (registrated after June 2022) |
| Open Grey System for Information on Grey Literature in Europe  [https://easy.dans.knaw.nl/ui/advancedsearch](https://easy.dans.knaw.nl/ui/advancedsearch;jsessionid=526DDB7F427C278D2DA1D35A6666CB7F) | June 19 2023 | Advanced search: Any field: (loneliness or lonely or "social exclusion" or "social exclusions" or "socially excluded" or "social isolation" or "socially isolated") AND review  5 hits | No new hits |
| The Campbell Collaboration  <https://www.campbellcollaboration.org/> | June 19 2023 | The results cannot be exported to EndNote or limited to date. Look through the hitlists to find publications published after June 20 2022:  loneliness: <https://www.campbellcollaboration.org/website-search.html?searchword=loneliness&searchphrase=all>  lonely: <https://www.campbellcollaboration.org/website-search.html?searchword=lonely&searchphrase=all&limit=20>  social isolation: <https://www.campbellcollaboration.org/website-search.html?searchword=social%20isolation&searchphrase=all&limit=20>  socially isolated: <https://www.campbellcollaboration.org/website-search.html?searchword=socially%20isolated&searchphrase=all&limit=20>  social exclusion: <https://www.campbellcollaboration.org/website-search.html?searchword=social%20exclusion&searchphrase=all>  social exclusions: <https://www.campbellcollaboration.org/website-search.html?searchword=social%20exclusions&searchphrase=all&limit=20>  socially excluded: <https://www.campbellcollaboration.org/website-search.html?searchword=socially%20excluded&searchphrase=all&limit=20> | See links to hitlists |
| SBU – Statens beredning för medicinsk och social utvärdering  <https://www.sbu.se/sv/> | June 19 2023 | The results cannot be exported to EndNote. Look through the hitlists. Choose "Sortera efter: Datum" to get the newest publication first.  ensamhet: <https://www.sbu.se/sv/sok/?q=ensamhet>  social isolering: <https://www.sbu.se/sv/sok/?q=social+isolering> | See links to hitlists |
| WHO Library Catalog  <https://kohahq.searo.who.int/cgi-bin/koha/opac-search.pl> | June 19 2023 | Advanced search:  Keyword: loneliness  Publication date range: 2022-2023 | 0 |
| WHO IRIS  <http://apps.who.int/iris/> | June 19 2023 | Browsing by Subject "Loneliness" | 4  (0 from 2022-2023) |
| [The Grey Literature Report in Public Health](http://www.greylit.org/) | June 19 2023 | No longer valid web adress | -- |
| [Mednar](https://mednar.com/mednar/desktop/en/search.html)  <https://mednar.com/> | June 19 2023 | Advanced search: Full Record: loneliness / Title: review / From: 2017 / To: 2022  Medical  Topics: systematic review | 24 |
| Preprints in Embase(Ovid) | June 19 2023 | Embase <1974 to 2023 June 16>  1 loneliness/ or social Isolation/ 43793  2 (loneliness or lonely or (social* adj (exclusion? or excluded or isolation or isolated))).tw,kf. 34499  3 1 or 2 56277  4 limit 3 to "reviews (maximizes specificity)" 1212  5 exp Meta-Analysis/ or "systematic review"/ or ((systematic* adj2 review*) or metaanal* or "meta anal*" or (review and ((structured or database* or systematic*) adj2 search*)) or "integrative review*" or (evidence adj2 review*)).tw,kf. 763909  6 4 or (3 and 5) 2171  7 limit 6 to yr="2017 -Current" 573  8 limit 7 to "preprints (unpublished, non-peer reviewed)" 11 | 11 |
| [Socialstyrelsen](https://socialstyrelsen.dk/udgivelser) | June 19 2023 | Søk: ensamhet  156 hits  "Sortera på: Datum" and browse the publications from 2022-2023:  <https://www.socialstyrelsen.se/sok/?q=ensamhet> | See link |
| [Statens Institut for Folkesundhed](https://www.sdu.dk/da/sif/rapporter) | June 19 2023 | Søk: ensomhed  54 hits:  <https://www.sdu.dk/da/sif/search?q=ensomhed&l=0&ps=&c=&sc=&s=1&pst=> | See link |

**Appendix 4**

**Table**. Excluded studies and reasons for exclusions (n=141)

| **Author data** | **Reason for exclusion** |
| --- | --- |
| Abdi 2017 | No SR of RCTs |
| Alexandra 2018 | No SR of RCTs |
| Antunes 2019 | No SR of RCTs |
| Ashaari 2021 | Not in English or Scandinavian language |
| Astell-Burt 2022 | No SR of RCTs |
| Austin 2021 | Wrong or no outcomes of interest |
| Bagnall 2023 | Ongoing work |
| Baker 2018 | No SR of RCTs |
| Banbury 2018 | Wrong or no outcomes of interest |
| Bauer 2021 | Wrong or no outcomes of interest |
| Berkanish 2022 | No SR |
| Bermeja 2018 | Not in English or Scandinavian language |
| Bessaha 2020 | No SR |
| Bochicchio 2022 | No SR of RCTs |
| Boldi 2021 | Wrong or no outcomes of interest |
| Boldig 2021 | Wrong or no outcomes of interest |
| Boldt 2021 | Wrong or no outcomes of interest |
| Bong 2018 | No SR |
| Bordini 2023 | Ongoing work |
| Bourne 2021 | Wrong or no outcomes of interest |
| Brimelow 2017 | No SR |
| Brooks 2018 | No SR of RCTs |
| Bursky 2021 | No SR |
| Butz 2023 | Ongoing work |
| Cadth Medical Services 2023 | Ongoing work |
| Cai 2023 | Ongoing work |
| Casanova 2021 | No SR |
| Chang 2021 | Wrong or no outcomes of interest |
| Chipps 2017 | No SR |
| Clements 2019 | No SR of RCTs |
| Coll-Planas 2017 | Wrong or no outcomes of interest |
| Crowe 2022 | No SR |
| DiPerna 2023 | No SR of RCTs |
| Domenicucci 2022 | Wrong or no outcomes of interest |
| Donaldson 2022 | Only 1 RCT, covered by other reviews (28, 29) |
| Dworschak 2022 | Wrong or no outcomes of interest |
| Eddy 2023 | Ongoing work |
| Egan 2023 | Ongoing work |
| Elhag 2023 | Ongoing work |
| Ellis 2021 | No SR of RCTs |
| En 2022 | Wrong or no outcomes of interest |
| European Observatory on Health 2019 | No SR of RCTs |
| Fischer 2023 | No SR |
| Foettinger 2022 | No SR of RCTs |
| Forgeron 2018 | Wrong or no outcomes of interest |
| G 2022 | Wrong or no outcomes of interest |
| Galustyants 2022 | Discontinued and unpublished |
| Garcia 2022 | No SR |
| Gerrity 2019 | Wrong or no outcomes of interest |
| Gilmour 2020 | No SR of RCTs |
| Haas 2023 | Ongoing work |
| Hagani 2023 | Ongoing work |
| Hall 2019 | No SR |
| Handley 2021 | No SR of RCTs |
| Hards 2022 | No SR of RCTs |
| Hollands 2023 | Ongoing work |
| Holttum 2018 | No SR |
| Husted 2023 | Ongoing work |
| Ibarra 2020 | No SR of RCTs |
| Ibrahim 2021 | No SR |
| Ilgaz 2019 | Wrong or no outcomes of interest |
| Ingram 2020 | No SR of RCTs |
| Isabet 2021 | No SR |
| Jagroep 2022 | Wrong or no outcomes of interest |
| Jain 2020 | Wrong or no outcomes of interest |
| Jenni 2019 | Wrong or no outcomes of interest |
| Johnstone 2021 | Wrong or no outcomes of interest |
| Kiely 2023 | Ongoing work |
| Koller 2021 | No SR of RCTs |
| Kuru Alici 2020 | No SR of RCTs |
| Kusumota 2022 | Not in English or Scandinavian language |
| Laermans 2023 | Ongoing work |
| Lane 2022 | No SR of RCTs |
| Larsson 2020 | No SR |
| Latikka 2021 | No SR |
| Li 2022 | No SR |
| Lindsay 2018 | Wrong or no outcomes of interest |
| Littlewood 2022 | No SR |
| Lobbia 2019 | Wrong or no outcomes of interest |
| Manjunath 2021 | No SR |
| Mann 2017 | No SR |
| Marciano 2021 | No SR of RCTs |
| Marfell 2023 | Ongoing work |
| Mathewson 2022 | Discontinued and unpublished |
| Miake-Lye 2023 | Ongoing work |
| Mikkelsen 2019 | No SR of RCTs |
| Moore 2018 | Wrong or no outcomes of interest |
| Moriarty 2017 | No SR |
| Morrish 2023 | Ongoing work |
| Murray 2022 | Wrong or no outcomes of interest |
| Nnabuko 2018 | Wrong or no outcomes of interest |
| Noone 2020 | No SR of RCTs |
| Pallavicini 2022 | No SR of RCTs |
| Pan 2021 | No SR |
| Paquet 2023 | No SR of RCTs |
| Pathrose 2021 | No SR of RCTs |
| Pearce 2021 | No SR |
| Percival 2022 | No SR of RCTs |
| Peters 2021 | No SR of RCTs |
| Pool 2017 | No SR |
| Portz 2017 | Wrong or no outcomes of interest |
| Pu 2019 | Wrong or no outcomes of interest |
| Puyat 2020 | Wrong or no outcomes of interest |
| Quinn 2023 | Ongoing work |
| Reinhardt 2021 | No SR |
| Sen 2022 | No SR of RCTs |
| Shakya 2022 | No SR |
| Shishehgar 2019 | No SR |
| Song 2019 | No SR |
| Stojkov 2023 | Ongoing work |
| Strudwick 2021 | No SR of RCTs |
| Swinkels 2023 | Ongoing work |
| Tcymbal 2022 | No SR of RCTs |
| Thompson 2022 | No SR of RCTs |
| Timko Olson 2020 | Wrong or no outcomes of interest |
| Todd 2022 | No SR |
| Tricco 2022 | No SR of RCTs |
| Tshikaya 2023 | Ongoing work |
| Ulusoy 2023 | No SR of RCTs |
| Van der Meulen 2021 | No SR of RCTs |
| Vasquez 2023 | Ongoing work |
| Veazie 2019 | Wrong or no outcomes of interest |
| Velloze 2022 | No SR |
| Victor 2018 | No SR |
| Vidovic 2021 | No SR |
| Villalonga-Olives 2022 | No SR |
| Wang 2022 | Wrong or no outcomes of interest |
| Webber 2017 | No SR |
| Williams 2022 | No SR of RCTs |
| Wilson 2018 | No SR |
| Wolters 2023 | Ongoing work |
| Yu 2023 | No SR of RCTs |
| Zhang, D. 2021 | No SR of RCTs |
| Zhang, L. 2022 | No SR of RCTs |
| Zhang, K.X. 2023 | Wrong or no outcomes of interest |
| Zhong 2020 | No SR of RCTs |
| Zollick 2021 | Not in English or Scandinavian language |

Note: SR = Systematic review. “No SR” = Not meeting our criteria for a SR (clear PICO, risk of bias assessments, comprehensive search strategy). “No SR of RCTs” = The SR do not include RCTs. “Wong or no outcomes of interest” = The SR do not include RCTs on loneliness and/or social isolation. “Discontinued and unpublished” = The authors have notified us (via email) that their work on the SR was discontinued before completion. Record not found = authors were contacted three times, without response.

**Table**. Records not found (n= 14)

| **Author** | **Title** | **Intervention type** |
| --- | --- | --- |
| Bellido | Effect of physical exercise programs with digital health interventions in mental health in older adults: systematic review and meta-analysis | Exercise |
| Gonzalez-Moral | What is the effectiveness of social prescribing interventions in older adults for physical and mental well being? A systematic review | Social prescribing |
| Hewson | The effects of adapted mind-body exercises in improving physical function, wellbeing and quality of life for older people: a systematic review | Exercise |
| Huang | A systematic review of studies investigating the effectiveness of psychological interventions in reducing loneliness in individuals with psychiatric disorders | Psychological |
| Jong | Physical activity has the potential to reduce loneliness in older adults | Physical activity |
| Li | Effects of an online mindfulness intervention on physiology and psychology health in adults during COVID-19: a systematic review and meta-analysis of randomized controlled trials | Mindfulness |
| McConnell | A meta-analysis on the effectiveness of animal-assisted interventions on the psychological outcomes of patients undergoing psychological therapeutic interventions | Animal-assisted |
| Qi | Effects of information and communication technology interventions on alleviating social disconnection in older adults: a systematic review and meta-analysis of randomized controlled trials | ICT |
| Raciti | Interventions targeting loneliness and social isolation among long-term care residents: A systematic review | Diverse |
| Staras | Effectiveness of UK-based Social Prescribing Initiatives Designed To Increase Social Connection, Reduce Loneliness, and Benefit Wellbeing: A Systematic Review | Social prescribing |
| Takahashi | Communication robot for improve the health and well-being of elderly people with dementia: A systematic review and meta-analysis | Communication robot |
| Tan | Effectiveness of health promotion interventions on loneliness among older adults: A systematic review | Health promotion |
| Vella | A rapid systematic review of social interventions to support young people with co-present social and mental health problems | Social |
| Winkler | A systematic review of studies investigating the effectiveness of psychological interventions in reducing loneliness in individuals with psychiatric disorders | Psychological |

**Appendix 5.** Critical appraisal of included systematic reviews: AMSTAR II consensus results

AMSTAR Domain questions

| 1. PICO | Did the research questions and inclusion criteria for the review include the components of PICO? (yes/no) | | |  |  |
| --- | --- | --- | --- | --- | --- |
| 2. Protocol | Was a complete protocol written? (yes/partial yes/no) | | |  |  |
| 3. Study design | Did the authors explain their selection of the study designs for inclusion in the review? (yes/no) | | |  |  |
| 4. Search strategy | Comprehensive search strategy? (yes/partial yes/no) | | |  |  |
| 5. Study selection | In duplicate? (yes/no) | | |  |  |
| 6. Data extraction | In duplicate? (yes/no) | | |  |  |
| 7. Excluded studies | List of excluded studies and justification? (yes/partial yes/no) | | |  |  |
| 8. Included studies | Included studies described in detail? (yes/partial yes/no) | | |  |  |
| 9. RoB assessment | Risk of bias assessed? (yes/partial yes/no) | | |  |  |
| 10. Funding sources | Reported? (yes/no) | | |  |  |
| 11. Meta-analysis | Appropriate methods used? (yes/no/no meta-analysis) | | |  |  |
| 12. Impact of risk of bias | Was impact on results assessed? (yes/no/no meta-analysis) | | |  |  |
| 13. Discussing risk of bias | Was potential effects of bias discussed? (yes/no/no meta-analysis) |  | Methodological requirements met | |  |
| 14. Heterogeneity | Discussion of heterogeneity? (yes/no) |  | Methodological requirements partly met | | |
| 15. Publication bias | Investigated? (yes/no/no meta-analysis) |  | Methodological requirements not met | | |
| 16. Conflict of interest | Did the review authors report any? (yes/partial yes/no) |  | Not applicable (no meta-analysis) | |  |

The display is adapted from Uphoff et al. (77). AMSTAR2 includes 16 domains with an overall rating based on weaknesses in critical domains (C = critical) (40). The domains included in the tool relate to PICO, protocol (C), study design, search strategy (C), study selection, data extraction, justification for excluded studies (C), description of included studies, risk of bias (C), sources of funding, meta-analysis (C), assessing impact of bias, discussing impact of bias (C), heterogeneity, publication bias (C), and conflicts of interest. As shown, all of the SRs had clearly defined PICO components, as required by the eligibility criteria (domain #1). Most SRs had conducted comprehensive search strategies^[[1]](#footnote-1)^ (#4) and performed study selection and data extraction in duplicate (#5 and #6). All reviews with meta-analysis, except one, used an appropriate method for conducting the meta-analysis (#11). However, almost half of SRs did not prepare a protocol (#2) or justify their choice of study selection (#3). Similarly, nearly all SRs failed to provide a list of excluded studies and justification for exclusion (#7). Although almost all SRs described the included studies in detail (#8) and conducted risk of bias assessments (#9), most only partially completed these steps. Moreover, while most SRs assessed the impact of risk of bias on results (#12), one-third failed to discuss its effect on the findings (#13). Most SRs with meta-analysis, but few with narrative synthesis, properly discussed heterogeneity in the results (#14). Over half of the SRs, and all of those which had narrative synthesis, did not include a publication bias assessment (#15). Only nine SRs, none of which had narrative synthesis, investigated publication bias (#15). While all reviews provided a conflict-of-interest statement (#16), most failed to report sources of funding for the included studies (#10).

**Appendix 6**. Assessment of overlap

Table. Number of RCTs (n) overlapped among reviews

|  | **1** | **2** | **3** | **4** | **5** | **6** | **7** | **8** | **9** | **10** | **11** | **12** | **13** | **14** | **15** | **16** | **17** | **18** | **19** | **20** | **21** | **22** | **23** | **24** | **25** | **26** | **27** | **28** | **29** |
| --- | --- | --- | --- | --- | --- | --- | --- | --- | --- | --- | --- | --- | --- | --- | --- | --- | --- | --- | --- | --- | --- | --- | --- | --- | --- | --- | --- | --- | --- |
| 1.Abbott 2019 (2) | 2 | 0 | 1 | 2 | 0 | 0 | 0 | 0 | 1 | 0 | 0 | 0 | 2 | 0 | 0 | 0 | 0 | 0 | 1 | 1 | 0 | 0 | 0 | 0 | 0 | 2 | 0 | 1 | 0 |
| 2.Barnett 2020 (23) | 0 | 18 | 0 | 2 | 0 | 0 | 0 | 0 | 0 | 0 | 0 | 2 | 0 | 0 | 0 | 7 | 0 | 0 | 0 | 0 | 0 | 0 | 0 | 0 | 0 | 1 | 0 | 1 | 0 |
| 3.Choi 2021 (3) | 1 | 0 | 4 | 2 | 0 | 0 | 0 | 1 | 1 | 0 | 0 | 1 | 2 | 0 | 0 | 0 | 0 | 0 | 0 | 0 | 2 | 0 | 0 | 0 | 0 | 1 | 0 | 1 | 0 |
| 4.Christ. 2021 (54) | 2 | 2 | 2 | 55 | 2 | 3 | 0 | 2 | 3 | 0 | 0 | 13 | 17 | 2 | 0 | 1 | 1 | 1 | 1 | 2 | 2 | 2 | 0 | 3 | 4 | 14 | 1 | 17 | 0 |
| 5.Eccles 2021 (25) | 0 | 0 | 0 | 2 | 26 | 1 | 0 | 0 | 0 | 0 | 0 | 4 | 0 | 0 | 0 | 1 | 0 | 2 | 0 | 0 | 0 | 0 | 0 | 0 | 0 | 0 | 0 | 1 | 0 |
| 6. Ellard 2022 (16) | 0 | 0 | 0 | 3 | 1 |  | 0 | 0 | 0 | 0 | 0 | 2 | 0 | 0 | 0 | 0 | 0 | 1 | 0 | 0 | 0 | 0 | 0 | 2 | 0 | 3 | 0 | 2 | 2 |
| 7.Forsman 2018 (6) | 0 | 0 | 0 | 0 | 0 | 0 | 7 | 3 | 0 | 0 | 0 | 0 | 3 | 2 | 1 | 0 | 0 | 0 | 0 | 0 | 0 | 0 | 1 | 0 | 3 | 5 | 1 | 1 | 0 |
| 8.Fu 2022 (13) | 0 | 0 | 1 | 2 | 0 | 0 | 3 | 13 | 0 | 2 | 0 | 4 | 7 | 3 | 0 | 0 | 1 | 0 | 0 | 0 | 1 | 0 | 1 | 0 | 4 | 3 | 1 | 3 | 0 |
| 9.Gardiner 2018 (6) | 1 | 0 | 1 | 3 | 0 | 0 | 0 | 0 | 6 | 0 | 0 | 1 | 5 | 0 | 0 | 0 | 0 | 0 | 1 | 0 | 0 | 1 | 0 | 1 | 2 | 3 | 0 | 1 | 0 |
| 10.Hao 2023 (4) | 0 | 0 | 0 | 0 | 0 | 0 | 0 | 2 | 0 |  | 0 | 0 | 0 | 0 | 0 | 0 | 0 | 0 | 0 | 0 | 0 | 0 | 0 | 0 | 0 | 0 | 0 | 0 | 0 |
| 11.Heins 2021 (3) | 0 | 0 | 0 | 0 | 0 | 0 | 0 | 0 | 0 | 0 | 3 | 0 | 0 | 0 | 0 | 0 | 0 | 0 | 0 | 0 | 0 | 0 | 0 | 0 | 0 | 0 | 0 | 0 | 0 |
| 12.Hickin 2021 (31) | 0 | 2 | 1 | 13 | 4 | 2 | 0 | 4 | 1 | 0 | 0 | 31 | 5 | 0 | 0 | 0 | 2 | 4 | 0 | 1 | 1 | 0 | 0 | 3 | 2 | 5 | 0 | 13 | 0 |
| 13.Hoang 2022 (70) | 2 | 0 | 2 | 17 | 0 | 0 | 3 | 7 | 5 | 0 | 0 | 5 |  | 3 | 0 | 1 | 1 | 0 | 2 | 4 | 4 | 2 | 3 | 0 | 11 | 18 | 2 | 10 | 0 |
| 14.Jin 2021 (6) | 0 | 0 | 0 | 2 | 0 | 0 | 2 | 3 | 0 | 0 | 0 | 0 | 3 | 6 | 0 | 0 | 0 | 0 | 1 | 0 | 0 | 0 | 0 | 0 | 1 | 2 | 1 | 1 | 0 |
| 15.Li 2018 (4) | 0 | 0 | 0 | 0 | 0 | 0 | 1 | 0 | 0 | 0 | 0 | 0 | 0 | 0 | 4 | 0 | 0 | 0 | 0 | 0 | 0 | 0 | 0 | 0 | 0 | 2 | 0 | 0 | 0 |
| 16.Ma 2020 (30) | 0 | 7 | 0 | 1 | 1 | 0 | 0 | 0 | 0 | 0 | 0 | 0 | 1 | 0 | 0 | 29 | 0 | 0 | 0 | 0 | 0 | 0 | 0 | 0 | 1 | 1 | 0 | 0 | 0 |
| 17.McElfresh 2021 (7) | 0 | 0 | 0 | 1 | 0 | 0 | 0 | 1 | 0 | 0 | 0 | 2 | 1 | 0 | 0 | 0 | 8 | 0 | 0 | 0 | 0 | 0 | 0 | 1 | 0 | 0 | 0 | 2 | 0 |
| 18.Osborn 2021 (5) | 0 | 0 | 0 | 1 | 2 | 1 | 0 | 0 | 0 | 0 | 0 | 4 | 0 | 0 | 0 | 0 | 0 | 5 | 0 | 0 | 0 | 0 | 0 | 1 | 0 | 1 | 0 | 2 | 0 |
| 19.Poscia 2018 (2) | 1 | 0 | 0 | 1 | 0 | 0 | 0 | 0 | 1 | 0 | 0 | 0 | 2 | 1 | 0 | 0 | 0 | 0 | 3 | 1 | 0 | 0 | 0 | 0 | 1 | 2 | 0 | 1 | 0 |
| 20.Quan 2020 (5) | 1 | 0 | 0 | 2 | 0 | 0 | 0 | 0 | 0 | 0 | 0 | 1 | 4 | 0 | 0 | 0 | 0 | 0 | 1 | 5 | 0 | 0 | 0 | 0 | 0 | 2 | 0 | 2 | 0 |
| 21.Shah 2021 (5) | 0 | 0 | 2 | 2 | 0 | 0 | 0 | 1 | 0 | 0 | 0 | 1 | 4 | 0 | 0 | 0 | 0 | 0 | 0 | 0 | 4 | 0 | 0 | 0 | 0 | 1 | 1 | 1 | 0 |
| 22.Shvedko 2018 (7) | 0 | 0 | 0 | 2 | 0 | 0 | 0 | 0 | 1 | 0 | 0 | 0 | 2 | 0 | 0 | 0 | 0 | 0 | 0 | 0 | 0 | 7 | 0 | 0 | 2 | 4 | 0 | 1 | 0 |
| 23.Siette 2017 (5) | 0 | 0 | 0 | 0 | 0 | 0 | 1 | 1 | 0 | 0 | 0 | 0 | 3 | 0 | 0 | 0 | 0 | 0 | 0 | 0 | 0 | 0 | 5 | 0 | 1 | 1 | 0 | 0 | 0 |
| 24.Teoh 2021 (8) | 0 | 0 | 0 | 3 | 0 | 2 | 0 | 0 | 1 | 0 | 0 | 3 | 0 | 0 | 0 | 0 | 1 | 1 | 0 | 0 | 0 | 0 | 0 | 9 | 0 | 2 | 0 | 3 | 0 |
| 25.Tong 2021 (24) | 0 | 0 | 0 | 4 | 0 | 0 | 3 | 4 | 2 | 0 | 0 | 2 | 11 | 1 | 0 | 1 | 0 | 0 | 1 | 0 | 0 | 2 | 1 | 0 | 23 | 8 | 1 | 3 | 0 |
| 26.Williams 2021 (45) | 2 | 1 | 2 | 14 | 0 | 3 | 5 | 3 | 3 | 0 | 0 | 5 | 18 | 2 | 2 | 1 | 0 | 1 | 2 | 2 | 1 | 4 | 1 | 2 | 8 | 41 | 2 | 13 | 0 |
| 27.Wiwat. 2021 (4) | 0 | 0 | 0 | 1 | 0 | 0 | 1 | 1 | 0 | 0 | 0 | 0 | 2 | 1 | 0 | 0 | 0 | 0 | 0 | 0 | 1 | 0 | 0 | 0 | 1 | 2 | 4 | 1 | 0 |
| 28.Zagic 2021 (58) | 1 | 1 | 1 | 17 | 1 | 2 | 1 | 3 | 1 | 0 | 0 | 13 | 10 | 1 | 0 | 0 | 2 | 2 | 1 | 2 | 1 | 1 | 0 | 3 | 3 | 13 | 1 | 33 | 0 |
| 29.Zhang 2023 (8) | 0 | 0 | 0 | 0 | 0 | 2 | 0 | 0 | 0 | 0 | 0 | 0 | 0 | 0 | 0 | 0 | 0 | 0 | 0 | 0 | 0 | 0 | 0 | 0 | 0 | 0 | 0 | 0 |  |

Table. Citations matrix with systematic reviews (coloums) and unique primary studies (rows). Check marks (√) indicate when a primary study is included in a review.

|  | 1. Abbott 2019 (n=2) | 2. Barnett 2020 (n=23) | 3. Choi 2021 (n=3) | 4. Christians. 2021 (n=54) | 5. Eccles 2021 (n=25) | 6. Ellard 2022 (n=16) | 7. Forsman 2018 (n=6) | 8. Fu 2022 (n=13) | 9. Gardiner 2018 (n=6) | 10. Hao 2023 (n=4) | 11. Heins 2021 (n=3) | 12. Hickin 2021 (n=31) | 13. Hoang 202 (n=70) | 14. Jin 2021 (n=6) | 15. Li 2018 (n=4) | 16. Ma 2020 (n=30) | 17. McElfresh 2021 (n=7) | 18. Osborn 2021 (n=5) | 19. Poscia 2018 (n=2) | 20. Quan 2020 (n=5) | 21. 8.Shah 2021 (n=5) | 22.Shvedko 2018 (n=7) | 23.Siette 2017 (n=5) | 24.Teoh 2021 (n=8) | 25.Tong 2021 (n=24) | 26.Williams 2021 (n=45) | 27.Wiwat. 2021 (n=4) | 28.Zagic 2021 (n=58) | 29. Zhang 2023 (n=8) | # Times included |
| --- | --- | --- | --- | --- | --- | --- | --- | --- | --- | --- | --- | --- | --- | --- | --- | --- | --- | --- | --- | --- | --- | --- | --- | --- | --- | --- | --- | --- | --- | --- |
| Abbott 1985 |  |  |  | **√** |  |  |  |  |  |  |  |  |  |  |  |  |  |  |  |  |  |  |  |  |  |  |  |  |  | 1 |
| Aberg-Wistedt 1995 |  |  |  |  |  |  |  |  |  |  |  |  |  |  |  | **√** |  |  |  |  |  |  |  |  |  |  |  |  |  | 1 |
| Adams 1988 |  |  |  | **√** |  |  |  |  |  |  |  |  |  |  |  |  |  |  |  |  |  |  |  |  |  |  |  |  |  | 1 |
| Alaviani 2015 |  |  |  | **√** |  |  |  |  |  |  |  | **√** | **√** |  |  |  |  |  |  |  |  |  |  |  |  |  |  |  |  | 3 |
| Ammerman 2013 |  |  |  |  |  |  |  |  |  |  |  |  |  |  |  | **√** |  |  |  |  |  |  |  |  |  |  |  |  |  | 2 |
| Andersson 1985 |  |  |  | **√** |  |  |  |  |  |  |  |  | **√** |  |  |  |  |  |  |  |  |  |  |  |  | **√** |  |  |  | 3 |
| Aspy 2017 |  |  |  |  |  |  |  |  |  |  |  |  |  |  |  |  |  |  |  |  |  |  |  |  |  |  |  | **√** |  | 1 |
| Atkinson 1996 |  |  |  |  |  |  |  |  |  |  |  |  |  |  |  | **√** |  |  |  |  |  |  |  |  |  |  |  |  |  | 1 |
| Baez 2017 |  |  |  |  |  |  |  |  |  |  |  |  | **√** |  |  |  |  |  |  |  |  |  |  |  |  |  |  |  |  | 1 |
| Banks 2002 |  |  |  | **√** |  |  |  |  |  |  |  |  | **√** |  |  |  |  |  |  |  |  |  |  |  |  |  |  |  |  | 2 |
| Banks 2005 |  |  |  |  |  |  |  |  | **√** |  |  |  | **√** |  |  |  |  |  |  |  |  |  |  |  |  |  |  |  |  | 2 |
| Banks 2008 | **√** |  | **√** | **√** |  |  |  |  | **√** |  |  |  | **√** |  |  |  |  |  |  |  |  |  |  |  |  | **√** |  |  |  | 6 |
| Barber 2011 |  |  |  |  |  | **√** |  |  |  |  |  |  |  |  |  |  |  |  |  |  |  |  |  |  |  |  |  |  |  | 1 |
| Bartlett 2019 |  |  |  |  |  |  |  |  |  |  |  | **√** |  |  |  |  |  |  |  |  |  |  |  |  |  |  |  |  |  | 1 |
| Beidel 2000 |  |  |  |  | **√** |  |  |  |  |  |  |  |  |  |  |  |  |  |  |  |  |  |  |  |  |  |  |  |  | 1 |
| Besse 2016 |  |  |  |  |  | **√** |  |  |  |  |  |  |  |  |  |  |  |  |  |  |  |  |  |  |  |  |  |  |  | 1 |
| Bickmore 2005 |  |  |  |  |  |  |  |  |  |  |  |  | **√** |  |  |  |  |  |  |  |  |  |  |  |  | **√** |  |  |  | 2 |
| Björkman 2002 |  |  |  |  |  |  |  |  |  |  |  |  |  |  |  | **√** |  |  |  |  |  |  |  |  |  |  |  |  |  | 1 |
| Black 2014 |  |  |  |  |  |  |  |  |  |  |  |  |  |  |  |  |  |  |  |  |  |  |  |  | **√** |  |  |  |  | 1 |
| Boevink 2016 |  | **√** |  | **√** |  |  |  |  |  |  |  |  |  |  |  | **√** |  |  |  |  |  |  |  |  |  |  |  |  |  | 3 |
| Bond 2010 |  |  |  |  |  |  |  |  |  |  |  |  | **√** |  |  |  |  |  |  |  |  |  |  |  |  |  |  |  |  | 1 |
| Borji 2018 |  |  |  |  |  |  |  |  |  |  |  |  |  |  |  |  |  |  |  |  |  |  |  |  |  |  |  | **√** |  | 1 |
| Borji 2020 |  |  |  | **√** |  |  |  |  |  |  |  |  |  |  |  |  |  |  |  |  |  |  |  |  |  |  |  |  |  | 1 |
| Bouwman 2017 |  |  |  | **√** |  |  |  |  |  |  |  |  |  |  |  |  |  |  |  |  |  |  |  |  |  | **√** | **√** | **√** |  | 4 |
| Brennan 1991 |  |  |  |  |  |  |  |  |  |  |  |  |  |  |  |  |  |  |  |  |  |  |  |  | **√** |  |  |  |  | 1 |
| Brog 2022 |  |  |  |  |  |  |  |  |  | **√** |  |  |  |  |  |  |  |  |  |  |  |  |  |  |  |  |  |  |  | 1 |
| Bruehlman-Senec. 2020 |  |  |  |  |  | **√** |  |  |  |  |  | **√** |  |  |  |  |  | **√** |  |  |  |  |  |  |  |  |  |  |  | 3 |
| Buckle 2015 |  |  |  | **√** |  |  |  |  |  |  |  |  |  |  |  |  |  |  |  |  |  |  |  |  |  |  |  |  |  | 1 |
| Bøen 2012 |  |  |  |  |  |  |  |  |  |  |  |  | **√** |  |  | **√** |  |  |  |  |  |  |  |  | **√** |  |  |  |  | 3 |
| Cacioppo 2015 |  |  |  | **√** |  |  |  |  |  |  |  | **√** |  |  |  |  |  |  |  |  |  |  |  |  |  |  |  |  |  | 2 |
| Caputi 2020 |  |  |  |  |  |  |  |  |  |  |  | **√** |  |  |  |  |  |  |  |  |  |  |  |  |  |  |  |  |  | 1 |
| Castelein 2008 |  | **√** |  |  |  |  |  |  |  |  |  |  |  |  |  | **√** |  |  |  |  |  |  |  |  |  |  |  |  |  | 2 |
| Chan 2017 |  |  |  | **√** |  |  |  |  |  |  |  |  | **√** |  |  |  |  |  |  |  |  | **√** |  |  | **√** | **√** |  | **√** |  | 6 |
| Charlesworth 2008 |  |  |  |  |  |  |  |  |  |  |  |  | **√** |  |  |  |  |  |  |  |  |  | **√** |  |  |  |  |  |  | 2 |
| Chiang 2009 |  |  |  |  |  |  |  |  |  |  |  |  | **√** |  |  |  |  |  |  |  |  |  |  |  |  |  |  |  |  | 1 |
| Chiang 2010 |  |  |  | **√** |  |  |  |  |  |  |  | **√** |  |  |  |  |  |  |  | **√** |  |  |  |  |  | **√** |  | **√** |  | 5 |
| Choi 2020 |  |  |  |  |  |  |  | **√** |  |  |  | **√** |  |  |  |  |  |  |  |  |  |  |  |  |  |  |  | **√** |  | 3 |
| Chow 2019 |  |  |  |  |  |  |  |  |  |  |  |  | **√** |  |  |  |  |  |  |  |  |  |  |  |  |  |  |  |  | 1 |
| Christian & D'auria 2006 |  |  |  |  | **√** |  |  |  |  |  |  |  |  |  |  |  |  |  |  |  |  |  |  |  |  |  |  |  |  | 1 |
| Chu 2019 |  |  |  | **√** |  |  |  |  |  |  |  |  |  |  |  |  |  |  |  |  |  |  |  |  |  |  |  |  |  | 1 |
| Cleary 2015 |  |  |  |  |  |  |  |  |  |  |  |  |  |  |  |  | **√** |  |  |  |  |  |  |  |  |  |  |  |  | 1 |
| Cohen-Mansfield 2018 |  |  |  | **√** |  |  |  |  |  |  |  | **√** | **√** |  |  |  |  |  |  |  |  |  |  |  |  | **√** |  | **√** |  | 5 |
| Cole 1995 |  |  |  |  |  |  |  |  |  |  |  |  |  |  |  | **√** |  |  |  |  |  |  |  |  |  |  |  |  |  | 1 |
| Coleman 2005 |  |  |  |  |  |  |  |  |  |  |  |  |  |  |  |  | **√** |  |  |  |  |  |  |  |  |  |  |  |  | 1 |
| Conoley 1985 |  | **√** |  |  |  | **√** |  |  |  |  |  |  |  |  |  | **√** |  |  |  |  |  |  |  |  |  | **√** |  |  |  | 4 |
| Conoley 1998 |  |  |  |  |  |  |  |  |  |  |  |  |  |  |  |  |  |  |  |  |  |  |  |  |  |  |  | **√** |  | 1 |
| Constantino 1988 |  |  |  |  |  |  |  |  |  |  |  |  |  |  |  |  |  |  |  |  |  |  |  |  | **√** |  |  |  |  | 1 |
| Cox 2007 |  |  |  |  |  |  |  |  |  |  |  |  | **√** |  |  |  |  |  |  |  |  |  |  |  |  |  |  |  |  | 1 |
| Craig 2016 |  |  |  |  | **√** |  |  |  |  |  |  |  |  |  |  |  |  |  |  |  |  |  |  |  |  |  |  |  |  | 1 |
| Craig 2018 |  |  |  |  | **√** |  |  |  |  |  |  |  |  |  |  |  |  |  |  |  |  |  |  |  |  |  |  |  |  | 1 |
| Cresswell 2012 |  |  |  | **√** |  |  |  |  | **√** |  |  | **√** |  |  |  |  |  |  |  |  |  |  |  | **√** |  | **√** |  | **√** |  | 5 |
| Cross 2018 |  |  |  |  | **√** |  |  |  |  |  |  |  |  |  |  |  |  |  |  |  |  |  |  |  |  |  |  |  |  | 1 |
| Czaja 2017 |  |  |  |  |  |  |  |  |  |  |  |  |  |  |  |  |  |  |  |  |  |  |  |  | **√** |  |  |  |  | 1 |
| Czaja 2018 |  |  | **√** |  |  |  |  |  |  |  |  |  | **√** |  |  |  |  |  |  |  | **√** |  |  |  |  | **√** |  |  |  | 4 |
| Dammeyer 2004 |  |  |  | **√** |  |  |  |  |  |  |  |  |  |  |  |  |  |  |  |  |  |  |  |  |  |  |  |  |  | 1 |
| Davidson 2004 |  | **√** |  |  |  |  |  |  |  |  |  |  |  |  |  |  |  |  |  |  |  |  |  |  |  |  |  |  |  | 1 |
| Deckers 2016 |  |  |  |  | **√** |  |  |  |  |  |  |  |  |  |  |  |  |  |  |  |  |  |  |  |  |  |  |  |  | 1 |
| De Craen 2006 |  |  |  |  |  |  |  |  |  |  |  |  | **√** |  |  |  |  |  |  |  |  |  |  |  |  |  |  |  |  | 1 |
| Deters 2013 |  |  |  | **√** |  |  |  |  |  |  |  |  |  |  |  |  |  |  |  |  |  |  |  |  |  |  |  |  |  | 1 |
| Diab 2014 |  |  |  |  | **√** |  |  |  |  |  |  | **√** |  |  |  |  |  |  |  |  |  |  |  |  |  |  |  |  |  | 2 |
| Dodds 2015 |  |  |  |  |  |  |  |  |  |  |  |  |  |  |  |  | **√** |  |  |  |  |  |  | **√** |  |  |  |  |  | 2 |
| Dodge 2015 |  |  |  |  |  |  |  |  |  |  |  |  | **√** |  |  |  |  |  |  |  |  |  |  |  |  | **√** |  |  |  | 2 |
| Dowd 2014 |  |  |  |  |  | **√** |  |  |  |  |  |  |  |  |  |  |  |  |  |  |  |  |  |  |  | **√** |  |  |  | 2 |
| Drenetea 2006 |  |  |  |  |  |  |  |  |  |  |  |  |  |  |  |  |  |  |  |  |  |  |  |  | **√** |  |  |  |  | 1 |
| Eggert 1995 |  |  |  |  |  |  |  |  |  |  |  |  |  |  |  | **√** |  |  |  |  |  |  |  |  |  |  |  |  |  | 1 |
| Ehlers 2017 |  |  |  |  |  |  |  |  |  |  |  |  | **√** |  |  |  |  |  |  |  |  |  |  |  |  |  |  |  |  | 1 |
| Elsherbiny 2018 |  |  |  |  |  |  |  |  |  |  |  |  |  |  |  |  |  |  |  |  |  |  |  |  |  | **√** |  |  |  | 1 |
| Estebsari 2018 |  |  |  |  |  |  |  |  |  |  |  |  | **√** |  |  |  |  |  |  |  |  |  |  |  |  |  |  |  |  | 1 |
| Evcik 2002 |  |  |  |  |  |  |  |  |  |  |  |  |  |  |  |  |  |  |  |  |  | **√** |  |  |  |  |  |  |  | 1 |
| Fokkema 2007 |  |  |  | **√** |  |  |  |  |  |  |  |  |  |  |  |  |  |  |  |  |  |  |  |  |  |  |  |  |  | 1 |
| Frankel 2010 |  |  |  |  | **√** |  |  |  |  |  |  | **√** |  |  |  |  |  |  |  |  |  |  |  |  |  |  |  |  |  | 2 |
| Fuki 2003 |  |  |  |  |  |  |  |  |  |  |  |  |  |  |  |  |  |  |  |  |  |  |  |  | **√** |  |  |  |  | 1 |
| Fukui 1993 |  |  |  | **√** |  |  |  |  |  |  |  |  |  |  |  |  |  |  |  |  |  |  |  |  |  |  |  |  |  | 1 |
| Fukui 2003 |  |  |  |  |  |  |  |  |  |  |  | **√** |  |  |  |  | **√** |  |  |  |  |  |  |  |  |  |  | **√** |  | 3 |
| Gantman 2012 |  |  |  |  | **√** |  |  |  |  |  |  | **√** |  |  |  |  |  | **√** |  |  |  |  |  |  |  |  |  | **√** |  | 4 |
| Gawrysiak 2009 |  |  |  |  |  |  |  |  |  |  |  |  |  |  |  | **√** |  |  |  |  |  |  |  |  |  |  |  |  |  | 1 |
| Gee 2019 |  |  |  |  |  | **√** |  |  |  |  |  |  |  |  |  |  |  |  |  |  |  |  |  |  |  |  |  |  |  | 1 |
| Gelkopf 1994 |  | **√** |  |  |  |  |  |  |  |  |  |  |  |  |  | **√** |  |  |  |  |  |  |  |  |  |  |  |  |  | 2 |
| Gilbody 2021 |  |  |  |  |  |  |  |  |  | **√** |  |  |  |  |  |  |  |  |  |  |  |  |  |  |  |  |  |  |  | 1 |
| Giovagnoli 2018 |  |  |  |  |  |  |  |  |  |  |  |  | **√** |  |  |  |  |  |  |  |  |  |  |  |  |  |  |  |  | 1 |
| Glynn 2004 |  | **√** |  |  |  |  |  |  |  |  |  |  |  |  |  |  |  |  |  |  |  |  |  |  |  |  |  |  |  | 1 |
| Graf 2002 |  |  |  | **√** |  |  |  |  |  |  |  |  |  |  |  |  |  |  |  |  |  |  |  |  |  |  |  |  |  | 1 |
| Granbom 2017 |  |  |  |  |  |  |  |  |  |  |  |  |  |  |  |  |  |  |  |  |  |  |  |  |  |  |  | **√** |  | 1 |
| Granholm 2005 |  | **√** |  |  |  |  |  |  |  |  |  |  |  |  |  |  |  |  |  |  |  |  |  |  |  |  |  |  |  | 1 |
| Gustafsson 2017 |  |  |  | **√** |  |  |  |  |  |  |  |  |  |  |  |  |  |  |  |  |  |  |  |  |  |  |  |  |  | 1 |
| Gustafson 2019 |  |  |  |  |  |  |  |  |  |  |  |  | **√** |  |  |  |  |  |  |  |  |  |  |  |  |  |  |  |  | 1 |
| Hall 1992 |  |  |  |  |  |  |  |  |  |  |  |  |  |  |  |  |  |  |  |  |  |  |  |  |  | **√** |  |  |  | 1 |
| Harris 1978 |  |  |  |  |  |  |  |  |  |  |  |  |  |  |  |  |  |  |  |  |  |  |  | **√** |  |  |  |  |  | 1 |
| Hartke 2003 |  |  |  |  |  |  |  | **√** |  |  |  |  | **√** |  |  |  |  |  |  |  |  |  |  |  |  |  |  |  |  | 2 |
| Haslam 2019 |  | **√** |  | **√** |  |  |  |  |  |  |  | **√** |  |  |  |  |  |  |  |  |  |  |  |  |  |  |  |  |  | 3 |
| Hasson-Ohayon 2007 |  |  |  |  |  |  |  |  |  |  |  |  |  |  |  | **√** |  |  |  |  |  |  |  |  |  |  |  |  |  | 1 |
| Hasson-Ohayon 2014 |  |  |  |  |  |  |  |  |  |  |  |  |  |  |  | **√** |  |  |  |  |  |  |  |  |  |  |  |  |  | 1 |
| Heckman 2006 |  |  |  |  |  |  |  |  |  |  |  | **√** |  |  |  |  |  |  |  |  |  |  |  |  |  |  |  |  |  | 1 |
| Heiney 2012 |  |  |  |  |  |  |  |  |  |  |  |  |  |  |  |  |  |  |  |  |  |  |  |  |  |  |  | **√** |  | 1 |
| Heller 1991 |  |  |  |  |  |  |  | **√** |  |  |  |  | **√** |  |  |  |  |  |  |  |  |  |  |  | **√** | **√** |  |  |  | 4 |
| Hind 2014 |  |  |  |  |  |  |  |  |  |  |  |  |  | **√** |  |  |  |  | **√** |  |  |  |  |  |  |  |  |  |  | 2 |
| Hopps 2003 |  |  |  | **√** |  |  |  |  |  |  |  |  |  |  |  |  |  |  |  |  |  |  |  |  |  |  |  |  |  | 1 |
| Huang 2011 |  |  |  |  |  |  |  |  |  |  |  |  | **√** |  |  |  |  |  |  |  |  |  |  |  |  |  |  |  |  | 1 |
| Iliffe 2014 |  |  |  |  |  |  |  |  |  |  |  |  |  |  |  |  |  |  |  |  |  |  |  |  |  | **√** |  |  |  | 1 |
| Interian 2016 |  |  |  |  |  |  |  |  |  |  |  |  |  |  |  | **√** |  |  |  |  |  |  |  |  |  |  |  |  |  | 1 |
| Jansons 2017 |  |  |  |  |  |  |  |  |  |  |  |  | **√** |  |  |  |  |  |  |  |  |  |  |  |  |  |  |  |  | 1 |
| Jarvis 2019 |  |  | **√** | **√** |  |  |  | **√** |  |  |  | **√** | **√** |  |  |  |  |  |  |  | **√** |  |  |  |  |  |  | **√** |  | 7 |
| Jazaieri 2012 |  |  |  |  |  |  |  |  |  |  |  |  |  |  |  |  |  |  |  |  |  |  |  | **√** |  |  |  |  |  | 1 |
| Jessen 1996 |  |  |  | **√** |  |  |  |  |  |  |  |  | **√** |  |  |  |  |  |  |  |  |  |  |  |  | **√** |  | **√** |  | 4 |
| Jing 2018 |  |  |  |  |  |  |  | **√** |  |  |  | **√** |  |  |  |  |  |  |  |  |  |  |  |  |  |  |  |  |  | 2 |
| Jing 2022 |  |  |  |  |  |  |  |  |  |  |  |  |  |  |  |  |  |  |  |  |  |  |  |  |  |  |  |  | **√** | 1 |
| Johnson 2020 |  |  |  |  |  |  |  |  |  |  |  |  | **√** |  |  |  |  |  |  |  |  |  |  |  |  |  |  |  |  | 1 |
| Jones 2019 |  |  |  |  |  |  |  |  |  |  |  |  | **√** |  |  |  |  |  |  |  |  |  |  |  |  |  |  |  |  | 1 |
| Joubert 2013 |  |  |  |  |  |  |  |  |  |  |  |  | **√** |  |  |  |  |  |  |  |  |  |  |  |  |  |  |  |  | 1 |
| Jung 2009 |  |  |  |  |  |  |  |  |  |  |  |  |  |  | **√** |  |  |  |  |  |  |  |  |  |  | **√** |  |  |  | 2 |
| Kahlbaugh 2011 |  |  |  |  |  |  | **√** |  |  |  |  |  |  |  | **√** |  |  |  |  |  |  |  |  |  |  | **√** |  |  |  | 2 |
| Kahlon 2021 |  |  |  |  |  |  |  | **√** |  | **√** |  |  |  |  |  |  |  |  |  |  |  |  |  |  |  |  |  |  |  | 2 |
| Kamegaya 2014 |  |  |  |  |  |  |  |  |  |  |  |  |  |  |  |  |  |  |  |  |  | **√** |  |  |  | **√** |  |  |  | 2 |
| Kapan 2017 |  |  |  |  |  |  |  |  |  |  |  |  | **√** |  |  |  |  |  |  |  |  |  |  |  |  |  |  |  |  | 1 |
| Kaplan 2011 |  |  |  |  |  |  |  |  |  |  |  |  |  |  |  | **√** |  |  |  |  |  |  |  |  |  |  |  |  |  | 1 |
| Kjøbli 2014 |  |  |  |  | **√** |  |  |  |  |  |  |  |  |  |  |  |  |  |  |  |  |  |  |  |  |  |  |  |  | 1 |
| Klingman 1993 |  |  |  |  | **√** |  |  |  |  |  |  |  |  |  |  |  |  |  |  |  |  |  |  |  |  |  |  |  |  | 1 |
| Kocak 2008 |  |  |  |  |  | **√** |  |  |  |  |  |  |  |  |  |  |  |  |  |  |  |  |  |  |  |  |  |  |  | 1 |
| Kremers 2006 |  |  |  | **√** |  |  |  |  |  |  |  | **√** | **√** |  |  |  |  |  |  |  |  |  |  |  | **√** | **√** |  | **√** |  | 6 |
| Käll 2020 |  |  |  | **√** |  |  |  |  |  |  |  | **√** |  |  |  |  |  |  |  |  |  |  |  |  |  |  |  | **√** |  | 3 |
| Lai 2020 |  |  |  |  |  |  |  | **√** |  |  |  | **√** |  |  |  |  |  |  |  |  |  |  |  |  | **√** |  |  |  |  | 3 |
| Lara 2016 |  |  | **√** |  |  |  |  |  |  |  |  |  |  |  |  |  |  |  |  |  |  |  |  |  |  |  |  |  |  | 1 |
| Larsen 2019 |  |  |  | **√** | **√** |  |  |  |  |  |  |  |  |  |  |  |  |  |  |  |  |  |  |  |  |  |  |  |  | 2 |
| Larsson 2016 |  |  |  | **√** |  |  |  |  |  |  |  |  | **√** |  |  |  |  |  |  |  | **√** |  |  |  |  |  |  |  |  | 3 |
| Leavitt 2019 |  |  |  | **√** |  |  |  |  |  |  |  |  |  |  |  |  |  |  |  |  |  |  |  |  |  |  |  |  |  | 1 |
| Lee 2019 |  |  |  |  |  |  |  |  |  |  |  |  |  |  |  |  |  |  |  |  |  |  |  | **√** |  |  |  |  |  | 1 |
| Leff 2009 |  |  |  |  | **√** |  |  |  |  |  |  |  |  |  |  |  |  |  |  |  |  |  |  |  |  |  |  |  |  | 1 |
| Li 2018 |  |  |  |  |  |  |  |  |  |  |  |  | **√** |  |  |  |  |  |  |  |  |  |  |  |  |  |  |  |  | 1 |
| Lindsay 2019 |  |  |  | **√** |  |  |  |  |  |  |  | **√** |  |  |  |  |  |  |  |  |  |  |  | **√** |  |  |  | **√** |  | 4 |
| Linhong 2020 |  |  |  |  |  |  |  |  |  |  |  |  |  |  |  |  |  |  |  |  |  |  |  |  |  |  |  |  | **√** | 1 |
| Lliffe 2014 |  |  |  |  |  |  |  |  |  |  |  |  |  |  |  |  |  |  |  |  |  | **√** |  |  |  |  |  |  |  | 1 |
| Lloyd-Evans 2020 |  | **√** |  |  |  |  |  |  |  |  |  | **√** |  |  |  |  |  |  |  |  |  |  |  |  |  |  |  |  |  | 2 |
| Lokk 1990 |  |  |  |  |  |  |  |  |  |  |  |  |  |  |  |  |  |  |  |  |  |  |  |  | **√** | **√** |  |  |  | 2 |
| Loucks 2020 |  |  |  |  |  | **√** |  |  |  |  |  | **√** |  |  |  |  |  |  |  |  |  |  |  |  |  |  |  |  |  | 2 |
| Lu 2020 |  |  |  |  |  | **√** |  |  |  |  |  |  |  |  |  |  |  |  |  |  |  |  |  |  |  |  |  |  | **√** | 2 |
| MacIntyre 1999 |  |  |  |  |  |  |  |  |  |  |  |  | **√** |  |  |  |  |  |  |  |  |  |  |  | **√** |  |  |  |  | 2 |
| MacIntyre 2002 |  |  |  |  |  |  |  |  |  |  |  |  |  |  |  |  |  |  |  |  |  |  | **√** |  |  |  |  |  |  | 1 |
| Maki 2012 |  |  |  |  |  |  |  |  |  |  |  |  |  |  |  |  |  |  |  |  |  | **√** |  |  |  | **√** |  |  |  | 2 |
| Marashian 2012 |  |  |  | **√** |  |  |  |  |  |  |  |  |  |  |  |  |  |  |  |  |  |  |  |  |  |  |  |  |  | 1 |
| Marder 1996 |  | **√** |  |  |  |  |  |  |  |  |  |  |  |  |  |  |  |  |  |  |  |  |  |  |  |  |  |  |  | 1 |
| Margalit 1995 |  |  |  |  | **√** |  |  |  |  |  |  |  |  |  |  |  |  |  |  |  |  |  |  |  |  |  |  |  |  | 1 |
| Markle-Reid 2006 |  |  |  |  |  |  |  |  |  |  |  |  | **√** |  |  |  |  |  |  |  |  |  |  |  |  |  |  |  |  | 1 |
| Marzillier 1976 |  |  |  |  |  |  |  |  |  |  |  |  |  |  |  | **√** |  |  |  |  |  |  |  |  |  |  |  |  |  | 1 |
| Mascaro 2016 |  |  |  |  |  |  |  |  |  |  |  | **√** |  |  |  |  |  |  |  |  |  |  |  |  |  |  |  |  |  | 1 |
| Mascaro 2018 |  |  |  |  |  | **√** |  |  |  |  |  |  |  |  |  |  |  |  |  |  |  |  |  | **√** |  |  |  |  |  | 2 |
| Mason 2016 |  |  |  |  | **√** |  |  |  |  |  |  |  |  |  |  |  |  |  |  |  |  |  |  |  |  |  |  |  |  | 1 |
| Massia-Warner 2005 |  |  |  |  | **√** |  |  |  |  |  |  |  |  |  |  | **√** |  |  |  |  |  |  |  |  |  |  |  |  |  | 2 |
| Mattanah 2010 |  |  |  | **√** | **√** | **√** |  |  |  |  |  |  |  |  |  |  |  |  |  |  |  |  |  |  |  |  |  |  |  | 3 |
| Matthews 2018 |  |  |  |  | **√** |  |  |  |  |  |  | **√** |  |  |  |  |  | **√** |  |  |  |  |  |  |  |  |  |  |  | 3 |
| Matz-Costa 2018 |  |  |  |  |  |  |  |  |  |  | **√** |  |  |  |  |  |  |  |  |  |  |  |  |  |  |  |  |  |  | 1 |
| McAuley 2000 |  |  |  |  |  |  |  |  |  |  |  |  | **√** |  |  |  |  |  |  |  |  |  |  |  |  | **√** |  |  |  | 2 |
| McWirther 1996 |  |  |  |  |  | **√** |  |  |  |  |  |  |  |  |  |  |  |  |  |  |  |  |  |  |  | **√** |  | **√** |  | 3 |
| Mendelson 2013 |  |  |  |  |  |  |  |  |  |  |  |  |  |  |  | **√** |  |  |  |  |  |  |  |  |  |  |  |  |  | 1 |
| Moieni 2020 |  |  |  |  |  |  |  |  |  |  |  |  | **√** |  |  |  |  |  |  |  |  |  |  |  |  |  |  |  |  | 1 |
| Morgenstern 2015 |  |  |  |  |  |  |  |  |  |  |  |  | **√** |  |  |  |  |  |  |  |  |  |  |  |  |  |  |  |  | 1 |
| Morrow 1998 |  |  |  |  |  |  |  |  |  |  |  |  |  |  |  |  |  |  |  |  |  |  |  |  | **√** |  |  |  |  | 1 |
| Morton 2018 |  |  |  |  |  |  |  |  |  |  |  |  | **√** |  |  |  |  |  |  |  | **√** |  |  |  |  |  | **√** |  |  | 3 |
| Mountain 2014 |  |  |  |  |  |  | **√** | **√** |  |  |  |  | **√** |  |  |  |  |  |  |  |  |  | **√** |  | **√** | **√** |  |  |  | 6 |
| Mountain 2017 |  |  |  |  |  |  |  |  |  |  |  |  | **√** |  |  |  |  |  |  |  |  |  |  |  |  | **√** |  |  |  | 2 |
| Mutrie 2012 |  |  |  |  |  |  |  |  |  |  |  |  |  |  |  |  |  |  |  |  |  | **√** |  |  |  | **√** |  |  |  | 2 |
| Neil-Sztramko 2020 |  |  |  |  |  |  |  |  |  |  |  |  |  |  |  |  |  |  |  |  |  |  |  |  |  |  | **√** |  |  | 1 |
| Nelson 2019 |  |  |  |  |  |  |  | **√** |  |  |  |  | **√** |  |  |  | **√** |  |  |  |  |  |  |  |  |  |  |  |  | 3 |
| Ollonqvist 2008 |  |  |  | **√** |  |  |  |  | **√** |  |  |  | **√** |  |  |  |  |  |  |  |  | **√** |  |  | **√** |  |  |  |  | 5 |
| O'Mahen 2014 |  |  |  |  |  |  |  |  |  |  |  |  |  |  |  | **√** |  |  |  |  |  |  |  |  |  |  |  |  |  | 1 |
| Orchard 1986 |  |  |  | **√** |  | **√** |  |  |  |  |  |  |  |  |  |  |  |  |  |  |  |  |  |  |  |  |  |  |  | 2 |
| Pandya 2019 |  |  |  |  |  |  |  |  |  |  |  |  |  |  |  |  |  |  |  |  |  |  |  | **√** |  |  |  |  |  | 1 |
| Parry 2016 |  |  |  |  |  |  |  |  |  |  |  |  | **√** |  |  |  |  |  |  |  |  |  |  |  |  |  |  |  |  | 1 |
| Pos 2019 |  | **√** |  |  |  |  |  |  |  |  |  |  |  |  |  |  |  |  |  |  |  |  |  |  |  |  |  |  |  | 1 |
| Pot-Kolder 2018 |  | **√** |  |  |  |  |  |  |  |  |  |  |  |  |  |  |  |  |  |  |  |  |  |  |  |  |  |  |  | 1 |
| Priebe 2020 |  | **√** |  |  |  |  |  |  |  |  |  |  |  |  |  |  |  |  |  |  |  |  |  |  |  |  |  |  |  | 1 |
| Purohit 2016 |  |  |  |  | **√** |  |  |  |  |  |  |  |  |  |  |  |  |  |  |  |  |  |  |  |  |  |  |  |  | 1 |
| Pynnonen 2018 |  |  |  |  |  |  |  |  |  |  |  |  | **√** |  |  |  |  |  |  |  |  |  |  |  |  |  |  |  |  | 1 |
| Quayle 2001 |  |  |  |  | **√** |  |  |  |  |  |  |  |  |  |  |  |  |  |  |  |  |  |  |  |  |  |  |  |  | 1 |
| Ransom 2008 |  |  |  |  |  |  |  |  |  |  |  | **√** |  |  |  |  |  |  |  |  |  |  |  |  |  |  |  |  |  | 1 |
| Rantanen 2015 |  |  |  |  |  |  |  |  |  |  |  |  |  |  |  |  |  |  |  |  |  |  | **√** |  |  |  |  |  |  | 1 |
| Regev 2005 |  |  |  |  | **√** |  |  |  |  |  |  |  |  |  |  |  |  |  |  |  |  |  |  |  |  |  |  |  |  | 1 |
| Rigney et al 2017 |  |  |  |  |  |  |  |  |  |  |  |  |  |  |  |  | **√** |  |  |  |  |  |  |  |  |  |  |  |  | 1 |
| Ristolainen 2020 |  |  |  |  |  |  |  |  |  |  |  |  |  |  |  |  |  |  |  |  |  |  |  |  | **√** |  |  |  |  | 1 |
| Rivera 2007 |  | **√** |  |  |  |  |  |  |  |  |  |  |  |  |  | **√** |  |  |  |  |  |  |  |  |  |  |  |  |  | 2 |
| Roberts 2014 |  | **√** |  |  |  |  |  |  |  |  |  |  |  |  |  |  |  |  |  |  |  |  |  |  |  |  |  |  |  | 1 |
| Robinson 2013 | **√** |  |  | **√** |  |  |  |  |  |  |  |  | **√** |  |  |  |  |  | **√** | **√** |  |  |  |  |  | **√** |  | **√** |  | 7 |
| Rodriguez-Rom. 2020 |  |  |  |  |  |  |  |  |  |  |  |  |  |  |  |  |  |  |  |  |  |  |  |  |  |  |  | **√** |  | 1 |
| Rohde 2004 |  |  |  |  | **√** |  |  |  |  |  |  |  |  |  |  |  |  |  |  |  |  |  |  |  |  |  |  |  |  | 1 |
| Rook 2003 |  |  |  |  |  |  |  |  |  |  |  |  | **√** |  |  |  |  |  |  |  |  |  |  |  |  |  |  | **√** |  | 2 |
| Rotondi 2005 |  |  |  |  |  |  |  |  |  |  |  |  |  |  |  | **√** |  |  |  |  |  |  |  |  |  |  |  |  |  | 1 |
| Routasalo 2008 |  |  |  |  |  |  |  |  | **√** |  |  |  |  |  |  |  |  |  |  |  |  |  |  |  |  |  |  |  |  | 1 |
| Routasalo 2009 |  |  |  | **√** |  |  |  |  |  |  |  |  | **√** |  |  |  |  |  |  |  |  |  |  |  | **√** |  |  |  |  | 3 |
| Saito 2012 |  |  |  |  |  |  |  |  | **√** |  |  |  | **√** |  |  |  |  |  | **√** |  |  |  |  |  | **√** | **√** |  |  |  | 5 |
| Samarel 2002 |  |  |  |  |  |  |  |  |  |  |  |  |  |  |  |  | **√** |  |  |  |  |  |  |  |  |  |  |  |  | 1 |
| Samhkaniyan 2015 |  |  |  | **√** |  |  |  |  |  |  |  |  |  |  |  |  |  |  |  |  |  |  |  |  |  |  |  |  |  | 1 |
| Samulski 2004 |  |  |  | **√** |  |  |  |  |  |  |  |  |  |  |  |  |  |  |  |  |  |  |  |  |  |  |  |  |  | 1 |
| Sanchez 2017 |  |  |  |  | **√** |  |  |  |  |  |  |  |  |  |  |  |  |  |  |  |  |  |  |  |  |  |  |  |  | 1 |
| Saulsberry 2013 |  |  |  |  |  |  |  |  |  |  |  |  |  |  |  |  |  | **√** |  |  |  |  |  |  |  |  |  |  |  | 1 |
| Savelkoul 2003 |  |  |  |  |  |  |  |  |  |  |  |  |  |  |  |  |  |  |  |  |  |  |  |  | **√** |  |  |  |  | 1 |
| Sayied 2015 |  |  |  | **√** |  |  |  |  |  |  |  |  |  |  |  |  |  |  |  |  |  |  |  |  |  |  |  |  |  | 1 |
| Schene 1993 |  |  |  |  |  |  |  |  |  |  |  |  |  |  |  | **√** |  |  |  |  |  |  |  |  |  |  |  |  |  | 1 |
| Schulz 1976 |  |  |  |  |  |  |  |  |  |  |  |  |  |  |  |  |  |  |  |  |  |  |  |  |  | **√** |  |  |  | 1 |
| Schwinden 2014 |  |  |  | **√** |  |  |  |  |  |  |  |  |  |  |  |  |  |  |  |  |  |  |  |  |  |  |  |  |  | 1 |
| Shapira 2007 |  |  |  |  |  |  | **√** | **√** |  |  |  |  |  | **√** |  |  |  |  |  |  |  |  |  |  |  |  |  |  |  | 3 |
| Shapira 2021 |  |  |  |  |  |  |  | **√** |  | **√** |  |  |  |  |  |  |  |  |  |  |  |  |  |  |  |  |  |  |  | 2 |
| Shaui 1981 |  |  |  | **√** |  |  |  |  |  |  |  |  |  |  |  |  |  |  |  |  |  |  |  |  |  |  |  |  |  | 1 |
| Sheridan 2015 |  | **√** |  |  |  |  |  |  |  |  |  |  |  |  |  |  |  |  |  |  |  |  |  |  |  |  |  | **√** |  | 2 |
| Shije 2022 |  |  |  |  |  |  |  |  |  |  |  |  |  |  |  |  |  |  |  |  |  |  |  |  |  |  |  |  | **√** | 1 |
| Shima 2016 |  |  |  | **√** |  |  |  |  |  |  |  |  |  |  |  |  |  |  |  |  |  |  |  |  |  |  |  |  |  | 1 |
| Shvedko 2020 |  |  |  |  |  |  |  |  |  |  |  |  |  |  |  |  |  |  |  |  |  |  |  |  |  |  |  | **√** |  | 1 |
| Sidner 2018 |  |  |  |  |  |  |  |  |  |  |  |  | **√** |  |  |  |  |  |  |  |  |  |  |  |  |  |  |  |  | 1 |
| Silverman 2014 |  | **√** |  |  |  |  |  |  |  |  |  |  |  |  |  | **√** |  |  |  |  |  |  |  |  |  |  |  |  |  | 2 |
| Slegers 2007 |  |  |  |  |  |  | **√** |  |  |  |  |  |  |  |  |  |  |  |  |  |  |  |  |  |  |  |  |  |  | 1 |
| Slegers 2008 |  |  |  |  |  |  | **√** | **√** |  |  |  |  | **√** | **√** |  |  |  |  |  |  |  |  |  |  | **√** | **√** | **√** |  |  | 7 |
| Sollami 2017 |  |  |  |  |  |  |  |  |  |  |  |  | **√** |  |  |  |  |  |  | **√** |  |  |  |  |  |  |  |  |  | 2 |
| Solomon 1995 |  |  |  |  |  |  |  |  |  |  |  |  |  |  |  | **√** |  |  |  |  |  |  |  |  |  |  |  |  |  | 1 |
| Stice 2010 |  |  |  |  | **√** |  |  |  |  |  |  |  |  |  |  |  |  |  |  |  |  |  |  |  |  |  |  |  |  | 1 |
| Stravynski 1982 |  |  |  |  |  |  |  |  |  |  |  |  |  |  |  | **√** |  |  |  |  |  |  |  |  |  |  |  |  |  | 1 |
| Struchen 2011 |  |  |  |  |  |  |  |  |  |  |  |  |  |  |  |  |  |  |  |  |  |  |  |  |  |  |  | **√** |  | 1 |
| Tabrize 2016 |  |  |  | **√** |  |  |  |  |  |  |  | **√** |  |  |  |  | **√** |  |  |  |  |  |  |  |  |  |  | **√** |  | 4 |
| Taube 2018 |  |  |  | **√** |  |  |  |  |  |  |  |  | **√** |  |  |  |  |  |  |  |  |  |  |  |  |  |  |  |  | 2 |
| Terzian 2013 |  | **√** |  |  |  |  |  |  |  |  |  |  |  |  |  | **√** |  |  |  |  |  |  |  |  |  |  |  |  |  | 2 |
| Thamboo 2016 |  |  |  | **√** |  |  |  |  |  |  |  |  |  |  |  |  |  |  |  |  |  |  |  |  |  |  |  |  |  | 1 |
| Theeke 2016 |  |  |  | **√** |  |  |  |  |  |  |  | **√** | **√** |  |  |  |  |  |  |  |  |  |  |  |  |  |  | **√** |  | 4 |
| Thomas 2016 |  |  |  | **√** |  |  |  |  |  |  |  |  |  |  |  |  |  |  |  |  |  |  |  |  |  |  |  | **√** |  | 2 |
| Tsai 2010 |  |  |  | **√** |  |  |  |  |  |  |  |  |  |  |  |  |  |  |  |  |  |  |  |  |  | **√** |  | **√** |  | 3 |
| Tsai 2011 |  |  |  | **√** |  |  |  |  |  |  |  |  | **√** | **√** |  |  |  |  |  |  |  |  |  |  |  | **√** |  |  |  | 4 |
| Tsai 2015 |  |  |  |  |  |  |  |  |  |  |  |  |  | **√** |  |  |  |  |  |  |  |  |  |  |  |  |  |  |  | 1 |
| Tsai 2020 |  |  |  | **√** |  |  |  | **√** |  |  |  |  | **√** | **√** |  |  |  |  |  |  |  |  |  |  |  |  |  | **√** |  | 5 |
| Tse 2010 |  |  |  |  |  |  |  |  |  |  |  |  |  |  |  |  |  |  |  |  |  |  |  |  |  | **√** |  | **√** |  | 2 |
| Tse 2012 |  |  |  |  |  |  |  |  |  |  |  |  | **√** |  |  |  |  |  |  |  |  |  |  |  |  |  |  |  |  | 1 |
| Tse 2013 |  |  |  |  |  |  |  |  |  |  |  |  | **√** |  |  |  |  |  |  |  |  |  |  |  |  |  |  |  |  | 1 |
| Tse 2014 |  |  |  |  |  |  |  |  |  |  |  |  | **√** |  |  |  |  |  |  | **√** |  |  |  |  |  |  |  |  |  | 2 |
| Tse 2016 |  |  |  |  |  |  |  |  |  |  |  |  | **√** |  |  |  |  |  |  |  |  |  |  |  |  |  |  |  |  | 1 |
| Van Gestel 2012 |  |  |  |  |  |  |  |  |  |  |  | **√** |  |  |  |  |  |  |  |  |  |  |  |  |  |  |  |  |  | 1 |
| van Rossum 1993 |  |  |  |  |  |  |  |  |  |  |  |  |  |  |  |  |  |  |  |  |  |  |  |  |  | **√** |  |  |  | 1 |
| Vanoh 2019 |  |  |  |  |  |  |  |  |  |  | **√** |  |  |  |  |  |  |  |  |  |  |  |  |  |  |  |  |  |  | 1 |
| Vassilopoulos 2018 |  |  |  |  | **√** |  |  |  |  |  |  |  |  |  |  |  |  |  |  |  |  |  |  |  |  |  |  |  |  | 1 |
| Walshe 2016 |  |  |  |  |  |  |  |  |  |  |  |  | **√** |  |  |  |  |  |  |  |  |  | **√** |  |  |  |  |  |  | 2 |
| Wan 2017 |  |  |  |  |  |  |  |  |  |  |  |  | **√** |  |  |  |  |  |  |  |  |  |  |  |  |  |  |  |  | 1 |
| Wang 2010 |  |  |  |  |  |  |  |  |  |  |  |  | **√** |  |  |  |  |  |  |  |  |  |  |  |  |  |  |  |  | 1 |
| Westerhof 2017 |  |  |  |  |  |  |  |  |  |  |  |  | **√** |  |  |  |  |  |  | **√** |  |  |  |  |  |  |  |  |  | 2 |
| Westerhof 2018 |  |  |  |  |  |  |  |  |  |  |  |  |  |  |  |  |  |  |  |  |  |  |  |  |  | **√** |  |  |  | 1 |
| White 2002 |  |  |  |  |  |  | **√** |  |  |  |  |  | **√** |  |  |  |  |  |  |  |  |  |  |  | **√** | **√** |  | **√** |  | 5 |
| Winstead 2014 |  |  |  | **√** |  |  |  |  |  |  |  |  |  |  |  |  |  |  |  |  |  |  |  |  |  | **√** |  |  |  | 2 |
| Wood 1984 |  |  |  | **√** |  |  |  |  |  |  |  |  |  |  |  |  |  |  |  |  |  |  |  |  |  |  |  |  |  | 1 |
| Woodward 2011 |  |  |  |  |  |  | **√** |  |  |  |  |  | **√** |  |  |  |  |  |  |  |  |  |  |  |  | **√** |  |  |  | 3 |
| Wu 2015 |  |  |  |  |  |  |  |  |  |  |  |  |  |  | **√** |  |  |  |  |  |  |  |  |  |  |  |  |  |  | 1 |
| Xiao, T. 2021 |  |  |  |  |  | **√** |  |  |  |  |  |  |  |  |  |  |  |  |  |  |  |  |  |  |  |  |  |  | **√** | 2 |
| Xiao, Z. 2021 |  |  |  |  |  |  |  |  |  |  |  |  |  |  |  |  |  |  |  |  |  |  |  |  |  |  |  |  | **√** | 1 |
| Xu 2016 |  |  |  |  |  |  |  |  |  |  |  |  |  |  | **√** |  |  |  |  |  |  |  |  |  |  |  |  |  |  | 1 |
| Yap 2017 |  |  |  |  |  |  |  |  |  |  |  |  | **√** |  |  |  |  |  |  |  |  |  |  |  |  |  |  |  |  | 1 |
| Yi 2012 |  |  |  |  |  |  |  |  |  |  |  |  |  |  |  |  |  |  |  |  |  |  |  |  | **√** |  |  |  |  | 1 |
| Yousefipour 2021 |  |  |  |  |  | **√** |  |  |  |  |  |  |  |  |  |  |  |  |  |  |  |  |  |  |  |  |  |  |  | 1 |
| Yu 2019 |  |  |  |  |  |  |  |  |  |  | **√** |  |  |  |  |  |  |  |  |  |  |  |  |  |  |  |  |  |  | 1 |
| Zang 2013 |  |  |  |  |  |  |  |  |  |  |  |  |  |  |  | **√** |  |  |  |  |  |  |  |  |  |  |  |  |  | 1 |
| Zang 2014 |  |  |  |  |  |  |  |  |  |  |  |  |  |  |  | **√** |  |  |  |  |  |  |  |  |  |  |  |  |  | 1 |
| Zara 2017 |  |  |  |  |  |  |  |  |  |  |  | **√** |  |  |  |  |  |  |  |  |  |  |  |  |  |  |  |  |  | 1 |
| Zhang 2016 |  |  |  |  | **√** |  |  |  |  |  |  |  |  |  |  |  |  |  |  |  |  |  |  |  |  |  |  |  |  | 1 |
| Zhang 2018 |  |  |  | **√** |  | **√** |  |  |  |  |  | **√** |  |  |  |  |  | **√** |  |  |  |  |  | **√** |  | **√** |  | **√** |  | 7 |
| Zhengkuan 2021 |  |  |  |  |  |  |  |  |  |  |  |  |  |  |  |  |  |  |  |  |  |  |  |  |  |  |  |  | **√** | 1 |

**Appendix 7**. Results from “other” types of interventions

**Table.** Characteristics of the reviews on **mixed/other** interventions

| **Author year**  **Outcome**  **Population details** | **Intervention details (number of studies)** | | **Findings**  **Meta-analysis: Effect sizes (95% CI), subgroup analysis**  **Narrative synthesis: Report of significant effects** |
| --- | --- | --- | --- |
|  | **Intervention vs. comparator** | **Delivery: group vs. individual**  **Mode: F2F, internet, etc.**  **Frequency/duration (F/D)**  **Follow-up (FU)** |  |
| Reviews with meta-analysis | |  | |
| Eccles 2021  Loneliness  Adolescents (age 11-16) at-risk (orphan, learning disorder) | Learning new hobby (examples NR)  vs. NR | Delivery: Group  Mode: F2F  F/D: 1-4 times per week//12-25w  FU: No | g 0.47 (-0.05; 0.99), *I^2^* NR, 2 studies (n = 118). |
| Hoang 2022  Social isolation, loneliness  Age 65+ | Multicomponent interventions (e.g., support and psychoeducation)  vs. NR | Delivery: Group, individual  Mode: NR  F/D: NR/3-38w  FU: No | Social isolation (community-dwelling): SMD 0.29 (0.15; 0.43), *I^2^* = 0%, 6 studies  Loneliness (community-dwelling): SMD -0.67 (-1.13; -0.21), *I^2^* = 0%, 2 studies  Loneliness (LTC): SMD -0.53 (-0.86; -0.20), *I^2^* = 57%, 3 studies |
| Hoang 2022  Social isolation, loneliness  Age 65+ | Exercise (e.g., dance, yoga, Tai Chi, strength and balance)  vs. TAU (3), other activity (5) | Delivery: Group  Mode: F2F  F/D: NR/4-26w  FU: No | Social isolation (community): SMD -0.12 (-0.55; 0.31), 1 study  Loneliness (community-dwelling): SMD -0.15 (-0.44; 0.15), *I^2^* = 35%, 5 studies  Loneliness (LTC): SMD -0.53 (-0.86; -0.20), *I^2^* = 57%, 3 studies. (n’s = NR) |
| Hoang 2022  Loneliness and social isolation  Age 65+ | Music (e.g., music therapy, choir program)  vs. Waitlist (3), other activity (1) | Delivery: Group  Mode: F2F  F/D: NR/6-24w  FU: No | Social isolation: SMD -0.11 (-0.57; 0.35), *I^2^* = 0%, 2 studies  Loneliness: SMD -0.34 (-0.55; -0.13), 1 study  (n’s = NR) |
| Svedko 2018  Social network, social isolation, loneliness  Community-dwelling older adults (age 51-82). | Physical activity with social interactions (e.g., health education, CBT, lectures, nurse counselling).  vs. NR | Delivery: Mostly groups  Mode: NR  F/D: NR/12w  FU: 6-12m | Social network: SMD -0.00 (-0.28; 0.27), *I^2^* = 68%, 4 studies.  Narrative synthesis showed no evidence of effect for loneliness (n=3) or social isolation (n=1).  (n’s = NR) |
| Zhang 2023  Loneliness  Problematic mobile phone use (mean age 14-21) | Combination of exercise (basketball, Tai Chi, Qigong) and psychological (CBT, mindfulness)  vs. NR | Delivery: NR  Mode: NR  F/D: 1-2 per w/4-12w  FU: NR | SMD -1.10 (-1.45; -0.71), *I^2^* =75%, 8 studies (n=1,107) |
| Reviews with narrative synthesis | |  | |
| Williams 2021  Loneliness  NR | Health and social care provision compatible with COVID-19 social distancing (support from health or social care professionals).  vs. NR | Delivery: NR  Mode: NR  F/D: NR  FU: NR | No evidence of effect. 2 studies (n’s = NR). |
| Williams 2021  Social isolation, loneliness  NR | Leisure/skill development compatible with COVID-19 social distancing: Provide leisure activities or promote learning a new skill (exercise, computer training, video gaming, gardening, general activities).  vs. NR | Delivery: NR  Mode: NR  F/D: NR  FU: NR | Social isolation (2/6 found evidence of effect):  Evidence of effect found for 2 poor/fair quality trials (gardening, general activities). No evidence of effect found for 1 good and 2 fair quality exercise, 1 fair quality computer training intervention.  Loneliness (3/11 found evidence of effect):  Evidence of effect found for 2 fair quality video gaming, 1 fair quality gardening trial. No evidence of effect found for 1 good and 3 fair quality exercise, 3 fair quality computer training, 1 fair quality general activity intervention. |

Notes: ^1^ Not limited to a specific group. “Effect” indicates a significant (p< .05) effect in favor of the intervention. Abbrevations: F2F = Face-to-face, TAU = treatment as usual. W = weeks, M = months, Y = years, ES = Effect size, N = number of participants, g = Hedges’ g, SMS = standardized mean difference, NR = not reported, RCT = Randomized controlled trial, SI = Social isolation, L = Loneliness, ICT = Information and communications technology, LTC = Long-term care.

**Table**. Summary of findings on the efficacy of **other** interventions to reduce social isolation and loneliness.

|  | **Study details** | | | | | **General conclusion** | |
| --- | --- | --- | --- | --- | --- | --- | --- |
|  | **Intervention type** | **Sample** | **N** | **# RCTs** | **Reference** | **Social isolation** | **Loneliness** |
| Meta-analysis | Hobby/skill development | Young people | 118 | 2 | Eccles 2021 |  | g 0.47 (-0.05; 0.99), *I^2^* = NR |
|  | Therapy, exercise | Young people | 1,107 | 8 | Zhang 2023 |  | Large SMD -1.10 (-1.45; -0.71), *I^2^* = 75% |
|  | Therapy, exercise | Older adults | NR | SI 4, L 3 | Svedko 2018 | SMD 0.00 (-0.28; 0.27), *I^2^* = 68% |  |
|  | Multicomponent | Age 65+ | NR | SI 6, L 2 | Hoang 2022 | Low SMD 0.29 (0.15; 0.43), *I^2^* = 0% | Mod. SMD -0.67 (-1.13; -0.21), *I^2^* = NR |
|  | Multicomponent | Age 65+, LTC | NR | 3 | Hoang 2022 |  | Mod. SMD -0.53 (-0.86; -0.20), *I^2^* = 57% |
|  | Exercise, group-based | Age 65+ | NR | SI 1, L 5 | Hoang 2022 | SMD -0.12 (-0.55; 0.31), *I^2^* = NR | SMD -0.15 (-0.44; 0.14), *I^2^* = 35% |
|  | Music (choir, therapy) | Age 65+ | NR | SI 2, L 1 | Hoang 2022 | SMD -0.11 (-0.57; 0.35), *I^2^* = 0% | SMD -0.34 (-0.55; -0.13), *I^2^* = NR |
| Narrati. | Health/social care services | NR | NR | 2 | Williams 2021 |  |  |
|  | Leisure/skill development | NR | NR | SI 6, L 11 | Williams 2021 |  |  |

Color keys: green = evidence of effect, yellow = inconsistent or inconclusive evidence of effect, red = no evidence of effect.

**References**

1. Beckers A, Buecker S, Casabianca EJ, Minna N. Effectiveness of interventions tackling loneliness, EUR 31313 EN, Publications Office of the European Union, Luxembourg, 2022, ISBN 978-92-76-59108-5, doi:10.2760/277109, JRC130944.; 2022. Report No.: 9276591087.

2. Boulton E, Kneale D, Stansfield C, Heron P, Hanratty B, McMillan D, et al. Rapid review of reviews: what remotely delivered interventions can reduce social isolation and loneliness among older adults. F1000Research 2021, 9:1368 (<https://doi.org/10.12688/f1000research.27076.2>). 2020.

3. Chipps J, Jarvis MA, Ramlall S. The effectiveness of e-Interventions on reducing social isolation in older persons: A systematic review of systematic reviews. Journal of Telemedicine and Telecare. 2017;23(10):817-27.

4. Jarvis M-A, Padmanabhanunni A, Balakrishna Y, Chipps J. The effectiveness of interventions addressing loneliness in older persons: an umbrella review. International Journal of Africa Nursing Sciences. 2020;12:100177.

5. Veronese N, Galvano D, D’Antiga F, Vecchiato C, Furegon E, Allocco R, et al. Interventions for reducing loneliness: An umbrella review of intervention studies. Health & social care in the community. 2021;29(5):e89-e96.

6. Victor C, Mansfield L, Kay T, Daykin N, Lane J, Duffy LG, et al. An overview of reviews: the effectiveness of interventions to address loneliness at all stages of the life-course. What Works Wellbeing; 2018.

1. Our inclusion criteria did not fully match the AMSTAR2 criteria, as our inclusion criteria equals a “partial yes” in AMSTAR2. [↑](#footnote-ref-1)
